# Coumarin–Thiourea Hybrids: Structural Features Governing CA Inhibition and Antiproliferative Effects

**DOI:** 10.3390/ijms27093743

**Published:** 2026-04-23

**Authors:** Alma Fuentes-Aguilar, Rebecca Colombo, Aday González-Bakker, Adrián Puerta, Penélope Merino-Montiel, Sara Montiel-Smith, José L. Vega-Báez, Simone Giovannuzzi, Alessio Nocentini, José G. Fernández-Bolaños, Claudiu T. Supuran, José M. Padrón, Óscar López

**Affiliations:** 1 Departamento de Química Orgánica, Facultad de Química, Universidad de Sevilla, Apartado 1203, E-41071 Seville, Spainbolanos@us.es (J.G.F.-B.); 2 Facultad de Ciencias Químicas, Ciudad Universitaria, Benemérita Universidad Autónoma de Puebla, Puebla 72570, Mexicomaria.montiel@correo.buap.mx (S.M.-S.);; 3 NEUROFARBA Department, Sezione di Scienze Farmaceutiche e Nutraceutiche, University of Florence, 50019 Florence, Italyclaudiu.supuran@unifi.it (C.T.S.); 4 BioLab, Instituto Universitario de Bio-Orgánica “Antonio González” (IUBO-AG), Universidad de La Laguna, c/Astrofísico Francisco Sánchez 2, E-38206 La Laguna, Spainjmpadron@ull.es (J.M.P.)

**Keywords:** coumarins, thioureas, carbonic anhydrases, enzyme inhibition, antiproliferative activity, docking simulations

## Abstract

Selective inhibition of the tumour-associated carbonic anhydrase (CA) isoforms IX and XII, which are overexpressed in hypoxic tumours, has emerged as a promising strategy for the development of novel anticancer agents. Among the diverse CA inhibitors reported to date, coumarins have attracted particular attention. These chromenone derivatives, widely distributed in phytochemicals, display a broad range of biological activities and are known to act as *suicide inhibitors* of CAs. Following the tail approach, we designed a series of hybrid compounds combining a coumarin core with an *N*-arylthioureido scaffold located at the C-7 position and investigated how structural variations—including substituents on the coumarin and aromatic moieties, tether length, and urea/thiourea isosterism—influence their biological properties (CA inhibition and antiproliferative activity). Substituted coumarins at C-3 and C-4 were efficiently prepared via Pechmann condensation, while the thioureido motif was introduced using various aryl isothiocyanates as key synthetic intermediates. The lead compound, featuring a dimethylated coumarin, a pentyl linker, and an *N*-(*p*-tolyl)thioureido residue, inhibited the target enzymes in the low- to mid-nanomolar range (*K*_i_ = 6.0 and 49.9 nM, respectively), displaying selectivity indexes (S.I.s) surpassing those of the reference drug acetazolamide (AAZ). Moreover, it exhibited potent antiproliferative activity, with GI_50_ values in the low micromolar range (1.9–3.5 µM) against both drug-sensitive and multidrug-resistant cancer cell lines. Label-free three-dimensional holotomographic microscopy revealed that this compound triggers slow apoptosis, leading to cell death after approximately 20 h of exposure.

## 1. Introduction

Carbonic anhydrases (CAs, EC 4.2.1.1), first identified more than 90 years ago [1], constitute a large superfamily of metalloenzymes within the hydrolase class [2]. These enzymes catalyze the reversible hydration of CO_2_ to yield HCO_3_^−^ plus a proton [3]. Despite the apparent simplicity of this chemical transformation, CAs participate in an exceptionally broad spectrum of essential biological processes [4]. These include respiration, where erythrocytic isoforms regulate gas exchange [5], acid-base homeostasis [6], photosynthesis [7], plant growth [8], and electrolyte secretion [9], as well as key metabolic pathways such as gluconeogenesis, ureagenesis, and de novo lipogenesis [10,11,12,13]. Notably, CAs rank among the most efficient biocatalysts known (*k*_cat_ = 10^4^–10^6^ s^−1^) [14], providing the required CO_2_/HCO_3_^−^ flux to sustain cellular activity, whereas the uncatalyzed hydration of CO_2_ is far too slow (*k*_cat_ = 0.15 s^−1^) [15].

Beyond their physiological relevance, CAs have also attracted considerable interest in biotechnological applications. Their immobilization on nanostructured materials has enabled the development of nanobiocatalysts for CO_2_ capture and sequestration [16,17], while recent progress in zirconium–organic frameworks has yielded CA mimetics [18] featuring Zr–OH groups that emulate the architecture and function of the native catalytic centre. Because of their capacity to capture and transform environmental CO_2_, CAs have been postulated as promising tools for strategies aimed at mitigating global warming [19].

CAs are widespread across living organisms of all the phylogenetic tree and are currently grouped into eight evolutionarily distinct families [20,21], designated by the Greek letters α (present in vertebrates, plants, algae, and some bacteria), β (found in bacteria, plants, fungi, and algae), γ (bacteria and archaea), δ and ζ (mainly diatoms), η (Plasmodium species), θ (photosynthetic organisms), and ι (some bacteria and protozoa), the latter being the most recently identified [22].

Their catalytic mechanism relies on the presence of a metal ion located within the active site [23]. Although various divalent cations, including Fe^2+^, Co^2+^, and Cd^2+^, may be present, Zn^2+^ is by far the most common and physiologically relevant [24]. Interestingly, enzymes belonging to the ι-CA family appear to operate in the absence of a coordinated metal ion [25]. Kinetic studies revealed [26] that the metal cation acts as a Lewis acid, lowering the p*K*_a_ of the bound water molecule from 15.7 to approximately 7, thereby enabling hydroxide formation under physiological conditions. This metal-bound hydroxide then reacts with CO_2_ to generate HCO_3_^−^, which is subsequently replaced by a new water molecule; deprotonation of this incoming H_2_O molecule regenerates the active catalytic species and completes the catalytic cycle [20].

Human CAs (hCAs) belong to the α-family, with 15 isoforms currently identified [27]. These comprise cytosolic (hCA I, II, III, VII, and XIII), mitochondrial (VA and VB), transmembrane (hCA IX, XII, and XIV), membrane-anchored (hCA IV), and secreted enzymes (hCA VI), as well as CA-related proteins (CARP, hCA VIII, X, and XI) [28], the latter category devoid of known catalytic activity.

CAs are implicated in a wide range of diseases and pathological conditions. Consequently, the design of selective CA inhibitors and activators has become an active and highly relevant field in Medicinal Chemistry [27]. Therapeutic applications include glaucoma [29], epilepsy [30], obesity [13], cariogenesis [31], neurodegenerative disorders [32], cancer [33], and microbial infections [34], among others.

In oncology, the hypoxia-associated isoforms CA IX and XII are of particular interest, as they are overexpressed in a wide number of solid tumours [35]. CA IX contributes to the acidification of the tumour microenvironment, a phenomenon associated with enhanced tumour cell survival, invasiveness, and metastatic potential [36], and its overexpression correlates with poor prognosis [37]. Additionally, inhibition of CA XII may reduce the *P*-glycoprotein (P-gp)-mediated efflux of xenobiotics [38], potentially mitigating chemoresistance in cancer patients.

## 2. Results and Discussion

### 2.1. Drug Design and Chemistry

The main objective pursued in this manuscript was the design of coumarin derivatives with selective inhibitory activity against tumour-associated CA isoforms IX and XII, together with relevant antiproliferative properties. Approximately 45 chemical families are currently known to inhibit CAs through five different mechanisms [39]: (i) direct Zn^2+^ chelation; (ii) chelation of the Zn-bound H_2_O molecule; (iii) occlusion of the active site entrance; (iv) binding to the outer region; (v) halogen-bond formation mediated by His65.

Among all CA inhibitors, alkyl/aryl sulfonamides and coumarins represent the two most widely exploited chemotypes, although they act through markedly different mechanisms [39]. Sulfonamides inhibit CAs via coordination of the catalytic Zn^2+^ ion by the deprotonated sulfonamide nitrogen (class-i). By contrast, coumarins behave as suicide inhibitors: the intrinsic esterase activity of CAs hydrolyses their lactone ring, producing *Z*-*o*-hydroxycinnamic acid species that sterically block the entrance of the active site (class-iii) [39]. Many coumarins exhibit notable isoform selectivity, with reduced affinity for cytosolic isoforms, thereby limiting the likelihood of severe adverse effects.

Based on these considerations, the coumarin scaffold was selected as the key pharmacophore to target CA IX and CA XII. This core was functionalized with various substituents at positions C-3 and C-4 (alkyl, aryl, and halogen groups). Previous evidence suggests that alkyl residues at these positions may attenuate hepatotoxicity by slowing the metabolic formation of transient 3,4-epoxide intermediates [40].

To further enhance binding interactions, we designed hybrid molecules in which the coumarin core was connected to an aromatic thioureido fragment through flexible hydrocarbon linkers of various lengths. Thioureas, frequent motifs in synthetic bioactive compounds [41,42], and versatile precursors for additional functionalities [43], are well recognized for their ability to engage in strong hydrogen-bond interactions with enzyme active or allosteric sites [44], thereby reinforcing inhibitor affinity. The appended hydrophobic aromatic ring, bearing diverse substituents such as alkyl or halogen atoms in different positions, was expected to participate in complementary interactions with peripheral regions of the enzyme. Following the well-established tail approach for CA inhibitor design [20], strategic elongation of the inhibitor structure has been shown to improve isoform selectivity.

The results are presented following a structure–activity relationship (SAR) sequence, beginning with the design of the coumarin–thiourea hybrids, followed by their enzymatic inhibition profiles, selectivity toward tumour-associated CA IX and CA XII, and their antiproliferative activity in relevant cancer cell lines. This organization allows for a clear interpretation of how structural modifications influence biological outcomes.

**Figure 1:**
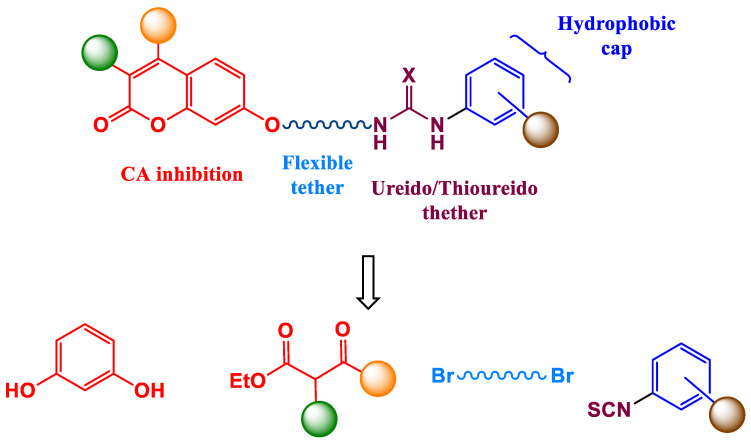
General structure of coumarin–thioureido hybrids.

**Scheme 1:**
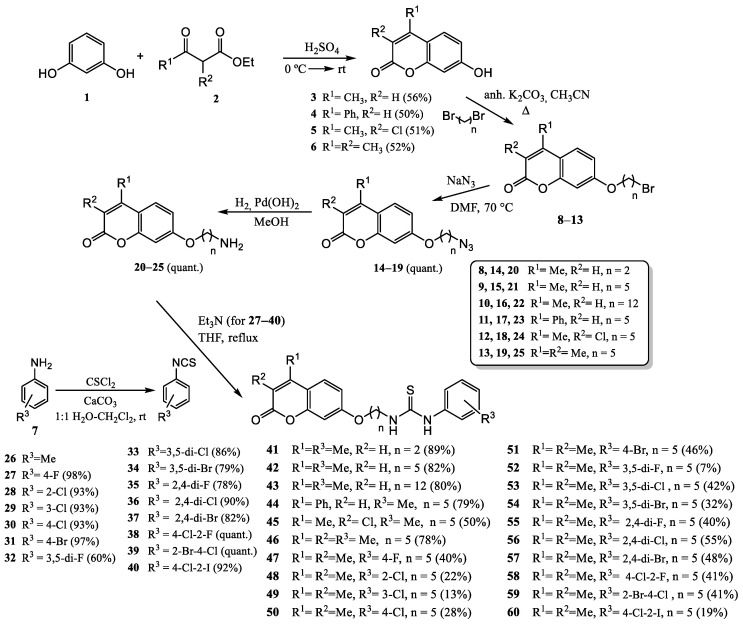
Preparation of thioureido-appended coumarins **41**–**60**.

Figure 1 displays the general structure of the compounds synthesized in this study. This structure shows the key scaffolds combined herein: the coumarin skeleton to afford CA inhibition, an aromatic residue acting as a hydrophobic cap to enhance such inhibition, a thioureido motif to promote hydrogen bonding interactions with the hydrophilic region of CA active site, and a flexible tether to afford conformational rotations when accommodating within the enzyme active site. The key chemical precursors to afford such hybrids include resorcinol (1,3-dihydroxybenzene) and substituted β-keto esters, used to construct the coumarin core, together with a series of aromatic isothiocyanates employed to introduce the thioureido unit. The synthetic strategy includes convergent synthesis, as described in Scheme 1.

The synthetic pathway is outlined in Scheme 1. Acid-catalyzed Pechmann condensations [45] between resorcinol (**1**) and the corresponding β-ketoesters **2** at low temperature afforded coumarins **3**–**6**, with different substitution patterns at the C-3/C-4 positions. These substituents were expected not only to limit the generation of potentially hazardous metabolites but also to influence interactions of the resulting molecules with the biological targets.

Subsequent Williamson synthesis under basic conditions (K_2_CO_3_) between the free phenolic group and an excess of diverse α,ω-dibromoalkanes provided intermediates **8**–**13**. Nucleophilic substitution of the terminal bromide with NaN_3_, followed by standard hydrogenolysis, furnished the ω-amino derivatives **20**–**25** in quantitative yields. Coupling of these amines with substituted aryl isothiocyanates **33**–**40** afforded the target thioureas **41**–**60** (Scheme 1). The aryl isothiocyanates were prepared in good to quantitative yields by reaction of the appropriate anilines with thiophosgene in a triphasic medium (H_2_O/CH_2_Cl_2_/CaCO_3_) [46]. Derivatives **41**–**46** had previously been employed by our group as intermediates in the preparation of coumarin-derived guanidines [47], although their biological properties had not been evaluated.

**Figure 2:**
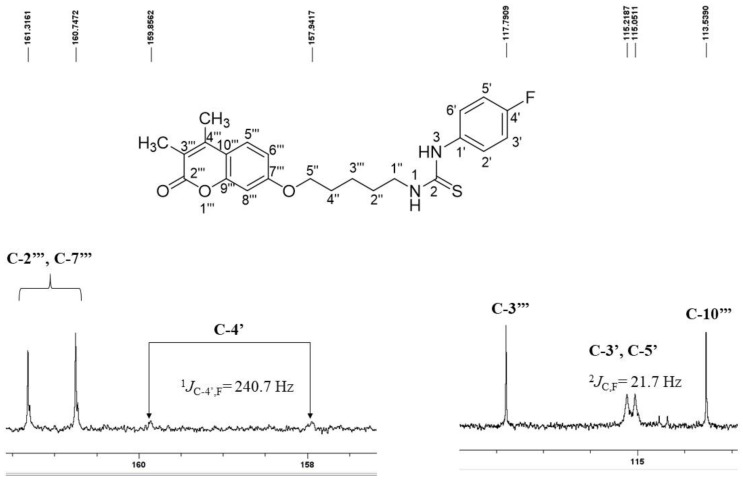
Partial ^13^C-NMR spectrum of thiourea **47**, showing C,F couplings.

The presence of the thioureido group in all final derivatives was confirmed by the characteristic ^13^C-NMR resonance at roughly 180 ppm, consistent with literature values [48]. Mono- and difluorinated derivatives additionally exhibited C-F couplings. Figure 2 exemplifies these observations for compound **47** in particular, for positions C-3/C-5 and C-4 of the aromatic residue.

Some additional minor peaks, showing chemical shifts similar to those reported in the Experimental Section, can be observed in certain ^13^C-NMR spectra. This phenomenon can be attributed to the presence of conformers [49], arising from the partial C=N double-bond character of the thioureido scaffold. A similar situation was recently described by some of us [50] for selenoureas derived from trihydroxypiperidine.

The library of compounds prepared herein encompassed mono- and disubstituted patterns at the C-3/C-4 positions of the coumarin core, linkers containing two to twelve carbon atoms, and mono- or disubstituted (alkyl or halogen) patterns on the thiourea-attached aromatic moiety. The biological evaluation—including inhibition of tumour-associated CA isoforms and in vitro antiproliferative assays (see subsequent sections)—revealed that the most favourable structural combination featured a dimethyl-substituted coumarin core linked through a pentyl chain. Guided by these results, we subsequently explored the influence of the phenyl ring in derivatives **47**–**60**.

Halogen atoms are frequently present in bioactive molecules [51]. In particular, fluorine is incorporated in a large number of marketed drugs due to its unique physicochemical properties. Halogen substituents allow for fine-tuning of molecular lipophilicity, improve metabolic stability by replacing labile C–H bonds, and can establish directional halogen bonds between C–X fragments and protein residues, thereby increasing the number of drug–target interactions [52]. Guided by these principles, we systematically varied the number, identity, and positional arrangement of halogens in the molecules described in this manuscript (Scheme 1), enabling a comprehensive structure–activity relationship (SAR) assessment.

### 2.2. Biological Assessments

Although stability assays were not accomplished in the present study, preliminary observations during synthesis, purification, and storage indicated that all coumarin–thiourea derivatives remained chemically stable at room temperature and under refrigerated conditions.

#### 2.2.1. CA Inhibition

Thioureas **41**–**60** were evaluated as potential inhibitors of the tumour-associated CA isoforms IX and XII. The cytosolic isoforms CA I and II were also examined to assess selectivity, and AAZ was included as the reference drug. Inhibition constants (*K*_i_, nM), determined using the stopped-flow CO_2_ hydrase assay, are summarized in Table 1, and the corresponding selectivity indexes (S.I.s) are shown in Table 2. Several structure–activity relationships were identified and are discussed below:

- **Influence of the linker length:** The effect of the alkyl spacer was examined by comparing monomethylated thioureas **41** (n = 2), **42** (n = 5) and **43** (n = 12). The pentyl linker provided the most favourable architecture, affording low nanomolar inhibition of CA IX and CA XII (*K*_i_ = 6.7 and 9.8 nM, respectively). Both shorter and longer linkers resulted in approximately a ten-fold loss of potency for the two isoforms.
- **Ureido vs. thioureido connector:** Pentyl ureido derivative **61** was previously obtained serendipitously [47] during an attempted H_2_O_2_/TBAI-mediated cyclodesulfurization of thiourea **42** to the corresponding guanidine. Comparison between **42** and **61** revealed that the ureido group preserved strong inhibition of CA IX but caused a 4.5-fold decrease in potency against CA XII.
- **Effect of mono and disubstitution on the coumarin core:** Using the pentyl chain as the optimal linker, the impact of C-3 and C-4 substitution was examined (**42** vs. **44**–**46**). Introducing a bulky phenyl group at C4 caused a major loss of activity (112-fold for CA IX and 30-fold for CA XII). In contrast, disubstituted analogues (Me/Cl or Me/Me) maintained strong CA IX inhibition. These results indicate that steric hindrance at C4 is strongly detrimental.
- **Selectivity:** Although none of these compounds surpassed AAZ in absolute potency, their selectivity indexes (S.I.s) were far superior. Compounds **42** and **46** displayed S.I. values of 10,204.1–14,925.4 and 2004.0–16,666.7, respectively, compared to 0.48–43.8 for AAZ. This highlights their significantly reduced inhibition of cytosolic isoforms and thus a lower risk of off-target effects.
- **Selection of scaffold for further optimization:** Although **42** was the most potent inhibitor of both isoforms in the **41**–**46** series, compound **46** was chosen for further derivatization (**47**–**60**) because of its superior antiproliferative properties, which are discussed in the next section.
- **Alkyl vs. halogen substituents on the aromatic ring:** Replacing the *p*-methyl group of **42** with a halogen (**47**: *p*-F; **50**: *p*-Cl; **51**: *p*-Br) reduced potency against CA IX and XII and caused a marked loss of selectivity. This indicates that para-electron-withdrawing groups negatively affect thiourea binding to tumour-associated CA isoforms.
- **Influence of halogen position:** In the chlorine series (**48**–**50**), the *meta*-substituted analogue **49** showed better selectivity despite being slightly less potent than the *para*-substituted **50**. The *ortho*-substituted analogue **48** was the least potent and selective, likely due to steric hindrance and unfavourable binding orientation.
- **Addition of a second identical halogen atom:** Introducing a second identical halogen at the *ortho* position (2,4-disubstituted compounds **55**–**57**) generally increased selectivity relative to their monosubstituted counterparts (**47**, **50**, **51**). Conversely, the 3,5-disubstituted analogues (**52**–**54**) showed lower S.I. values, indicating that the 2,4-pattern better promotes isoform discrimination.
- **Disubstitution with different halogens:** In derivatives bearing two different halogens at C2 and C4 (**58**–**60**), incorporation of an iodine atom at C2 substantially improved CA IX potency and selectivity compared with the 2,4-dichloro analogue **56** (*K*_i_: 13.3 nM vs. 67.8 nM).

**Table 1:** Inhibition constants (*K*_i_, nM) of compounds **41**–**61** against hCAs I, II, IX, and XII ^a^.

Compound	hCA I	hCA II	hCA IX	hCA XII
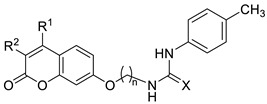				
**41** (n = 2, R^1^ = Me, R^2^ = H, X = S)	>10,000	>10,000	87.6	80.6
**42** (n = 5, R^1^ = Me, R^2^ = H, X = S)	>100,000	>100,000	6.7	9.8
**61** (n = 5, R^1^ = Me, R^2^ = H, X = O)	>100,000	>100,000	6.6	44.2
**43** (n = 12, R^1^ = Me, R^2^ = H, X = S)	>10,000	>10,000	78.7	89.5
**44** (n = 5, R^1^ = Ph, R^2^ = H, X = S)	>10,000	>10,000	751	291
**45** (n = 5, R^1^ = Me, R^2^ = Cl, X = S)	>10,000	>10,000	6.7	20.2
**46** (n = 5, R^1^ = R^2^ = Me)	>100,000	>100,000	6.0	49.9
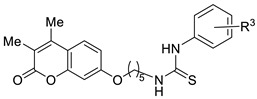				
**47** (R^3^ = 4-F)	7772	1125	48.2	40.9
**48** (R^3^ = 2-Cl)	10,100	3201	104.7	53.6
**49** (R^3^ = 3-Cl)	26,900	18,600	60.6	33.4
**50** (R^3^ = 4-Cl)	17,000	12,200	57.8	46.2
**51** (R^3^ = 4-Br)	35,400	16,500	167.0	26.2
**52** (R^3^ = 3,5-di-F)	7908	2585	18.8	15.7
**53** (R^3^ = 3,5-di-Cl)	58,700	20,700	440.6	116.9
**54** (R^3^ = 3,5-di-Br)	45,900	9220	38.6	29.8
**55** (R^3^ = 2,4-di-F)	73,800	11,500	76.5	91.8
**56** (R^3^ = 2,4-di-Cl)	48,900	14,600	67.8	83.2
**57** (R^3^ = 2,4-di-Br)	>100,000	68,600	14.4	184.6
**58** (R^3^ = 4-Cl-2-F)	67,000	18,200	93.4	311.0
**59** (R^3^ = 2-Br-4-Cl)	39,500	27,700	196.0	61.2
**60** (R^3^ = 4-Cl-2-I)	>100,000	45,700	13.3	91.3
**AAZ**	250.0	12.0	25.0	5.7

^a^ Mean from three different assays by a stopped flow technique (errors were in the range of ±5–10% of the reported values).

**Table 2:** Selectivity indexes (S.I.s) for the inhibition of human CAs.

Compound	S.I. I/IX	S.I. I/XII	S.I. II/IX	S.I. II/XII
**41** (n = 2, R^1^ = Me, R^2^ = H, X = S)	>114.2	>124.1	>114.2	>124.1
**42** (n = 5, R^1^ = Me, R^2^ = H X = S)	>14,925.4	>10,204.1	>14,925.4	>10,204.1
**61** (n = 5, R^1^ = Me, R^2^ = H X = O)	>15,151.5	>2262.4	>15,151.5	>2262.4
**43** (n = 12, R^1^ = Me, R^2^ = H, X = S)	>127.1	>111.7	>127.1	>111.7
**44** (n = 5, R^1^ = Ph, R^2^ = H, X = S)	>13.3	>34.4	>13.3	>34.4
**45** (n = 5, R^1^ = Me, R^2^ = Cl, X = S)	>1492.5	>495.0	>1492.5	>495.0
**46** (n = 5, R^1^ = R^2^ = Me)	>16,666.7	>2004.0	>16,666.7	>2004.0
**47** (R^3^ = 4-F)	161.2	190.0	23.3	27.5
**48** (R^3^ = 2-Cl)	96.5	188.4	30.6	59.7
**49** (R^3^ = 3-Cl)	443.9	805.4	306.9	556.9
**50** (R^3^ = 4-Cl)	294.1	367.9	211.1	264.1
**51** (R^3^ = 4-Br)	211.9	1351.1	98.87	629.7
**52** (R^3^ = 3,5-di-F)	420.6	503.7	137.5	164.6
**53** (R^3^ = 3,5-di-Cl)	133.2	502.1	46.9	177.1
**54** (R^3^ = 3,5-di-Br)	1189.1	1540.3	238.9	309.4
**55** (R^3^ = 2,4-di-F)	964.7	803.9	150.3	125.3
**56** (R^3^ = 2,4-di-Cl)	721.2	587.7	215.3	175.5
**57** (R^3^ = 2,4-di-Br)	>6944.4	>541.7	4763.8	371.6
**58** (R^3^ = 4-Cl-2-F)	717.3	215.4	194.9	58.5
**59** (R^3^ = 2-Br-4-Cl)	201.5	645.4	141.3	452.6
**60** (R^3^ = 4-Cl-2-I)	>7518.8	>1095.3	3436.1	500.5
**AAZ**	10.0	43.8	0.48	2.1

The selectivity of the studied compounds toward tumour-associated CA IX and XII is encouraging (Table 2) and considerably higher compared to the reference compound, AAZ. The exceptional good selectivity ratio towards CAs IX and XII relative to off-target enzymes (CAs I and II) highlight the potential use of these coumarin–thiourea hybrids in cancer research. Inhibition of non-tumour isoforms such as cytosolic CA I and CA II may lead to off-target physiological effects, including metabolic acidosis, gastrointestinal irritation, depression, and fatigue, among others [53]. Although the inhibition of CA I and CA II was significantly weaker for most derivatives, particularly the lead compounds **45** and **46**, some residual affinity cannot be excluded. A comprehensive assessment of off-target inhibition profiles and functional effects in normal tissues will therefore be required in future studies to ensure a favourable safety margin.

Among the synthesized series, compounds **45** and **46** displayed the most favourable biological profile and therefore were further studied in detail.

Recently, coumarin-derived ureas and amides bearing substituents at the C-6 position of the coumarin scaffold were reported [54] as the products of coupling 6-aminocoumarin with aryl isocyanates or substituted benzoic acids. However, these compounds lack a tether linking the pharmacophoric regions. Thioureas **45** and **46**, which share a similar selectivity and potency profile toward CA XII, displayed an approximately ten-fold increase in inhibitory activity against CA IX. Moreover, because these reported derivatives were obtained from unsubstituted 6-aminocoumarin, they are likely to undergo faster metabolic degradation [40].

A comparable scenario emerged when comparing **45** and **46** with thiourea derivatives obtained from 6-aminocoumarin [55], which showed markedly reduced inhibition of both CA IX and CA XII (up to 60-fold) relative to their C-7-substituted counterparts. Altogether, these findings underscore the critical role of C-7 substitution and the presence of a flexible tether connecting the coumarin and thioureido moieties for achieving potent and selective CA inhibition.

#### 2.2.2. Docking Simulations

Compound **46** was subjected to molecular modelling simulations against CAIX and CAXII. Since the coumarin moiety has been reported [39] to be hydrolyzed because of the CA esterase activity to give a cinnamic acid derivative, the open form—with the corresponding *Z*-configured *o*-hydroxycinnamic acid instead of the closed coumarin—of **46** was used. Binding energies are represented in Table 3.

**Figure 3:**
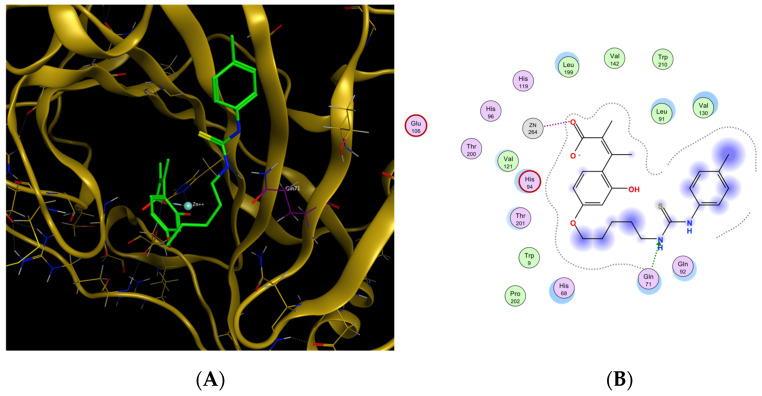
(**A**) Three-dimensional representation of CAIX (gold)–**46** (green) binding complex. Gln 71 is depicted in purple. (**B**) Two-dimensional scheme of the protein–ligand interactions.

The cinnamic acid residue was predicted to be oriented towards the catalytic site of the pocket in both of the CA isoforms, while the thiourea was projected towards the outside. In CAIX, the carboxylate group of the hydroxycinnamic moiety interacts directly with the Zn^2+^ cation of the catalytic site, whilst the thiourea allows the molecule to interact with Gln71 (Figure 3).

**Figure 4:**
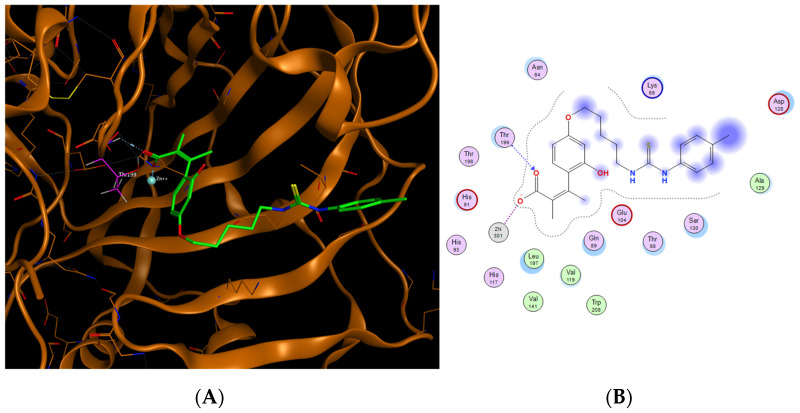
(**A**) Three-dimensional representation of CAXII (orange)–**46** (green) binding complex. Thr 199 is depicted in purple. (**B**) Two-dimensional scheme of the protein–ligand interactions.

The strongest binding energy predicted in the **46**-CAXII complex is explained by a simultaneous interaction with the Zn^2+^ cation and Thr199 through the carboxylate moiety (Figure 4).

#### 2.2.3. In Vitro Antiproliferative Properties

Antiproliferative properties of thioureas **41**–**60** were evaluated following the protocol of the US National Cancer Institute, using the panel of human solid tumour cell lines depicted in Table 4: A549 (non-small cell lung), HBL-100 (breast), HeLa (cervix), SW1537 (non-small cell lung), and the multidrug-resistant cell lines T-47D (breast) and WiDr (colon). Coumarin precursors **3**–**6** and drugs 5-fluorouracyl (5-FU) and cisplatin were also incorporated for comparison. Activities are expressed as GI_50_ values (Growth Inhibition at 50%, µM).

The following conclusions can be extracted:

- **Coumarin precursors:** The parent coumarins **3**–**6** displayed weak or negligible antiproliferative activity, with the only exception being compound **3** in HeLa cells (GI_50_ = 9.2 µM).
- **Effect of thiourea introduction and spacer length (monomethylated compounds 41–43):** Incorporation of an *N*-aryl thiourea dramatically enhanced antiproliferative activity, by more than 26-fold in some cases, highlighting the essential role of the thioureido group, likely through enhanced H-bonding and/or metal-binding interactions.
- **Influence of coumarin substitution (44–46):** With the tether fixed at n = 5, steric and electronic effects of substituents at C-3/C-4 were examined. The bulky phenyl group in **44** caused a complete loss of activity, confirming the negative impact of steric hindrance. In contrast, disubstituted coumarins **45** and **46** were highly potent, showing GI_50_ values in the low micromolar range (**45**: 2.2–4.2 µM; **46**: 1.9–3.5 µM). The activity trend Ph/H ≪ Me/Cl ≈ Me/Me indicates that substituents with reduced steric demand and moderate electron-withdrawing character are optimal for potency.
- **Thiourea vs. urea isosterism:** Replacing the thiourea in **42** with a urea moiety (**61**) abolished antiproliferative activity (GI_50_ > 100 µM), demonstrating that the thiocarbonyl group is essential, likely due to its higher NH acidity, superior H-bond donor capacity, and potential metal-coordination ability.
- **Effects of aryl halogenation (47–60):** After identifying the optimal coumarin core (R_1_ = R_2_ = Me) and linker length (n = 5), the effect of halogenation of the terminal aryl ring was explored. Monohalogenated *para*-derivatives (**47**, **50**, and **51**) exhibited moderate to low activity, whereas their *ortho*- and *meta*-substituted analogues were even less potent. Within the 3,5-dihalogenated series (**52**–**54**), a clear trend emerged, di-F (inactive) < di-Cl (26–33 µM) < di-Br (13–17 µM), consistent with the increasing size and polarizability of the halogens, which can enhance hydrophobic contacts and potentially halogen bonding.
  In multidrug-resistant cell lines, a general loss of activity was observed.
  The 2,4-dihalogenated compounds showed moderate activity overall.
  Among the mixed halogenated derivatives (**58**–**60**), activity improved slightly compared with the 2,4-dichloro analogue **56**. Notably, compound **60** showed a pronounced activity increase in HeLa cells (GI_50_ = 7.6 µM).
- **Comparison with 5-FU and cisplatin:** Lead thioureas (**45** and **46**) displayed GI_50_ values similar to those of reference drugs (5-FU and cisplatin) in sensitive cell lines and surpassed them in multidrug-resistant models, achieving up to a 15-fold improvement in potency.

These data clearly demonstrate that coupling a coumarin scaffold with an appended *N*-aryl thiourea at the C-7 position provides a highly effective framework for the development of antiproliferative compounds. Biological activity was strongly influenced by C-3/C-4 substitution on the coumarin ring, by the nature and position of halogens on the terminal aryl group, and by the tether length connecting both pharmacophore domains. Compounds **45** and **46** emerge as the lead structures, balancing electronic, steric, and lipophilic factors to yield potencies comparable to, and in some cases exceeding, those of cisplatin in multidrug-resistant cell lines.

**Table 4:** Antiproliferative activity of **3**–**6** and **41**–**61** (GI_50_, µM).

Compound	A549 (Non-Small Cell Lung)	HBL-100 (Breast)	HeLa (Cervix)	SW1573 (Non-Small Cell Lung)	T-47D (Breast)	WiDr (Colon)
	Sensitive Lines				Multidrug-Resistant Lines	
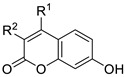						
**3** (R^1^ = Me, R^2^ = H)	>100		9.2 ± 0.7	>100		
**4** (R^1^ = Ph, R^2^ = H)	22 ± 9	39 ± 12	19 ± 7	22 ± 3	86 ± 20	35 ± 12
**5** (R^1^ = Me, R^2^ = Cl)	47 ± 2	>100	50 ± 5	88 ± 17	>100	
**6** (R^1^ = R^2^ = Me)	68 ± 0	>100				
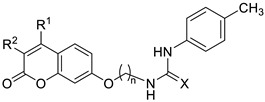						
**41** (n = 2, R^1^ = Me, R^2^ = H, X = S)	3.8 ± 0.1	22 ± 9	17 ± 1	8.6 ± 4.0	12 ± 4	9.7 ± 2.0
**42** (n = 5, R^1^ = Me, R^2^ = H, X = S)	23 ± 7	69 ± 4	30 ± 2	39 ± 3	78 ± 6	62 ± 8
**61** (n = 5, R^1^ = Me, R^2^ = H, X = O)	>100					
**43** (n = 12, R^1^ = Me, R^2^ = H, X = S)	4.9 ± 1.0	5.4 ± 1.0	4.0 ± 0.7	4.6 ± 1.0	8.4 ± 2.0	5.4 ± 0.4
**44** (n = 5, R^1^ = Ph, R^2^ = H, X = S)	>100					
**45** (n = 5, R^1^ = Me, R^2^ = Cl, X = S)	2.2 ± 0.9	4.2 ± 0.6	3.1 ± 0.8	3.1 ± 0.9	3.5 ± 0.6	3.3 ± 0.6
**46** (n = 5, R^1^ = R^2^ = Me)	1.9 ± 0.2	3.5 ± 0.9	2.8 ± 0.3	2.7 ± 0.5	3.1 ± 0.2	3.2 ± 0.6
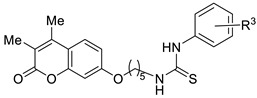						
**47** (R^3^ = 4-F)	>100				89 ± 16	71 ± 6
**48** (R^3^ = 2-Cl)	95 ± 7	>100			85 ± 21	67 ± 5
**49** (R^3^ = 3-Cl)	>100		98 ± 3	92 ± 8	>100	
**50** (R^3^ = 4-Cl)	62 ± 14	31 ± 8	40 ± 5	26 ± 6	40 ± 7	88 ± 17
**51** (R^3^ = 4-Br)	39 ± 10	31 ± 5	23 ± 6	>100	87 ± 18	>100
**52** (R^3^ = 3,5-di-F)	>100					
**53** (R^3^ = 3,5-di-Cl)	33 ± 4	26 ± 10	33 ± 10	26 ± 4	77 ± 8	>100
**54** (R^3^ = 3,5-di-Br)	15 ± 2	17 ± 3	16 ± 2	13 ± 2	37 ± 1	44 ± 4
**55** (R^3^ = 2,4-di-F)	72 ± 0	59 ± 7	91 ± 13	>100	81 ± 5	95 ± 7
**56** (R^3^ = 2,4-di-Cl)	53 ± 4	51 ± 6	32 ± 4	>100	71 ± 6	>100
**57** (R^3^ = 2,4-di-Br)	42 ± 2	45 ± 0	69 ± 3	>100	73 ± 21	59 ± 3
**58** (R^3^ = 4-Cl-2-F)	16 ± 2	21 ± 6	17 ± 2	63 ± 21	33 ± 2	48 ± 21
**59** (R^3^ = 2-Br-4-Cl)	23 ± 3	28 ± 10	36 ± 0	>100	63 ± 13	33 ± 3
**60** (R^3^ = 4-Cl-2-I)	30 ± 0	30 ± 3	7.6 ± 4.5	>100	54 ± 30	34 ± 4
**5-FU**	2.2 ± 0.3	4.4 ± 0.7	16 ± 5	3.3 ± 1.2	43 ± 16	49 ± 7
**Cisplatin**	4.9 ± 0.2	1.9 ± 0.2	1.8 ± 0.5	2.7 ± 0.4	17 ± 3	26 ± 4

**Figure 5:**
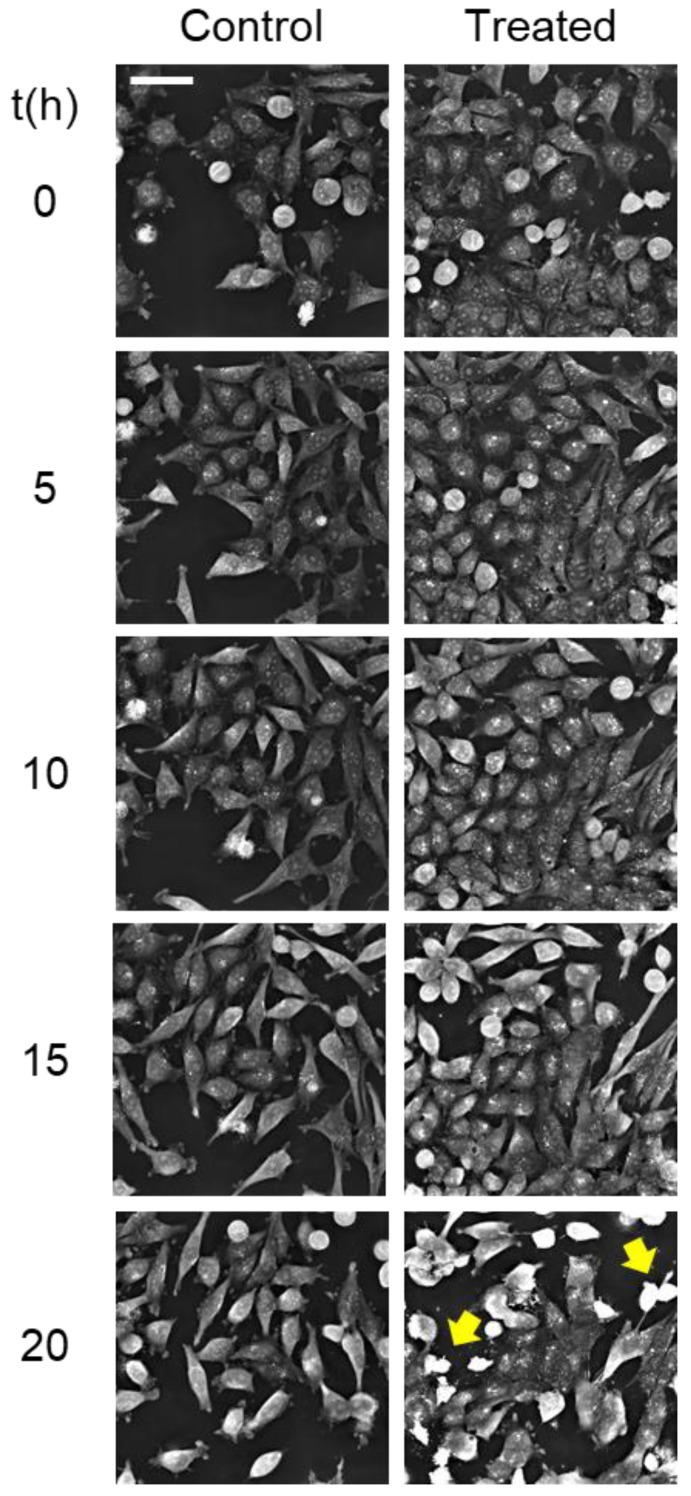
Effects observed in HeLa cells exposed to compound **46**. Snapshots at different time intervals of the continuous live-cell monitoring for 20 h of untreated (left panel) and treated (right panel, 10 μM) cells. Yellow arrows point to cells with morphological apoptotic features. Scale bar: 20 µm.

We selected compound **46** for a preliminary characterization of its mode of action. Thus, we monitored HeLa in real time using label-free holotomographic 3D microscopy. Cells were exposed for 20 h to compound **46** at 40 µM (based on the GI_50_ values against HeLa cells) and the cultures were monitored every 10 min for 20 h (Supplementary Videos S1 and S2). Figure 5 shows that compound 46 induced cell damage after approximately 10 h of exposure, when compared to untreated cells. The appearance of blebs indicates that the compounds trigger apoptosis in HeLa cells. These perturbations of the membrane were not observed in the control group. After exposure, cells started to shrink, decreasing their volume and forming apoptotic bodies, with cells from the treated group with **46** being dead by the end of the experiment (20 h).

## 3. Materials and Methods

### 3.1. Chemistry

#### 3.1.1. General Methods

TLC (Thin-Layer Chromatography) was performed using aluminum-coated sheets (Merck 60 F_254_, Darmstadt, Germany) of 0.25 mm width. Each eluant is indicated in the experimental procedures. Spots were visualized by UV light (λ = 254 nm) and by charring with 10% ethanolic vanillin containing 1% H_2_SO_4_ or with 5% ethanolic phosphomolybdic acid. Column chromatography purifications were performed using silica gel stationary phase (Merck 60, particle size 40–63 μm), eluting by gravity or with mild pressure. Eluants are indicated in each case. NMR spectra were registered in the Centro de Investigación, Tecnología e Innovación de la Universidad de Sevilla (CITIUS), using Bruker Avance III 300 and 500 spectrometers (300.1 MHz and 500 MHz for ^1^H; 75.5 and 125.7 MHz, respectively, for ^13^C) (Billerica, MA, USA), and DMSO-*d*_6_ as a solvent (VWR Chemicals, Radnor, PA, USA). Assignments were confirmed with 2D homo- and heteronuclear experiments (COSY, HSQC). Chemical shifts (δ) are expressed in ppm, and coupling constants (*J*) are expressed in Hz. Residual signals from the solvent are used as internal references [56] for the calibration (2.50 ppm for ^1^H and 39.5 ppm for ^13^C). Mass spectra were registered using a Qexactive spectrometer (Thermo Scientific, Waltham, MA, USA), using Electrospray Ionization (ESI). Sample injection (MeOH as solvent) was performed using UHPLC without a column (50 μL and acquisition for 3 min at 0.200 mL/min). Acquisition was carried out with a full scan at a 60,000 resolution. The capillary temperature was 350 °C and the source voltage was 3.5 kV. Spectra were registered under positive mode and calibrated using Pierce™ LTQ Velos ESI Positive Ion Calibration Solution (ThermoFisher Scientific, Waltham, MA, USA).

#### 3.1.2. General Procedure for the Preparation of Thioureas **41**–**60**

The corresponding isothiocyanate **26**–**40** (1.5–3.0 equiv.) and Et_3_N (1.0 equiv., for isothiocyanates **27**–**40**) was added to a solution of amines **20**–**25** (1.0 eq.) in THF (10 mL). The mixture was refluxed for the time indicated in each case. After that, the crude reaction was concentrated to dryness and purified by column chromatography (cyclohexane–EtOAc mixtures, for isothiocyanate **26**), or washed with Et_2_O (isothiocyanates **27**–**40**).

Thioureas **41**–**46** were reported previously [47]. ^1^H-, ^13^C-NMR and HR-ESIMS spectra of compounds **47**–**60** are displayed in Figures S1–S42 (Supplementary Materials).

1-{5″-[(3’’’,4’’’-Dimethyl-2’’’-oxo-2’’’*H*-chromen-7’’’-yl)oxy]pentyl}-3-(4′-fluorophenyl)thiourea (**47**): Amine **25** (84.7 mg, 0.31 mmol, 1.0 equiv.), isothiocyanate **27** (142.5 mg, 0.93 mmol, 3.0 equiv.) and Et_3_N (43 µL, 0.31 mmol, 1.0 equiv.) were used, and the reaction was refluxed for 15 h. Compound **47** was obtained as a brown solid. Yield: 52.7 mg (40%). Mp: 100–110 °C; ^1^H-NMR (500 MHz, DMSO-*d*_6_) δ 9.53 (brs, 1H, Ar-NH), 7.81 (brs, 1H, CH_2_-NH), 7.67 (d, 1H, *J*_5’’’,6’’’_ = 9.6 Hz, H-5’’’), 7.39 (m, 2H, H-3′, H-5′), 7.13 (m, 2H, H-2′, H-6′), 6.93 (m, 2H, H-6’’’,H-8’’’), 4.07 (t, 2H, *J*_H,H_ = 6.5 Hz, OCH_2_), 3.47 (brs, 2H, CH_2_N), 2.35 (s, 3H, CH_3_), 2.06 (s, 3H, CH_3_), 1.76 (quint, 2H, *J*_H,H_ = 6.4 Hz, CH_2_), 1.60 (quint, 2H, *J*_H,H_ = 7.2 Hz, CH_2_), 1.45 (quint, 2H, *J*_H,H_ = 7.0 Hz, CH_2_) ppm; ^13^C-NMR (125.7 MHz, DMSO-*d*_6_) δ 180.7 (C=S), 161.3, 160.7 (C-2’’’, C-7’’’), 158.9 (d, ^1^*J*_C_._F_ = 240.7 Hz, C-4′), 153.1 (C-9’’’), 146.9 (C-4’’’), 135.7 (brs, C-1′), 126.1 (C-5’’’), 125.5 (brs, C-2′, C-6′), 117.8 (C-3’’’), 115.1 (d, ^2^*J*_C,F_ = 21.7 Hz, C-3′, C-5′), 113.5 (C-10’’’), 112.3 (C-6’’’), 100.9 (C-8’’’), 68.1 (OCH_3_), 43.7 (NCH_3_), 28.2 (x2), 22.9 (CH_2_), 14.9 (CH_3_), 12.9 (CH_3_) ppm; HR-ESIMS *m*/*z* calcd. for C_23_H_25_FN_2_NaO_3_S ([M + Na]^+^): 451.1462, found: 451.1456.

1-(2′-Chlorophenyl)-3-{5″-[(3’’’,4’’’-dimethyl-2’’’-oxo-2’’’*H*-chromen-7’’’-yl)oxy]pentyl}thiourea (**48**): Amine **25** (96.4 mg, 0.35 mmol, 1.0 equiv.), isothiocyanate **28** (178.1 mg, 1.05 mmol, 3.0 equiv.) and Et_3_N (49 µL, 0.35 mmol, 1.0 equiv.) were used, and the reaction was refluxed for 12 h. Compound **48** was obtained as a brown solid. Yield: 34.0 mg (22%). Mp: 90–95 °C; ^1^H-NMR (300 MHz, DMSO-*d*_6_) δ 9.12 (brs, 1H, Ar-NH), 8.02 (brs, 1H, CH_2_-N*H*), 7.68 (m, 2H, H-5’’’, H-3′), 7.46 (dd, 1H, *J*_4′,6′_ = 1.4 Hz, *J*_5′,6′_ = 7.9 Hz, H-6′), 7.30 (td, 1H, *J*_H,H_ = 1.4 Hz, *J*_H,H_ = 7.5 Hz, H-4′ or H-5′), 7.21 (brt, 1H, *J*_H,H_ = 7.2 Hz, H-4′ or H-5′), 6.94 (m, 2H, H-6’’’, H-8’’’), 4.08 (t, 2H, *J*_H,H_ = 6.0 Hz, OCH_2_), 3.48 (brs, 2H, CH_2_N), 2.36 (s, 3H, CH_3_), 2.07 (s, 3H, CH_3_), 1.76 (m, 4H, 2CH_2_), 1.63 (quint, 2H, *J*_H,H_ = 6.0 Hz, CH_2_), 1.46 (quint, 2H, *J*_H,H_ = 6.0 Hz, CH_2_) ppm; ^13^C-NMR (125.7 MHz, DMSO-*d*_6_) δ 181.7 (C=S), 161.8, 161.2 (C-2’’’, C-7’’’), 153.5 (C-9’’’), 147.3 (C-4’’’), 136.6 (C-1′), 129.4 (Ar-C), 127.5 (C-5′), 126.6 (C-5’’’,Ar-C), 118.2 (C-3’’’), 114.0 (C-10’’’), 112.8 (C-6’’’), 100.8 (C-8’’’), 68.1 (OCH_3_), 45.5 (NCH_3_), 28.7, 28.1, 22.9 (CH_2_), 14.9 (CH_3_), 12.9 (CH_3_) ppm; HR-ESIMS *m*/*z* calcd. for C_23_H_25_ClN_2_NaO_3_NaS ([M + Na]^+^): 467.1167, found: 467.1158.

1-(3′-Chlorophenyl)-3-{5″-[(3’’’,4’’’-dimethyl-2’’’-oxo-2’’’*H*-chromen-7’’’-yl)oxy]pentyl}thiourea (**49**): Amine **25** (253.3 mg, 0.92 mmol, 1.0 equiv.), isothiocyanate **29** (468.2 mg, 2.76 mmol, 3.0 equiv.) and Et_3_N (129 µL, 0.92 mmol, 1.0 equiv.) were used, and the reaction was refluxed for 7 h. Compound **49** was obtained as a brown solid. Yield: 53.0 mg (13%). Mp: 105–110 °C; ^1^H-NMR (500 MHz, DMSO-*d*_6_) δ 9.66 (brs, 1H, Ar-NH), 7.99 (brt, 1H, *J*_NH,H_ = 5.1 Hz, CH_2_-N*H*), 7.75 (brt, 1H, *J*_2′,3′_ = *J*_2′,4′_ = 1.9 Hz, H-2′), 7.66 (d, 1H, *J*_5′’’,6′’’_ = 8.8 Hz, H-5’’’), 7.37 (ddd, 1H, *J*_H,H_ = 1.0 Hz, *J*_H,H_ = 2.0 Hz, *J*_H,H_ = 8.1 Hz, H-4′ or H-6′), 7.30 (t, 1H, *J*_4′,5′_ = *J*_5′,6′_ = 8.0 Hz, H-5′), 7.10 (ddd, 1H, *J*_H,H_ = 1.0 Hz, *J*_H,H_ = 2.2 Hz, *J*_H,H_ = 7.9 Hz, H-4′ or H-6′), 6.94 (dd, 1H, *J*_6’’’,8’’’_ = 2.6 Hz, H-6’’’), 6.91 (d, 1H, H-8’’’), 4.09 (t, 2H, *J*_H,H_ = 6.0 Hz, OCH_2_), 3.51 (q, 2H, *J*_NH,H_ = *J*_HH_ = 5.1 Hz, CH_2_N), 2.36 (s, 3H, CH_3_), 2.08 (s, 3H, CH_3_), 1.79 (quint, 2H, *J*_H,H_ = 6.0 Hz, CH_2_), 1.64 (quint, 2H, *J*_H,H_ = 6.0 Hz, CH_2_), 1.50 (quint, 2H, *J*_H,H_ = 6.0 Hz, CH_2_) ppm; ^13^C-NMR (125.7 MHz, DMSO-*d*_6_) δ 180.2 (C=S), 161.2, 160.6 (C-2’’’, C-7’’’), 153.0 (C-9’’’), 146.8 (C-4’’’), 141.2 (C-1′), 132.6 (C-5’’’), 130.1, 126.1, 123.2, 121.7, 120.7 (Ar-C), 117.8 (C-3’’’), 113.5 (C-10’’’), 112.3 (C-6’’’), 100.9 (C-8’’’), 68.1 (OCH_3_), 43.6 (NCH_3_), 28.2, 26.7, 22.9 (CH_2_), 14.9 (CH_3_), 12.9 (CH_3_) ppm; HR-ESIMS *m*/*z* calcd. for C_23_H_25_ClN_2_NaO_3_S ([M + Na]^+^): 467.1167, found: 467.1155.

1-(4′-Chlorophenyl)-3-{5″-[(3’’’,4’’’-dimethyl-2’’’-oxo-2’’’*H*-chromen-7’’’-yl)oxy]pentyl}thiourea (**50**): Amine **25** (63.3 mg, 0.23 mmol, 1.0 equiv.), isothiocyanate **30** (117.0 mg, 0.69 mmol, 3.0 equiv.) and Et_3_N (32 µL, 0.23 mmol, 1.0 equiv.) were used, and the reaction was refluxed for 7 h. Compound **50** was obtained as a brown solid. Yield: 29.0 mg (28%). ^1^H-NMR (300 MHz, DMSO-*d*_6_) δ 9.56 (brs, 1H, Ar-NH), 7.90 (brs, 1H, CH_2_-NH), 7.67 (m, 1H, H-5’’’), 7.45 (m, 2H, H-3′, H-5′), 7.33 (d, 2H, *J*_H,H_ = 9.0 Hz, H-2′, H-6′), 6.93 (m, 2H, H-6’’’, H-8’’’), 4.08 (t, 2H, *J*_H,H_ = 6.0 Hz, OCH_2_), 3.49 (brs, 2H, CH_2_N), 2.36 (s, 3H, CH_3_), 2.07 (s, 3H, CH_3_), 1.77 (quint, 2H, *J*_H,H_ = 9.0 Hz, CH_2_), 1.61 (quint, 2H, *J*_H,H_ = 9.0 Hz, CH_2_), 1.45 (quint, 2H, *J*_H,H_ = 6.0 Hz, CH_2_) ppm; ^13^C-NMR (125.7 MHz, DMSO-*d*_6_) δ 180.4 (C=S), 161.3, 160.7 (C-2’’’-C-7’’’), 153.0 (C-9’’’), 146.9 (C-4’’’), 138.5 (C-1′), 128.3 (C-4′), 126.1 (C-5’’’), 124.3 (C-2′,C-6′), 117.7 (C-3’’’), 113.5 (C-10’’’), 112.3 (C-6’’’), 100.8 (C-8’’’), 68.1(OCH_3_), 43.7 (NCH_3_), 28.2, 26.7, 22.9 (CH_2_), 14.9 (CH_3_), 12.9 (CH_3_) ppm; HR-ESIMS *m*/*z* calcd. for C_23_H_25_ClN_2_NaO_3_S ([M + H]^+^): 467.1167, found: 467.1159.

1-(4′-Bromophenyl)-3-{5″-[(3’’’,4’’’-dimethyl-2’’’-oxo-2’’’*H*-chromen-7’’’-yl)oxy]pentyl}thiourea (**51**): Amine **25** (57.8 mg, 0.21 mmol, 1.0 equiv.), isothiocyanate **31** (134.9 mg, 0.63 mmol, 3.0 equiv.) and Et_3_N (29 µL, 0.21 mmol, 1.0 equiv.) were used, and the reaction was refluxed for 15 h. Compound **51** was obtained as a yellow solid. Yield: 47.3 mg (46%). Mp: 165–170 °C; ^1^H-NMR (500 MHz, DMSO-*d*_6_) δ 9.57 (brs, 1H, Ar-NH), 7.83 (brs, 1H, CH_2_-N*H*), 7.68 (m, 1H, H-5’’’), 7.46 (m, 2H, H-3′, H-5′), 7.40 (m, 2H, H-2′, H-6′), 6.93 (m, 2H, H-6’’’, H-8’’’), 4.08 (t, 2H, *J*_H,H_ = 5.0 Hz, OCH_2_), 3.48 (brs, 2H, CH_2_N), 2.36 (s, 3H, CH_3_), 2.07 (s, 3H, CH_3_), 1.77 (quint, 2H, *J*_H,H_ = 5.0 Hz, CH_2_), 1.60 (quint, 2H, *J*_H,H_ = 10.0 Hz, CH_2_), 1.45 (quint, 2H, *J*_H,H_ = 6.0 Hz, CH_2_) ppm; ^13^C-NMR (125.7 MHz, DMSO-*d*_6_) δ 180.3 (C=S), 161.3, 160.7 (C-2’’’-C-7’’’), 153.1 (C-9’’’), 146.8 (C-4’’’), 138.8 (C-1′), 131.3 (C-3′, C-5′), 126.1 (C-5’’’), 124.1 (C-2′, C-6′), 117.8 (C-3’’’), 115.7 (C-4′), 112.3 (C-6’’’), 100.9 (C-8’’’), 68.1 (OCH_3_), 43.7 (NCH_3_), 29.5, 28.7, 24.4 (CH_2_), 18.2 (CH_3_), 14.5 (CH_3_) ppm; HR-ESIMS *m*/*z* calcd. for C_23_H_25_^79^BrN_2_NaO_3_S ([M + Na]^+^): 511.0661, found: 511.0656; C_23_H_25_^81^BrN_2_NaO_3_S ([M + Na]^+^): 513.0641, found: 513.0633.

1-(3′,5′-Difluorophenyl)-3-{5″-[(3’’’,4’’’-dimethyl-2’’’-oxo-2’’’*H*-chromen-7’’’-yl)oxy]pentyl}thiourea (**52**): Amine **25** (278.1 mg, 1.01 mmol, 1.0 equiv.), isothiocyanate **32** (518.6 mg, 3.03 mmol, 3.0 equiv.) and Et_3_N (139 µL, 1.01 mmol, 1.0 equiv.) were used, and the reaction was refluxed for 12 h. Compound **52** was obtained as a white solid. Yield: 31.7 mg (7%). Mp: 190–195 °C; ^1^H-NMR (300 MHz, DMSO-*d*_6_) δ 9.76 (brs, 1H, Ar-NH), 8.10 (brs, 1H, CH_2_-NH), 7.68 (m, 1H, H-5’’’), 7.26 (d, 2H, H-6’’’, H-8’’’), 6.95–6.84 (m, 3H, H-2′, H-4′, H-6′), 4.07 (t, 2H, *J*_H,H_ = 6.0 Hz, OCH_2_), 3.48 (m, 2H, CH_2_N), 2.35 (s, 3H, CH_3_), 2.06 (s, 3H, CH_3_), 1.77 (m, 2H, CH_2_), 1.62 (quint, 2H, *J*_H,H_ = 6.0 Hz, CH_2_), 1.46 (quint, 2H, *J*_H,H_ = 6.0 Hz, CH_2_) ppm; ^13^C-NMR (125.7 MHz, DMSO-*d*_6_) δ 180.1 (C=S), 162.1 (dd, ^1^*J*_C,F_ = 233.8 Hz, ^3^*J*_C,F_ = 15.4 Hz, C-3′, C-5′), 161.3, 160.7 (C-2’’’, C-7’’’), 153.1 (C-9’’’), 146.9 (C-4’’’), 142.4 (t, ^3^*J*_C,F_ = 13.0 Hz, C-1′), 126.1 (C-5’’’), 117.9 (C-3’’’), 113.5 (C-10’’’), 112.3 (C-6’’’), 104.4 (d, ^2^*J*_C,F_ = 26.4 Hz, C-2′, C-6′), 100.8 (C-8’’’), 98.3 (t, ^2^*J*_C,F_ = 25.8 Hz, C-4′), 68.1 (OCH_3_), 43.7 (NCH_2_), 28.2, 27.9, 22.9 (CH_2_), 14.9 (CH_3_), 12.9 (CH_3_) ppm; HR-ESIMS *m*/*z* calcd. for C_23_H_24_F_2_N_2_O_3_NaS ([M + Na]^+^): 469.1361, found: 469.1368.

1-(3′,5′-Dichlorophenyl)-3-{5″-[(3’’’,4’’’-dimethyl-2’’’-oxo-2’’’*H*-chromen-7’’’-yl)oxy]pentyl}thiourea (**53**): Amine **25** (115.6 mg, 0.42 mmol, 1.0 equiv.), isothiocyanate **33** (257.1 mg, 1.26 mmol, 3.0 equiv.) and Et_3_N (58 µL, 0.42 mmol, 1.0 equiv.) were used, and the reaction was refluxed for 15 h. Compound **52** was obtained as a grey solid. Yield: 85.5 mg (42%). Mp: 150–155 °C; ^1^H-NMR (300 MHz, DMSO-*d*_6_) δ 9.88 (brs, 1H, Ar-NH), 8.21 (brs, 1H, CH_2_-NH), 7.66 (m, 1H, H-5‴), 7.59 (m, 2H, H-2′, H-6′), 7.24 (brt, 1H, ^4^*J*_H,H_ = 2.0 Hz, H-4′), 6.93 (m, 2H, H-6’’’, H-8’’’), 4.07 (t, 2H, *J*_H,H_ = 6.3 Hz, OCH_2_), 3.48 (brs, 2H, CH_2_N), 2.35 (s, 3H, CH_3_), 2.06 (s, 3H, CH_3_), 1.77 (quint, 2H, *J*_H,H_ = 6.7 Hz, CH_2_), 1.61 (quint, 2H, *J*_H,H_ = 7.0 Hz, CH_2_), 1.47 (quint, 2H, *J*_H,H_ = 7.9 Hz, CH_2_) ppm; ^13^C-NMR (125.7 MHz, DMSO-*d*_6_) δ 180.2 (C=S), 161.2, 160.7 (C-2’’’, C-7’’’), 153.0 (C-9’’’), 146.8 (C-4’’’), 142.3 (C.1′), 133.5 (C-3′, C-5′), 126.1 (C-5’’’), 122.3, 119.9 (C-2′, C-4′, C-6′), 117.7 (C-3’’’), 113.5 (C-10’’’), 112.2 (C-6’’’), 100.8 (C-8’’’), 68.0 (OCH_2_), 45.6 (NCH_2_), 28.2, 27.9, 22.9 (CH_2_), 14.9 (CH_3_), 12.9 (CH_3_) ppm; HR-ESIMS *m*/*z* calcd. for C_23_H_24_^35^Cl_2_N_2_O_3_NaS ([M + Na]^+^): 501.0777, found: 501.0770.

1-(3′,5′-Dibromophenyl)-3-{5″-[(3’’’,4’’’-dimethyl-2’’’-oxo-2’’’*H*-chromen-7’’’-yl)oxy]pentyl}thiourea (**54**): Amine **25** (44.1 mg, 0.16 mmol, 1.0 equiv.), isothiocyanate **34** (140.6 mg, 0.48 mmol, 3.0 equiv.) and Et_3_N (22 µL, 0.16 mmol, 1.0 equiv.) were used, and the reaction was refluxed for 8 h. Compound **54** was obtained as a white solid. Yield: 28.8 mg (32%). ^1^H-NMR (500 MHz, DMSO-*d*_6_) δ 9.71 (brs, 1H, Ar-NH), 8.14 (brs, 1H, CH_2_-N*H*), 7.77 (s, 2H, H-2′, H-6′), 7.66 (m, 1H, H-5’’’), 7.46 (brs, 1H, H-4′), 6.93 (m, 2H, H-6’’’, H-8’’’), 4.07 (t, 2H, *J*_H,H_ = 6.3 Hz, OCH_2_), 3.49 (brs, 2H, CH_2_N), 2.35 (s, 3H, CH_3_), 2.07 (s, 3H, CH_3_), 1.77 (quint, 2H, *J*_H,H_ = 7.0 Hz, CH_2_), 1.61 (quint, 2H, *J*_H,H_ = 7.1 Hz, CH_2_), 1.46 (quint, 2H, *J*_H,H_ = 6.9 Hz, CH_2_) ppm; ^13^C-NMR (125.7 MHz, DMSO-*d*_6_) δ 180.2 (C=S), 161.3, 160.7 (C-2’’’, C-7’’’), 153.0 (C-9’’’), 146.8 (C-4’’’), 142.6 (C-1′), 127.8 (C-4′), 126.1 (C-5’’’), 123.4 (C-2′, C-6′), 121.8 (C-3′, C-5′), 117.8 (C-3’’’), 113.5 (C-10’’’), 112.3 (C-6’’’), 100.9 (C-8’’’), 68.1 (OCH_2_), 43.7 (NCH_2_), 28.2, 27.9, 23.0 (CH_2_), 14.9 (CH_3_), 12.9 (CH_3_) ppm; HR-ESIMS *m*/*z* calcd. for C_23_H_25_^79^Br_2_N_2_O_3_S ([M + H]^+^): 566.9947, found: 566.9940; C_23_H_25_^79^Br^81^BrN_2_O_3_S ([M + H]^+^)—568.9927, found—568.9919; C_23_H_25_^81^Br_2_ N_2_O_3_S ([M + H]^+^)—570.9906, found—570.9984.

1-(2′,4′-Difluorophenyl)-3-{5″-[(3’’’,4’’’-dimethyl-2’’’-oxo-2’’’*H*-chromen-7’’’-yl)oxy]pentyl}thiourea (**55**): Amine **25** (52.3 mg, 0.19 mmol, 1.0 equiv.), isothiocyanate **35** (97.6 mg, 0.57 mmol, 3.0 equiv.) and Et_3_N (26 µL, 0.19 mmol, 1.0 equiv.) were used, and the reaction was refluxed for 18 h. Compound **55** was obtained as a white solid. Yield: 34.5 mg (40%). Mp: 115–120 °C; ^1^H-NMR (500 MHz, DMSO-*d*_6_) δ 9.12 (brs, 1H, Ar-NH), 7.91 (brs, 1H, CH_2_-N*H*), 7.67 (m, 1H, H-5’’’), 7.57 (m, 1H, H-6′), 7.29 (brtd, 1H, *J*_5′,6′_ = ^3^*J*_5′,F_ = 9.6 Hz, *J*_3′,5′_ = 2.6 Hz, H-5′), 7.04 (brtd, 1H, ^3^*J*_3′_,_F-2_ = ^3^*J*_3′_,_F-4_ = 8.4 Hz, H-3′), 6.92 (m, 2H, H-6’’’, H-8’’’), 4.07 (t, 2H, *J*_H,H_ = 10.0 Hz, OCH_2_), 3.47 (brs, 2H, CH_2_N), 2.35 (s, 3H, CH_3_), 2.06 (s, 3H, CH_3_), 1.76 (m, 2H, CH_2_), 1.59 (quint, 2H, *J*_H,H_ = 6.0 Hz, CH_2_), 1.45 (quint, 2H, *J*_H,H_ = 6.0 Hz, CH_2_) ppm; ^13^C-NMR (125.7 MHz, DMSO-*d*_6_) δ 181.7 (C=S), 161.2, 160.7 (C-2’’’, C-7’’’), 156.6 (d, ^1^*J*_C,F_ = 246.7 Hz (C-2′ or C-4′), 153.0 (C-9’’’), 146.8 (C-4’’’), 129.8 (brs, C-6′), 126.1 (C-5’’’), 123.4 (brs, C-1′), 117.8 (C-3’’’), 113.5 (C-10’’’), 112.2 (C-6’’’), 111.0 (d, ^2^*J*_c,F_ = 23.9 Hz, C-5′), 104.2 (t, ^2^*J*_C3,F-2_ = ^2^*J*_C3,F-4_ = 25.2 Hz, C-3′), 100.9 (C-8’’’), 68.1 (OCH_3_), 44.0 (NCH_3_), 28.2 (x2), 22.9 (CH_2_), 14.9 (CH_3_), 12.9 (CH_3_) ppm; HR-ESIMS *m*/*z* calcd. for C_23_H_24_F_2_N_2_O_3_NaS ([M + Na]^+^): 469.13679, found: 469.1362.

1-(2′,4′-Dichlorophenyl)-3-{5″-[(3’’’,4’’’-dimethyl-2’’’-oxo-2’’’*H*-chromen-7’’’-yl)oxy]pentyl}thiourea (**56**): Amine **25** (57.8 mg, 0.21 mmol, 1.0 equiv.), isothiocyanate **36** (128.6 mg, 0.63 mmol, 3.0 equiv.) and Et_3_N (29 µL, 0.21 mmol, 1.0 equiv.) were used, and the reaction was refluxed for 18 h. Compound **56** was obtained as a brown solid. Yield: 55.2 mg (55%). Mp: 110–115 °C; ^1^H-NMR (500 MHz, DMSO-*d*_6_) δ 9.18 (brs, 1H, Ar-NH), 8.11 (brs, 1H, CH_2_-N*H*), 7.70–7.65 (m, 2H, H-6′, H-5’’’), 7.63 (d, 1H, *J*_3′,5′_ = 2.4 Hz, H-3′), 7.37 (dd, 1H, *J*_5′,6′_ = 8.6 Hz, H-5′), 6.93 (m, 2H, H-6’’’, H-8’’’), 4.07 (t, 2H, *J*_H,H_ = 5.0 Hz, OCH_2_), 3.49 (brs, 2H, CH_2_N), 2.35 (s, 3H, CH_3_), 2.07 (s, 3H, CH_3_), 1.77 (m, 2H, CH_2_), 1.59 (quint, 2H, *J*_H,H_ = 7.0 Hz, CH_2_), 1.46 (quint, 2H, *J*_H,H_ = 6.9 Hz, CH_2_) ppm; ^13^C-NMR (125.7 MHz, DMSO-*d*_6_) δ 181.1 (C=S), 161.2, 160.7 (C-2’’’, C-7’’’), 153.0 (C-9’’’), 146.8 (C-4’’’), 135.4 (C-1′), 130.2 (C-6′), 128.8 (C-3′), 127.2 (C-5′), 126.1 (C-5’’’), 117.8 (C-3’’’), 113.5 (C-10’’’), 112.2 (C-6’’’), 100.8 (C-8’’’), 68.1 (OCH_3_), 43.9 (NCH_3_), 28.2, 26.6, 22.9 (CH_2_), 14.9 (CH_3_), 12.9 (CH_3_) ppm; HR-ESIMS *m*/*z* calcd. for C_23_H_24_Cl_2_N_2_O_3_NaS ([M + Na]^+^): 501.0777, found: 501.0771.

1-(2′,4′-Dibromophenyl)-3-{5″-[(3’’’,4’’’-dimethyl-2’’’-oxo-2’’’*H*-chromen-7’’’-yl)oxy]pentyl}thiourea (**57**): Amine **25** (49.6 mg, 0.18 mmol, 1.0 equiv.), isothiocyanate **37** (158.2 mg, 0.54 mmol, 3.0 equiv.) and Et_3_N (25 µL, 0.18 mmol, 1.0 equiv.) were used, and the reaction was refluxed for 17 h. Compound **57** was obtained as a white solid. Yield: 49.9 mg (48%). Mp: 127–133 °C; ^1^H-NMR (500 MHz, DMSO-*d*_6_) δ 9.09 (brs, 1H, Ar-NH), 8.08 (brs, 1H, CH_2_-N*H*), 7.87 (s, 1H, H-3′), 7.67 (m, 1H, H-5’’’), 7.54 (m, 2H, H-5′, H-6′), 6.93 (m, 2H, H-6’’’, H-8’’’), 4.07 (t, 2H, *J*_H,H_ = 5.0 Hz, OCH_2_), 3.48 (brs, 2H, CH_2_N), 2.35 (s, 3H, CH_3_), 2.07 (s, 3H, CH_3_), 1.76 (m, 2H, CH_2_), 1.60 (m, 2H, CH_2_), 1.46 (m, 2H, CH_2_) ppm; ^13^C-NMR (125.7 MHz, DMSO-*d*_6_) δ 181.1 (C=S), 161.2, 160.7 (C-2’’’, C-7’’’), 153.0 (C-9’’’), 146.8 (C-4’’’), 137.1 (C-1′), 134.3 (C-3′), 130.9 (C-5′), 126.1 (C-5’’’), 121.1 (C-3’’’), 118.4 (C-2′ or C-4′), 117.7 (C-3’’’), 113.5 (C-10’’’), 112.4 (C-6’’’), 100.9 (C-8’’’), 68.1 (OCH_3_), 43.9 (NCH_3_), 28.2, 28.1, 22.9 (CH_2_), 14.9 (CH_3_), 12.9 (CH_3_) ppm; HR-ESIMS *m*/*z* calcd. for C_23_H_25_^79^Br_2_N_2_NaO_3_S ([M + Na]^+^): 588.9763, found: 588.9963; C_23_H_25_^79^Br^81^BrN_2_NaO_3_S ([M + Na]^+^): 590.9746, found: 590.9739; C_23_H_25_^81^Br_2_N_2_NaO_3_S ([M + Na]^+^): 592.9726, found: 592.9716.

1-(4′-Chloro-2′-fluorophenyl)-3-{5″-[(3’’’,4’’’-dimethyl-2’’’-oxo-2’’’*H*-chromen-7’’’-yl)oxy]pentyl}thiourea (**58**): Amine **25** (52.4 mg, 0.19 mmol, 1.0 equiv.), isothiocyanate **38** (106.9 mg, 0.57 mmol, 3.0 equiv.) and Et_3_N (26 µL, 0.19 mmol, 1.0 equiv.) were used, and the reaction was refluxed for 17 h. Compound **58** was obtained as a white solid. Yield: 36.2 mg (41%). Mp: 130–135 °C; ^1^H-NMR (500 MHz, DMSO-*d*_6_) δ 9.25 (brs, 1H, Ar-NH), 8.04 (brs, 1H, CH_2_-N*H*), 7.74 (t, 1H, *J*_5′,6′_ = ^4^*J*_6′,F_ = 8.2 Hz, H-6′), 7.68 (m, 1H, H-5‴), 7.46 (dd, 1H, *J*_3′,5′_ = 2.3 Hz, ^3^*J*_3′,F_ = 10.3 Hz, H-3′), 7.24 (m, 1H, H-5′), 6.95–6.90 (m, 2H, H-6‴, H-8‴), 4.08 (t, 2H, *J*_H,H_ = 6.4 Hz, OCH_2_), 3.48 (brs, 2H, CH_2_N), 2.36 (s, 3H, CH_3_), 2.07 (s, 3H, CH_3_), 1.77 (quint, 2H, *J*_H,H_ = 7.3 Hz, CH_2_), 1.59 (quint, 2H, *J*_H,H_ = 7.2 Hz, CH_2_), 1.45 (quint, 2H, *J*_H,H_ = 7.0 Hz, CH_2_) ppm; ^13^C-NMR (125.7 MHz, DMSO-*d*_6_) δ 181.1 (C=S), 161.2, 160.7 (C-2’’’, C-7’’’), 154.7 (C-9’’’), 155.6 (d, 1H, ^1^*J*_C,H_ = 247.7 Hz, C-2′), 146.8 (C-4’’’), 129.5 (C-6′), 128.8 (C-4′), 126.1 (C-5’’’), 124.2 (d, ^4^*J*_5′,F_ = 2.8 Hz, C-5′), 117.8 (C-3’’’), 116.3 (d, ^2^*J*_3′,F_ = 23.8 Hz, C-3′), 113.5 (C-10’’’), 112.3 (C-6’’’), 100.9 (C-8’’’), 68.1 (OCH_3_), 45.7 (NCH_3_), 28.2, 26.9, 22.8 (CH_2_), 14.9 (CH_3_), 12.9 (CH_3_) ppm; HR-ESIMS *m*/*z* calcd. for C_23_H_24_ClFN_2_NaO_3_S ([M + Na]^+^): 485.1072, found: 485.1067.

1-(2′-Bromo-4′-chlorophenyl)-3-{5″-[(3’’’,4’’’-dimethyl-2’’’-oxo-2’’’*H*-chromen-7’’’-yl)oxy]pentyl}thiourea (**59**): Amine **25** (63.3 mg, 0.23 mmol, 1.0 equiv.), isothiocyanate **39** (171.4 mg, 0.69 mmol, 3.0 equiv.) and Et_3_N (31 µL, 0.23 mmol, 1.0 equiv.) were used, and the reaction was refluxed for 18 h. Compound **59** was obtained as a grey solid. Yield: 49.7 mg (41%). Mp: 130–135 °C; ^1^H-NMR (500 MHz, DMSO-*d*_6_) δ 9.08 (brs, 1H, Ar-NH), 8.03 (brs, 1H, CH_2_-N*H*), 7.76 (d, 1H, *J*_H,H_ = 5.0 Hz, H-3′), 7.67 (m, 1H, H-5’’’), 7.59 (m, 1H,H-5′), 7.41 (m, 1H, H-6′), 6.93 (m, 2H, H-6’’’, H-8’’’), 4.07 (t, 2H, *J*_H,H_ = 5.0 Hz, OCH_2_), 3.49 (brs, 2H, CH_2_N), 2.36 (s, 3H, CH_3_), 2.07 (s, 3H, CH_3_), 1.75 (m, 2H, CH_2_), 1.60 (m, 2H, CH_2_), 1.46 (m, 2H, CH_2_) ppm; ^13^C-NMR (125.7 MHz, DMSO-*d*_6_) δ 181.1 (C=S), 161.2, 160.7 (C-2’’’, C-7’’’), 153.1 (C-9’’’), 146.8 (C-4’’’), 136.8 (C-1′), 132.9 (C-3′), 131.7, 130.6 (Ar-C), 127.7 (Ar-C), 126.1 (C-5’’’), 120.9 (Ar-C), 117.8 (C-3’’’), 113.5 (C-10’’’), 112.5 (C-6’’’), 100.9 (C-8’’’), 68.1 (OCH_3_), 43.9 (NCH_3_), 28.2, 26.6, 22.9 (CH_2_), 14.9 (CH_3_), 12.9 (CH_3_) ppm; HR-ESIMS *m*/*z* calcd. for C_23_H_24_^79^BrClN_2_NaO_3_S ([M + Na]^+^): 45.0272, found: 545.0258; C_23_H_24_^81^BrClN_2_NaO_3_S ([M + Na]^+^): 547.0251, found: 547.0232.

1-(4′-Chloro-2′-iodophenyl)-3-{5″-[(3’’’,4’’’-dimethyl-2’’’-oxo-2’’’*H*-chromen-7’’’-yl)oxy]pentyl}thiourea (**60**): Amine **25** (52.3 mg, 0.19 mmol, 1.0 equiv.), isothiocyanate **30** (168.4 mg, 0.57 mmol, 3.0 equiv.) and Et_3_N (26 µL, 0.19 mmol, 1.0 equiv.) were used, and the reaction was refluxed for 18 h. Compound **60** was obtained as a white solid. Yield: 21.0 mg (19%). Mp: 95–100 °C; ^1^H-NMR (500 MHz, DMSO-*d*_6_) δ 9.01 (brs, 1H, Ar-NH), 7.92 (m, 1H, H-3′), 7.88 (brs, 1H, CH_2_-N*H*), 7.69 (m, 1H, H-5’’’), 7.48–7.42 (m, 2H, H-5′, H-6′), 6.94 (m, 2H, H-6’’’, H-8’’’), 4.08 (t, 2H, *J*_H,H_ = 6.3 Hz, OCH_2_), 3.48 (brs, 2H, CH_2_N), 2.36 (s, 3H, CH_3_), 2.07 (s, 3H, CH_3_), 1.77 (quint, 2H, *J*_H,H_ = 6.3 Hz, CH_2_), 1.61 (m, 2H, CH_2_), 1.46 (m, 2H, CH_2_) ppm; ^13^C-NMR (125.7 MHz, DMSO-*d*_6_) δ 181.3 (C=S), 161.2, 160.7 (C-2’’’, C-7’’’), 153.0 (C-9’’’), 146.8 (C-4’’’), 137.6 (C-1′), 130.9, 130.3, 128.4 (Ar-C), 126.1 (C-5’’’), 117.8 (C-3’’’), 113.5 (C-10’’’), 112.3 (C-6’’’), 100.9 (C-8’’’), 68.1 (OCH_3_), 43.9 (NCH_3_), 28.3, 28.2, 22.9 (CH_2_), 14.9 (CH_3_), 12.9 (CH_3_) ppm; HR-ESIMS *m*/*z* calcd. for C_23_H_24_ClIN_2_NaO_3_S ([M + Na]^+^): 593.0133, found: 593.0132.

### 3.2. CA Inhibition Assays

The same conditions as previously reported were used [47]. An applied photophysics stopped-flow instrument was used for assaying the CA-catalyzed CO_2_ hydration activity [57]. Phenol red (at 0.2 mM) was used as an indicator, working at the absorbance maximum of 557 nm, with 20 mM Hepes (pH 7.4) and 20 mM Na_2_SO_4_ (for maintaining constant ionic strength), following the initial rates of the CA-catalyzed CO_2_ hydration reaction for 10–100 s. The CO_2_ concentrations ranged from 1.7 to 17 mM for the determination of the kinetic parameters and inhibition constants. For each inhibitor, at least six traces of the initial 5–10% of the reaction were used for determining the initial rate. The uncatalyzed rates were determined in the same manner and subtracted from the total observed rates. Stock solutions of inhibitor (10 mM) were prepared in distilled–deionized water, and appropriate dilutions were done thereafter with distilled–deionized water. AAZ was used as a positive control. Inhibitor and enzyme solutions were preincubated together for 15 min at room temperature before assay in order to allow for the formation of the E–I complex. The inhibition constants were obtained by nonlinear least-squares methods using PRISM 3 and the Cheng–Prusoff equation and represent the mean from at least three different determinations. All CA isoforms were recombinant ones (5–12 nM), obtained in-house.

### 3.3. Docking Simulations

The crystallographic structures of CA IX (PDB: 5FL4) and CA XII (PDB: 5MSA with a resolution of 1.82 Å and 1.20 Å (PDB ID: 6QAA) were obtained from the Protein DataBank [58]. Docking simulations were performed in MOE Software (version 2019.01, Chemical Computing Group, ULC, Montreal, QC, Canada). Both proteins were optimized through the QuickPrep protocol. Briefly, crystallographic artefacts, non-bonded ligands and dimeric copies of the protein were removed. Water molecules at a maximum distance of 4.5 Å from the active site were maintained, and the rest were eliminated. Thereafter, all ligands were drawn, hydrogens added and geometry optimized through energy minimization.

During docking simulations, ligands were placed in the grid of the co-crystallized ligand. In the placement stage, energy binding calculations used the Triangle Matcher algorithm with the London dG scoring scheme. In the refinement stage, the receptor was kept rigid and the GBVI/WSA dg scoring scheme was used.

### 3.4. Antiproliferative Activity

#### 3.4.1. Cell Lines and Culture

The same conditions as previously reported were used [47]. The human cancer cell lines A549, HBL-100, and T-47D, as well as HeLa, were provided by Dr. Raimundo Freire (Hospital Universitario de Canarias, Tenerife, Canary Islands). The lung cancer cell lines SW1573 and WiDr were provided by Prof. G. J. Peters (VU University Medical Center, Amsterdam, The Netherlands). Cells were grown in RPMI-1640 medium containing 5% fetal bovine serum (FBS), 2 mM l-glutamine, 100 U/mL of penicillin G, and 0.1 mg/mL of streptomycin at 37 °C in a 95% humidified atmosphere of 5% CO_2_. Cells were maintained in culture in 60 mm cell culture dishes in growth medium (10 mL) and passaged twice weekly.

#### 3.4.2. Antiproliferative Tests

The same conditions as previously reported were used [47,59]. The antiproliferative activity of compounds was tested using our implementation of the protocol of the National Cancer Institute (NCI) of the USA. The following seeding densities (cells per well) were used: 2500 (A549, HBL-100, HeLa, and SW1573) and 5000 (T-47D and WiDr). Stock solutions of inhibitors (40 mM) were prepared in pure DMSO (400 times the maximum test concentration). For each test compound, the cells were exposed for a period of 48 h to serial decimal dilutions in cell culture medium of the test compounds (0.001–100 μM). For each product, GI_50_ values were calculated according to the NCI formulas (n = 3; data are expressed as mean ± SD).

#### 3.4.3. Cell Morphology

The same conditions as previously reported were used [47]. The CX-A imaging platform microscope (CX-A, Nanolive S.A., Lausanne, Switzerland) was used to measure refractive indices, creating a holotomographic 3D image of the cells. SW1573 cells were seeded onto 35 mm cell culture imaging dishes (IBIDI GmbH, Gräfelfing, Germany) at a density of 50,000 cells/well. On the next day, treated cells were exposed to the test compounds right before the acquisition of the images. Image data were transferred to FIJI software v2.9.0 (NIH, USA) for image analysis. EVE software v2.2.1.2162 (Nanolive S.A., Tolochenaz, Switzerland) was used for the analysis of the refractive indices and calculation of the phenotypic parameters. Specific conditions used in this work comprised an exposure dose of 10 μM and 20 h of monitoring at intervals of 10 min.

## 4. Conclusions

The hybridization of coumarin and *N*-aryl thiourea scaffolds at the C-7 position of the chromenone has proven to be a highly effective strategy for the development of new agents targeting the tumour-associated carbonic anhydrases IX and XII. Through a systematic exploration of structural modifications, including C-3/C-4 substitution on the coumarin core, variation in the tether length, chalcogen replacement, and controlled halogenation of the terminal aryl ring, we established solid structure–activity relationships that guided the identification of the most promising inhibitors within the series. In particular, the optimal combination of a dimethyl-substituted coumarin core, a pentyl linker, and an *N*-aryl thiourea moiety afforded compounds with remarkable potency and selectivity.

The lead molecule exhibited inhibition constants in the low- to mid-nanomolar range for CA IX and CA XII, significantly outperforming the reference drug acetazolamide in terms of selectivity. Molecular docking studies reinforced these findings by demonstrating well-defined and energetically favourable interactions and positioning for the *Z*-configured *o*-hydroxycinnamic acid derivatives, obtained by the sterase activity of CAs.

Beyond CA inhibition, the compounds also demonstrated potent antiproliferative activity across a panel of human cancer cell lines, including multidrug-resistant models. The observation that thioureas **45** and **46** achieved GI_50_ values comparable to or even superior to clinically used agents such as cisplatin underscores the translational potential of this chemotype. Importantly, label-free 3D holotomographic microscopy revealed that the mechanism of cytotoxicity involves a delayed induction of apoptosis, suggesting that CA inhibition may intersect with additional molecular pathways relevant to tumour survival.

Taken together, these results not only validate coumarin–thiourea hybrids as a compelling platform for CA IX/XII inhibition but also position them as promising leads with both enzymatic and antiproliferative effects. The high degree of selectivity toward tumour-associated isoforms, combined with limited activity against off-target cytosolic CAs I and II, further supports their potential for safer therapeutic development.

Overall, this work provides a strong foundation for the continued evolution of coumarin-based CA inhibitors and reinforces the value of a hybrid, multi-pharmacophore design in modern anticancer drug discovery. The selective inhibition of CA IX and CA XII has direct therapeutic implications, as both isoforms contribute to tumour acidification, metastasis, and resistance to hypoxia. The potent activity displayed by compounds **45** and **46** suggests that coumarin–thiourea hybrids may serve as valuable leads for the development of targeted anticancer agents. However, future translational work should include evaluation of bioavailability, metabolic stability, and potential toxicity in non-tumour tissues. Additionally, regulatory development of carbonic anhydrase inhibitors typically requires early assessment of safety due to the numerous physiological roles of CA isoforms, underscoring the importance of comprehensive off-target profiling.

## Figures and Tables

**Table 3:** Binding energies in the docking simulations of the interactions between **46** and CAIX/CAXII.

Protein	Binding Energy (Kcal/mol)
**CAIX**	−9.34
**CAXII**	−10.03

## Data Availability

The original contributions presented in this study are included in the article/Supplementary Material. Further inquiries can be directed to the corresponding author(s).

## Supplementary Materials

The following supporting information can be downloaded at: https://www.mdpi.com/article/10.3390/ijms27093743/s1.

## Abbreviations

The following abbreviations are used in this manuscript:

AAZ	Acetazolamide
br	Broad singlet
brt	Broad triplet
brtd	Broad triplet of doublets
CA	Carbonic anhydrase
calcd.	Calculated
CARP	Carbonic anhydrase-related proteins
d	Doublet
dd	Doublet of doublets
ddd	Doublet of doublet of doublets
E-I	Enzyme–inhibitor
equiv.	Equivalents
ESIMS	Electrospray ionization mass spectrometry
GI_50_	Growth inhibition at 50%
hCA	Human carbonic anhydrase
HR	High resolution
m	Multiplet
mp	Melting point
q	Quartet
quint	Quintet
s	Singlet
SAR	Structure–activity relationship
S.I.	Selectivity index
t	Triplet
TLC	Thin-layer chromatography
