# Supplementary material for: Coumarin–Thiourea Hybrids: Structural Features Governing CA Inhibition and Antiproliferative Effects
Source: Int J Mol Sci. 2026 Apr 23;27(9):3743. doi: 10.3390/ijms27093743 (PMC13163854; doi:10.3390/ijms27093743)
Supplement: Supplementary file 1 [file ijms-27-03743-s001.zip › ijms-4191070-supplementary.pdf]

Coumarin–Thiourea Hybrids: Structural Features Governing CA Inhibition and Antiproliferative Effects

Alma Fuentes-Aguilar [1,2,†], Rebecca Colombo [1,3,†], Aday González-Bakker [4], Adrián Puerta [4], Penélope Merino-Montiel [2], Sara Montiel-Smith [2], José L. Vega-Báez [2], Simone Giovannuzzi [3], Alessio Nocentini [3], José G. Fernández-Bolaños [1], Claudiu T. Supuran [3], José M. Padrón [4] and Óscar López [1,*]

[1] Departamento de Química Orgánica, Facultad de Química, Universidad de Sevilla, Apartado 1203, E-41071 Seville, Spain; bolanos@us.es (J.G.F.-B.)
[2] Facultad de Ciencias Químicas, Ciudad Universitaria, Benemérita Universidad Autónoma de Puebla, Puebla 72570, Mexico; maria.montiel@correo.buap.mx (S.M.-S.)
[3] NEUROFARBA Department, Sezione di Scienze Farmaceutiche e Nutraceutiche, University of Florence, 50019 Florence, Italy; claudiu.supuran@unifi.it (C.T.S.)
[4] BioLab, Instituto Universitario de Bio-Orgánica "Antonio González" (IUBO-AG), Universidad de La Laguna, c/Astrofísico Francisco Sánchez 2, E-38206 La Laguna, Spain; jmpadron@ull.es (J.M.P.)
* Correspondence: osc-lopez@us.es
† These authors contributed equally to this work.

^{1}H-, ^{13}C-NMR and HR-ESIMS spectra of compounds **47–60 (Figures S1-S42)**……………………………………………………………………...2–43

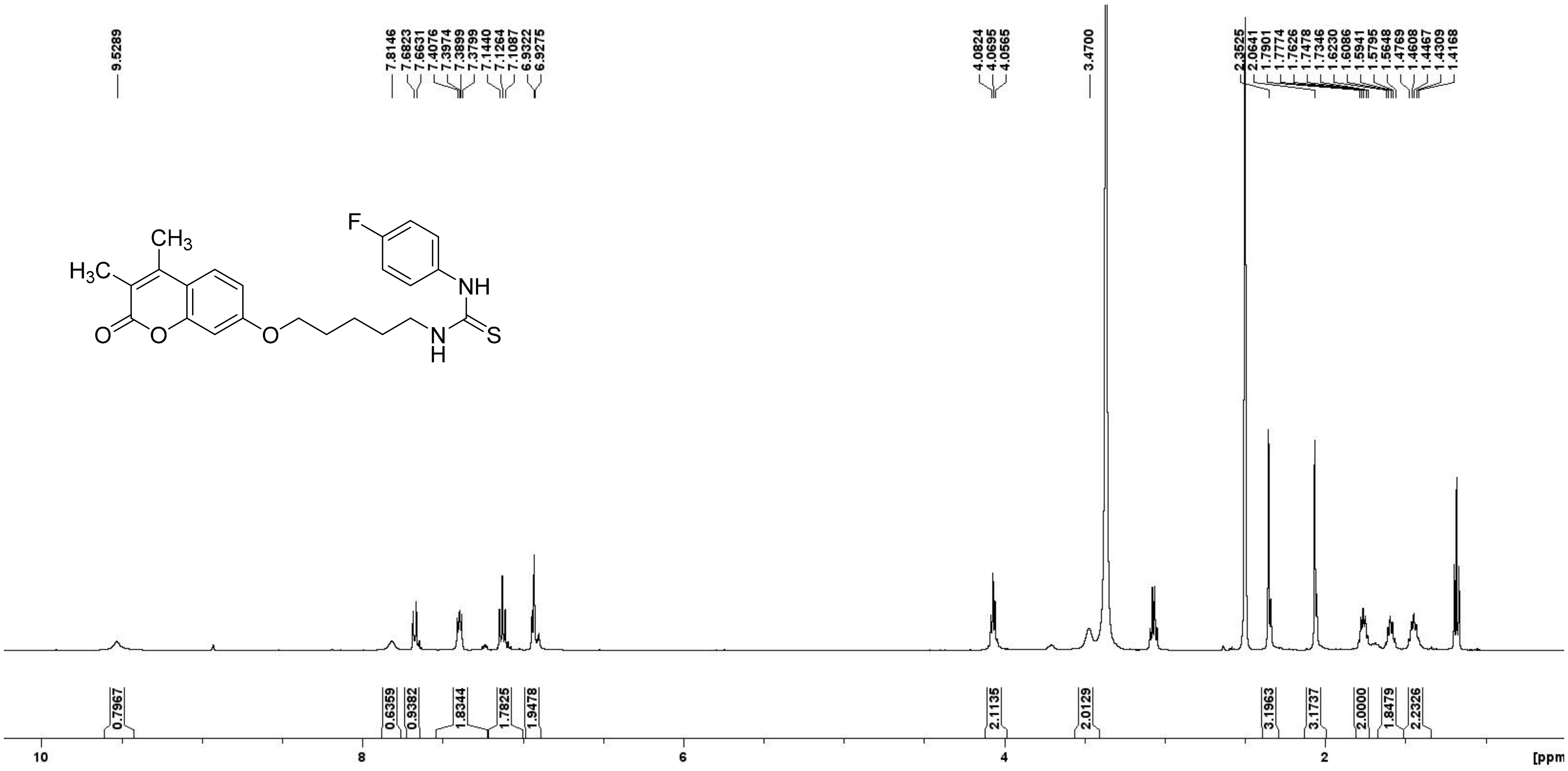

Figure S1. ^{1}H-NMR spectrum (500 MHz, DMSO-d_6) of **47**

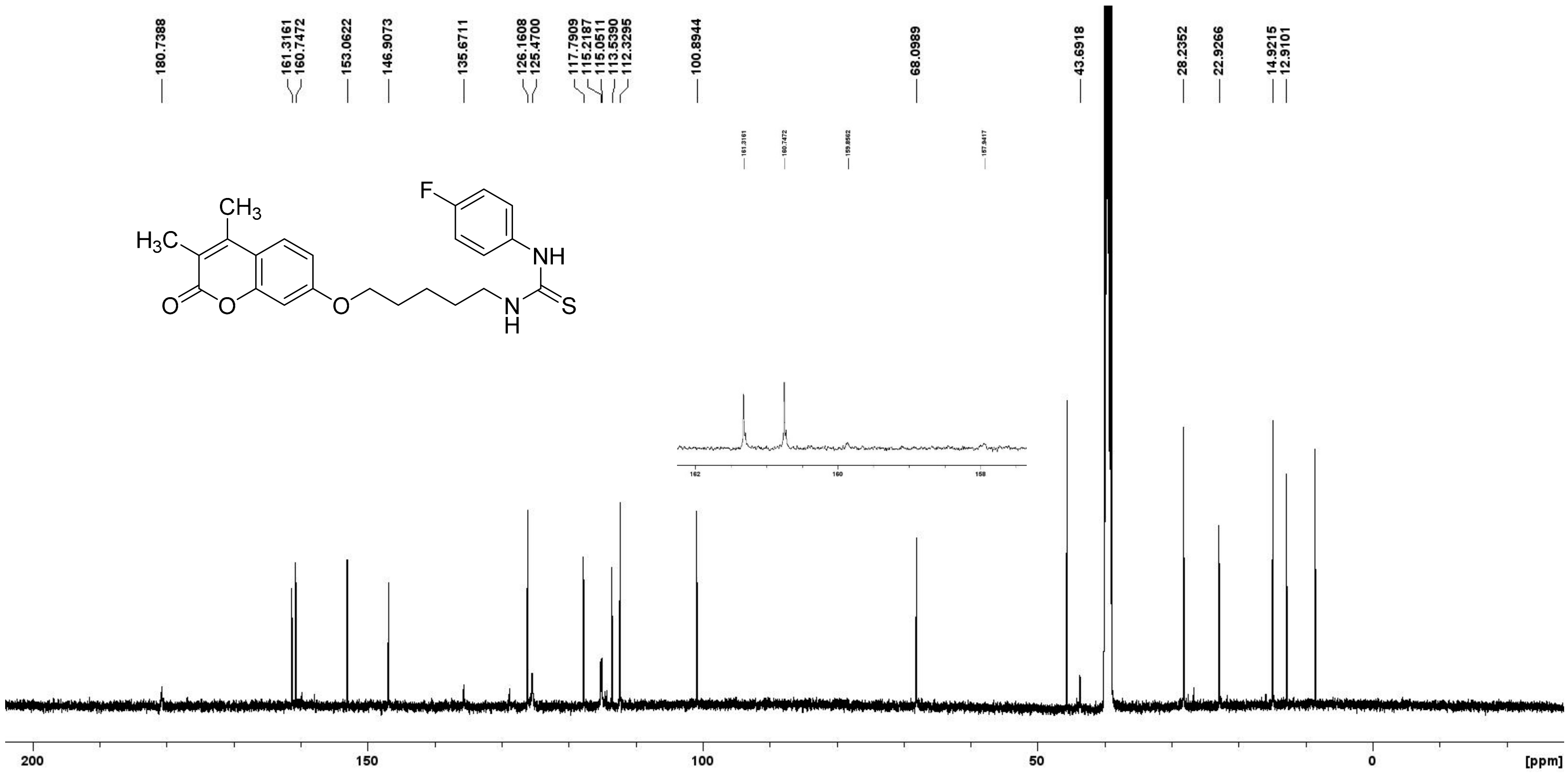

Figure S2. ^{13}C-NMR spectrum (125.7 MHz, DMSO-d_6) of **47**

Centro de Investigación, Tecnología e Innovación
Universidad de Sevilla
Avda. Reina Mercedes, nº 4-B, 41012 Sevilla, Spain

SERVICIO DE ESPECTROMETRIA DE MASAS

INFORMACIÓN EXPERIMENTAL

Equipo: Elite	Fuente ionización: HESI	Modo: POSITIVO	Rango de masas: 60-900

230127_RC53S 01/27/23 13:55:13 RC53(S) PM=428 C23H25FN2O3S

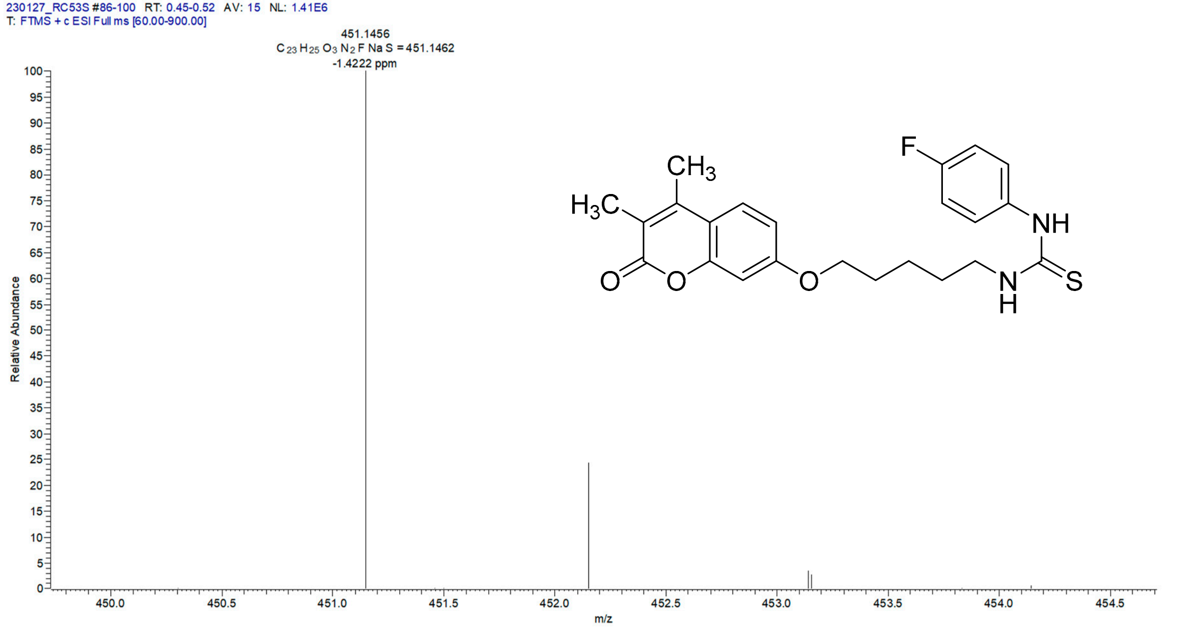

SGI Espectrometría de Masas
Tfno. 954559744; espectrometriademasas@us.es
Apdo. 1152, 41080 Sevilla, Spain
PNT07EPM0001-FT08
Rev 00
Fecha:25/05/2018

1

Figure S3. HRESI-MS of **47**

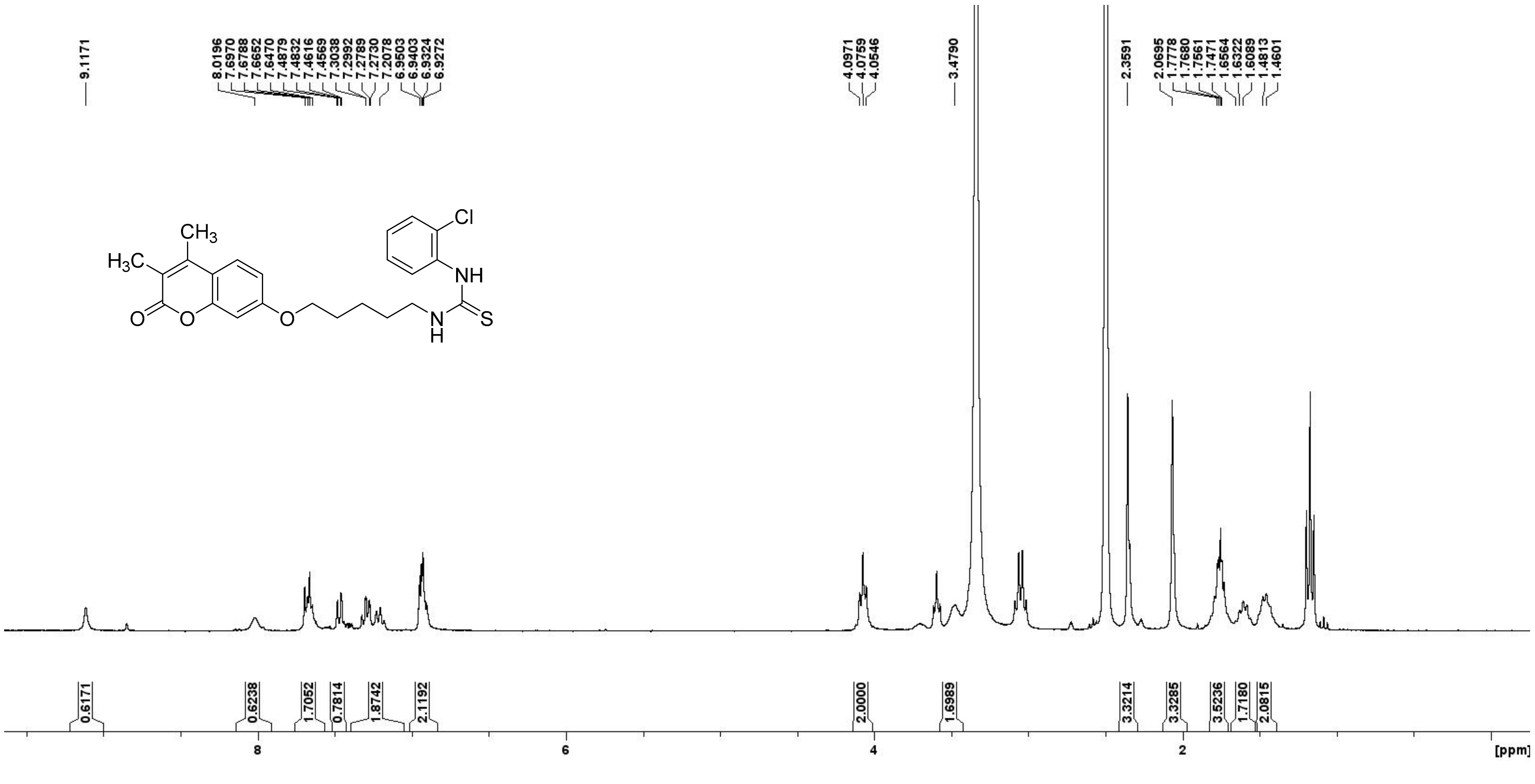

Figure S4. ^{1}H-NMR spectrum (300 MHz, DMSO-d_6) of **48**

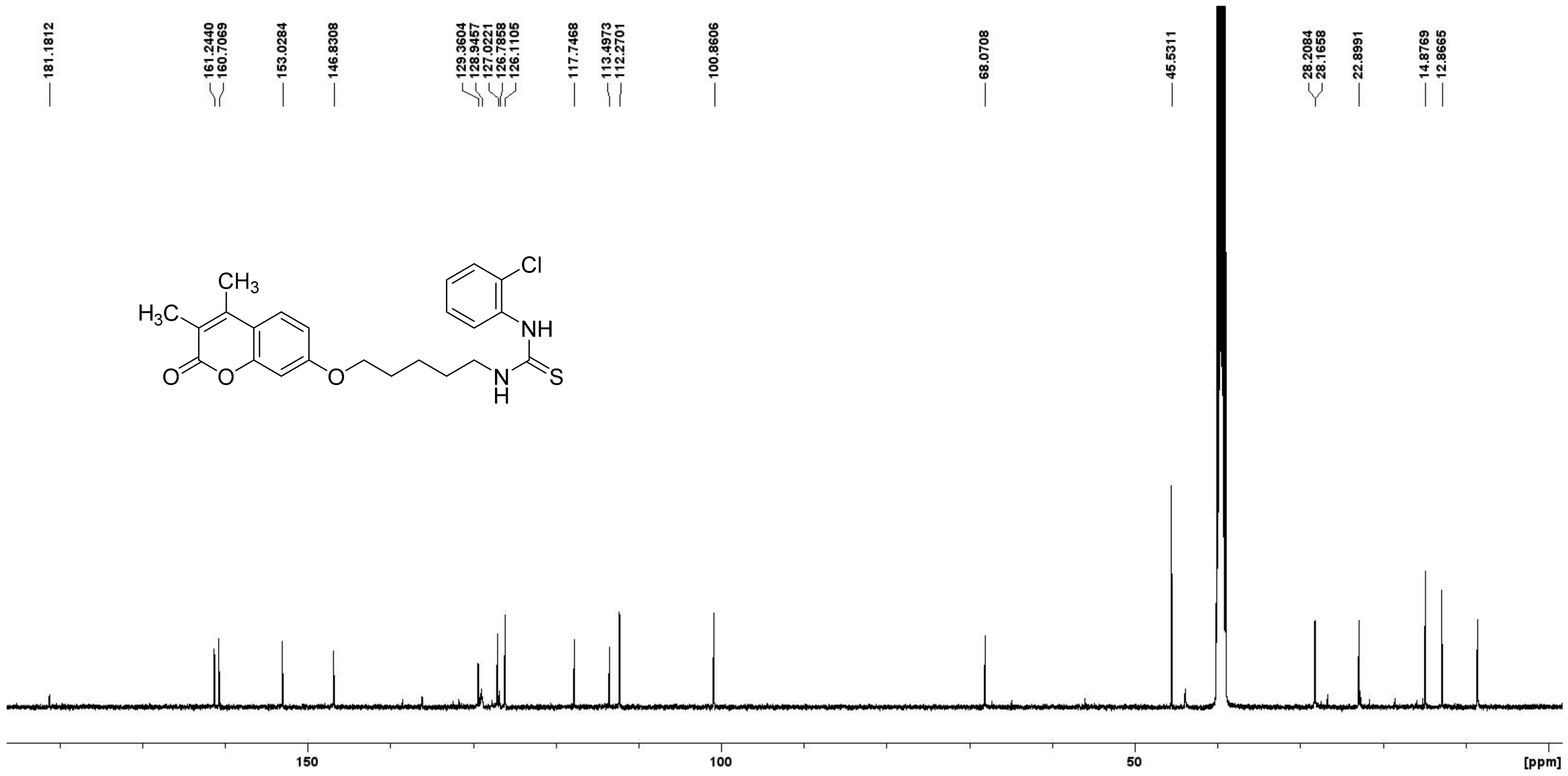

Figure S5. ^{13}C-NMR spectrum (125.7 MHz, DMSO-d_6) of **48**

INFORMACIÓN EXPERIMENTAL

Equipo: Elite	Fuente ionización: HESI	Modo: POSITIVO	Rango de masas: 60-900

230126_RC52S 01/26/23 11:57:00 RC52(S) PM=444 C23H25ClN2O3S

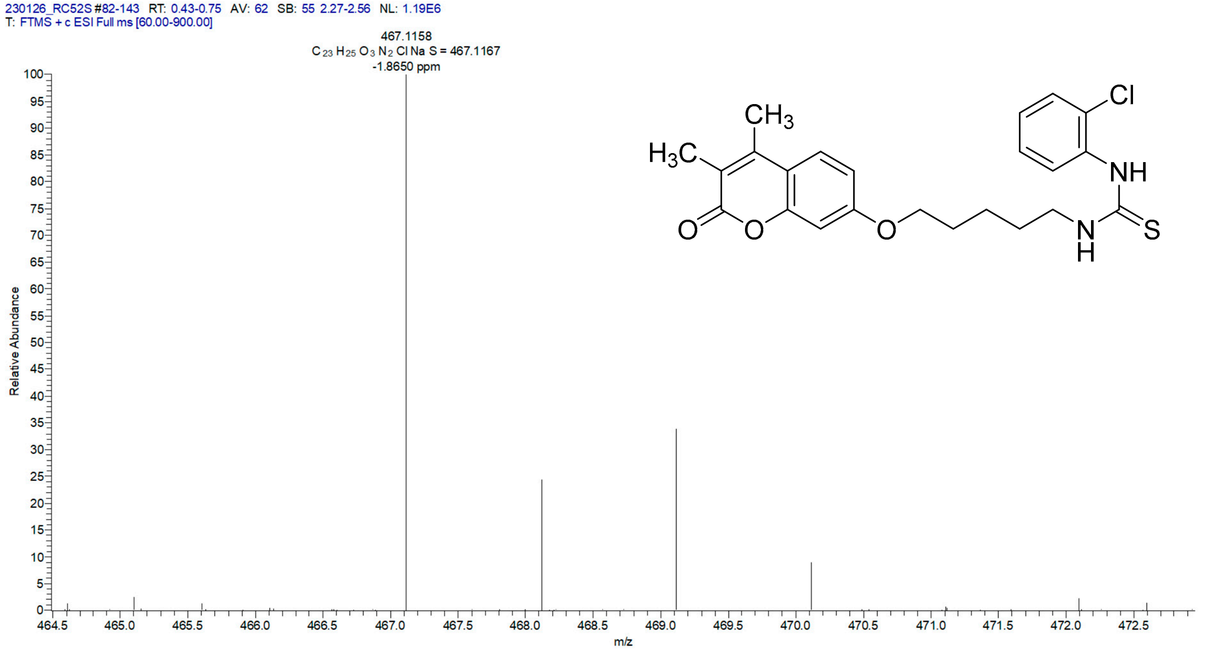

Figure S6. HRESI-MS of **48**

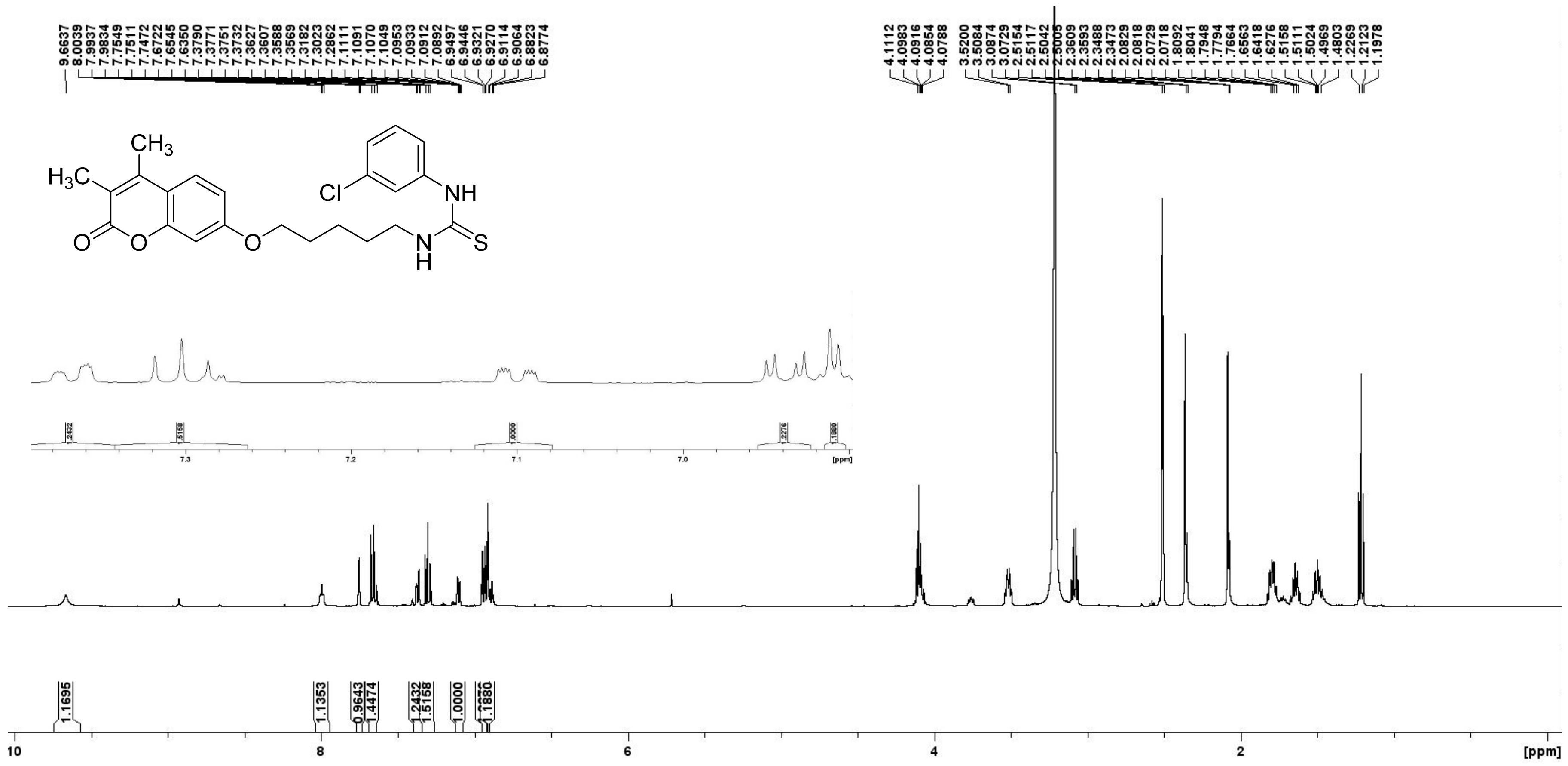

Figure S7. ^{1}H-NMR spectrum (500 MHz, DMSO-d_6) of **49**

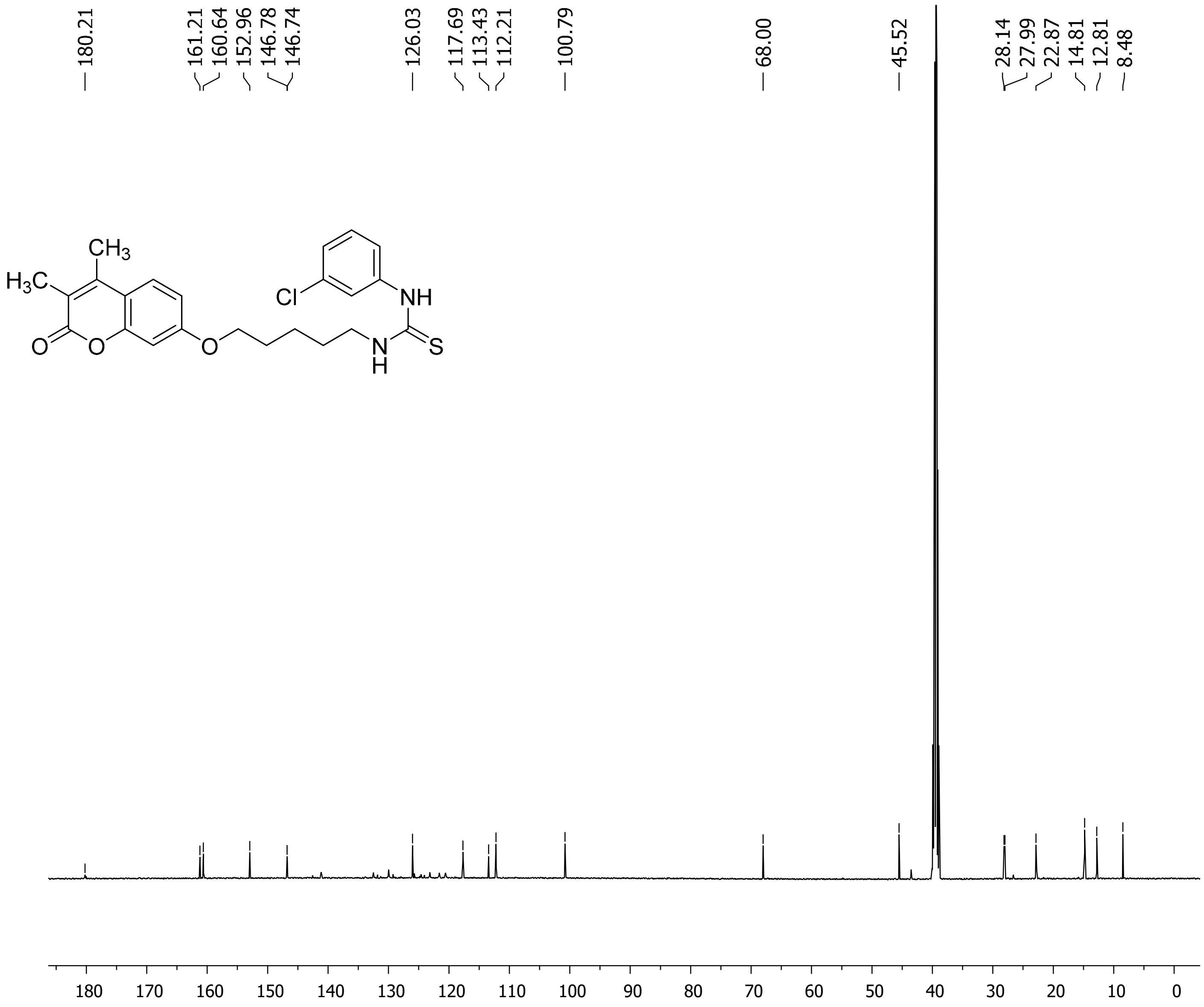

Figure S8. ^{13}C-NMR spectrum (125.7 MHz, DMSO-d_6) of **49**

INFORMACIÓN EXPERIMENTAL

Equipo: Elite	Fuente ionización: HESI	Modo: POSITIVO	Rango de masas: 60-900

230126_RC40S 01/26/23 11:41:37 RC40(S) PM=444 C23H25ClN2O3S

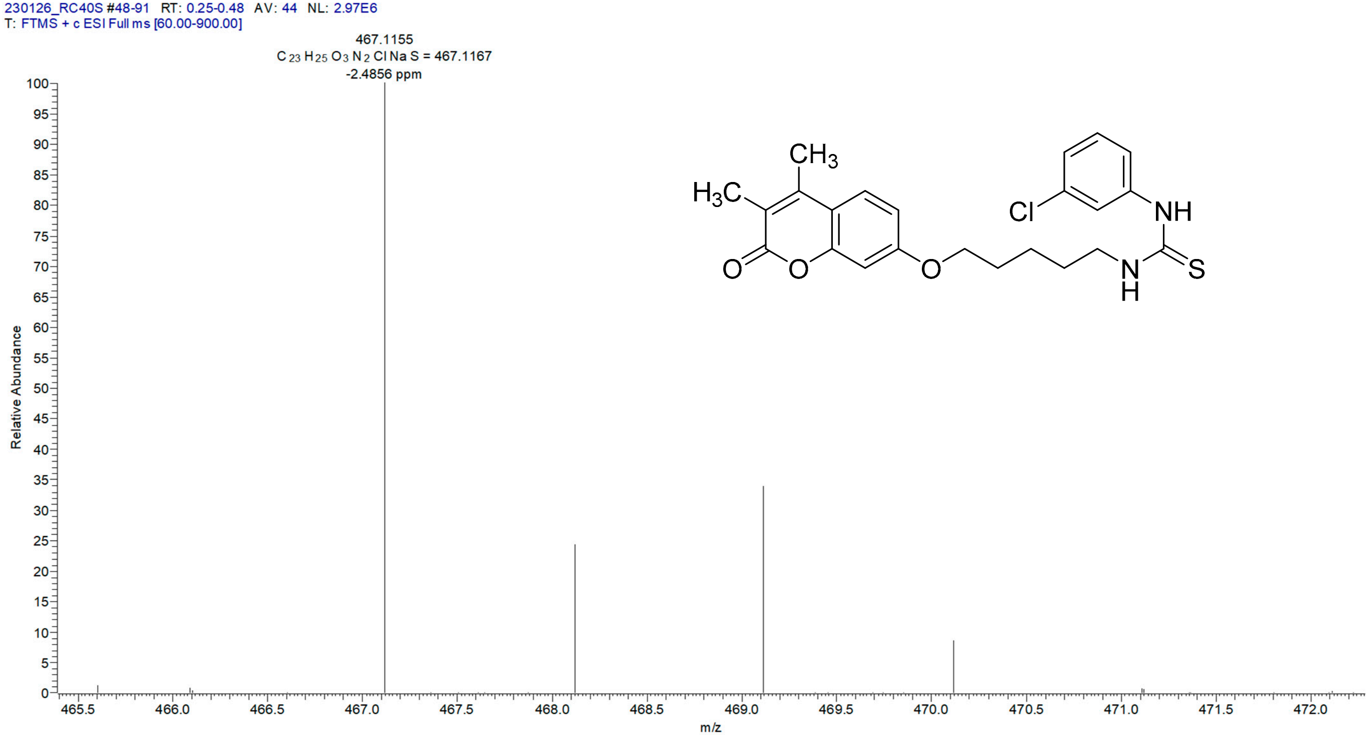

Figure S9. HRESI-MS of **49**

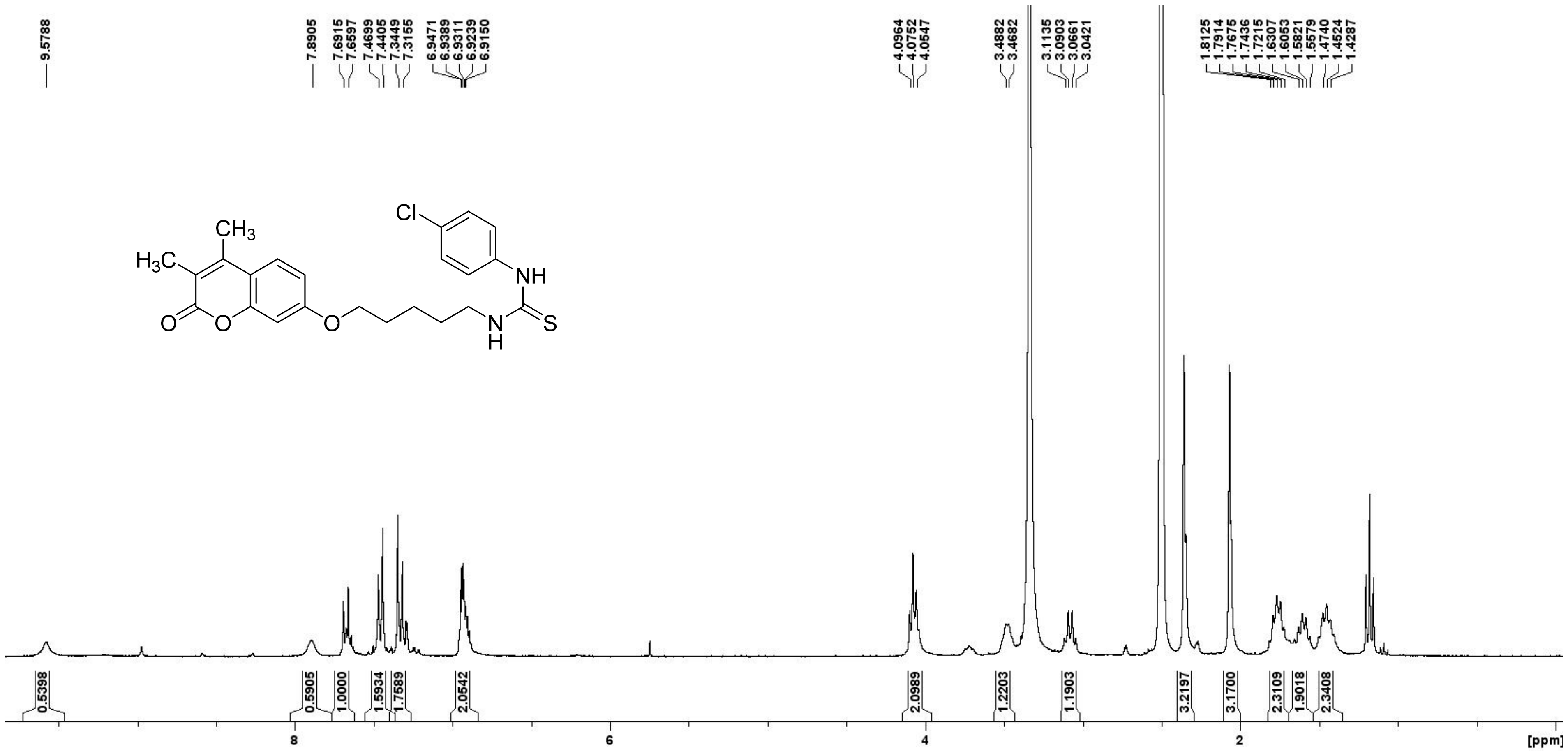

Figure S10. ^{1}H-NMR spectrum (300 MHz, DMSO-d_6) of **50**

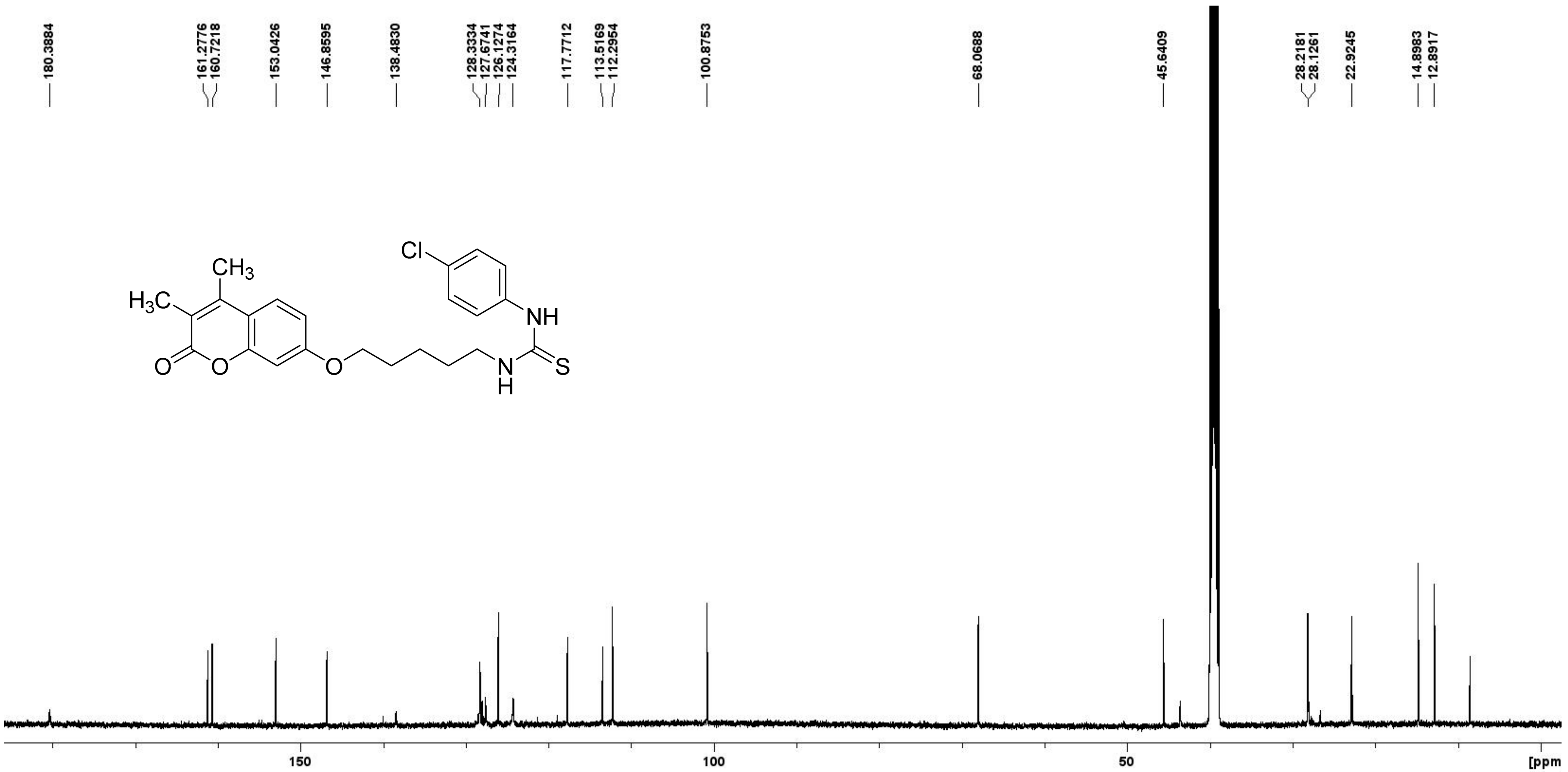

Figure S11. ^{13}C-NMR spectrum (125.7 MHz, DMSO-d_6) of **50**

Centro de Investigación, Tecnologia e Innovación
Universidad de Sevilla
Avda. Reina Mercedes, nº 4-B, 41012 Sevilla, Spain

SERVICIO DE ESPECTROMETRIA DE MASAS

INFORMACIÓN EXPERIMENTAL

Equipo: Elite	Fuente ionización: HESI	Modo: POSITIVO	Rango de masas: 60-900

230126_RC46S 01/26/23 11:46:44 RC46(S) PM=444 C23H25ClN2O3S

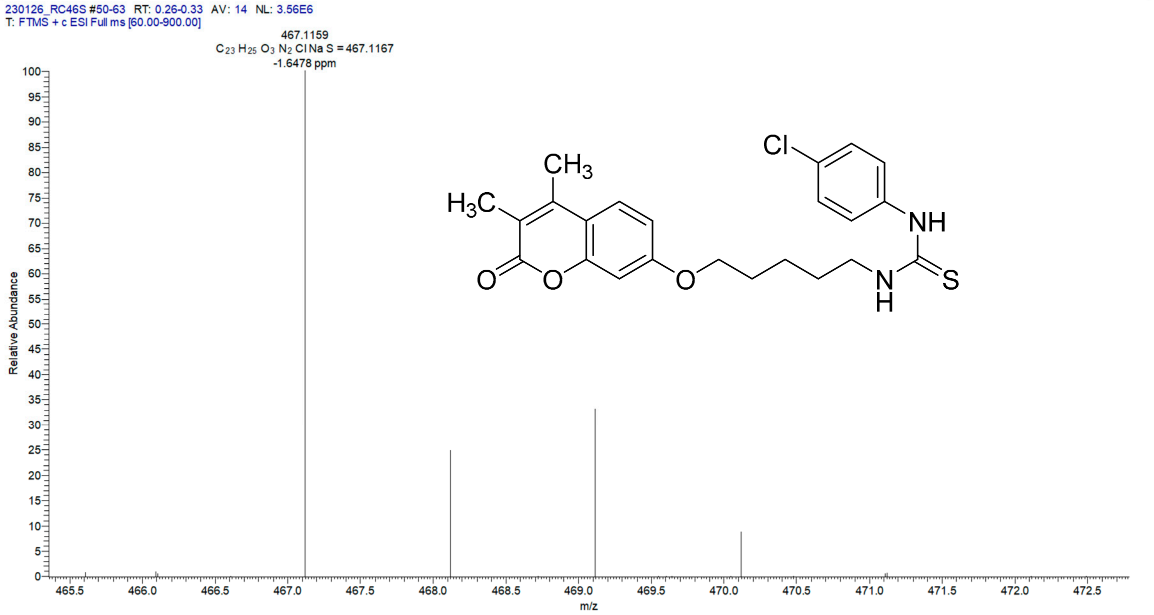

SGI Espectrometria de Masas
Tfno. 954559744; espectrometriademasas@us.es
Apdo. 1152, 41080 Sevilla, Spain
PNT07EPM0001-FT08
Rev 00
Fecha:25/05/2018

4

Figure S12. HRESI-MS of **50**

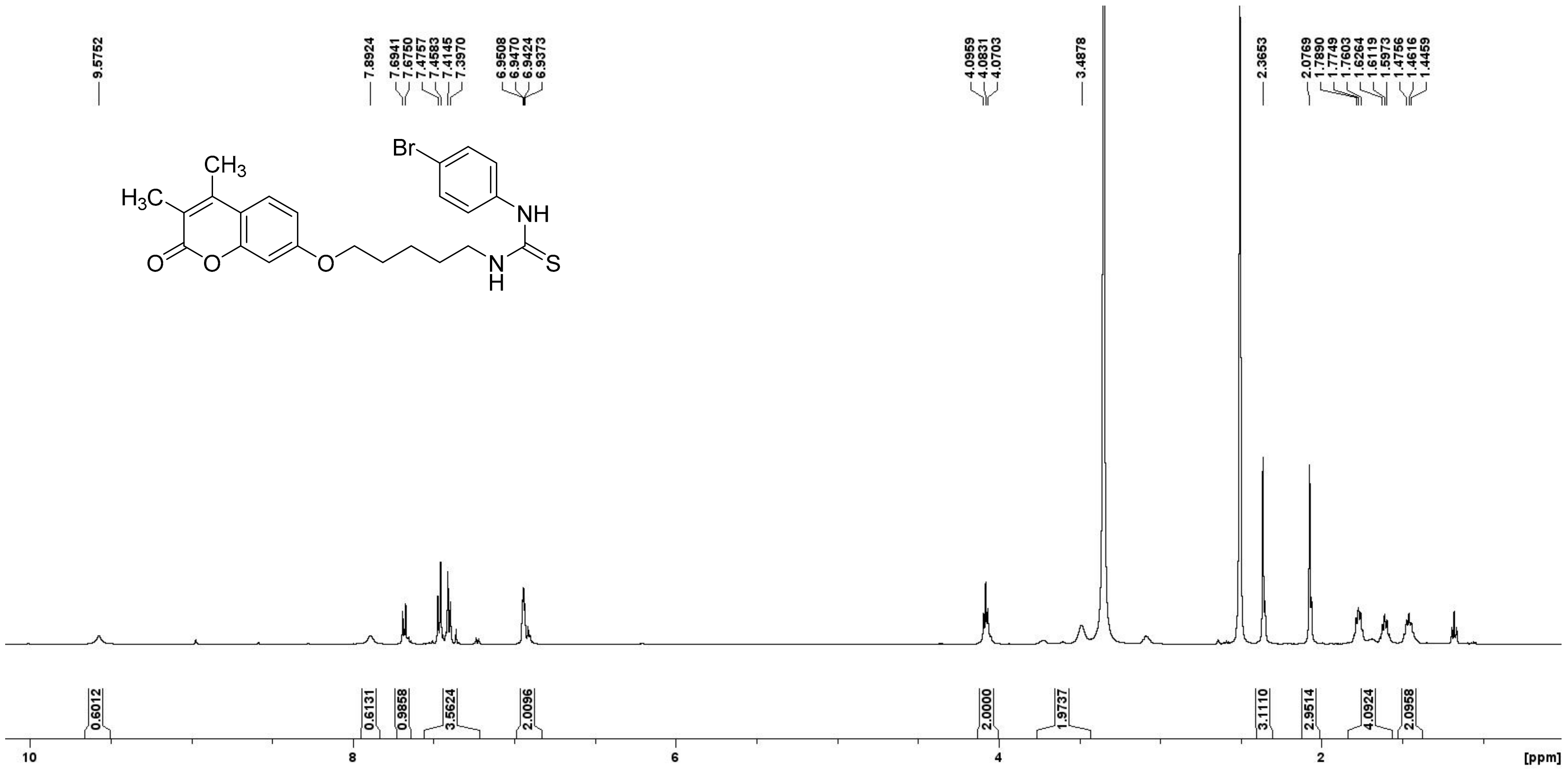

Figure S13. ^{1}H-NMR spectrum (500 MHz, DMSO-d_6) of **51**

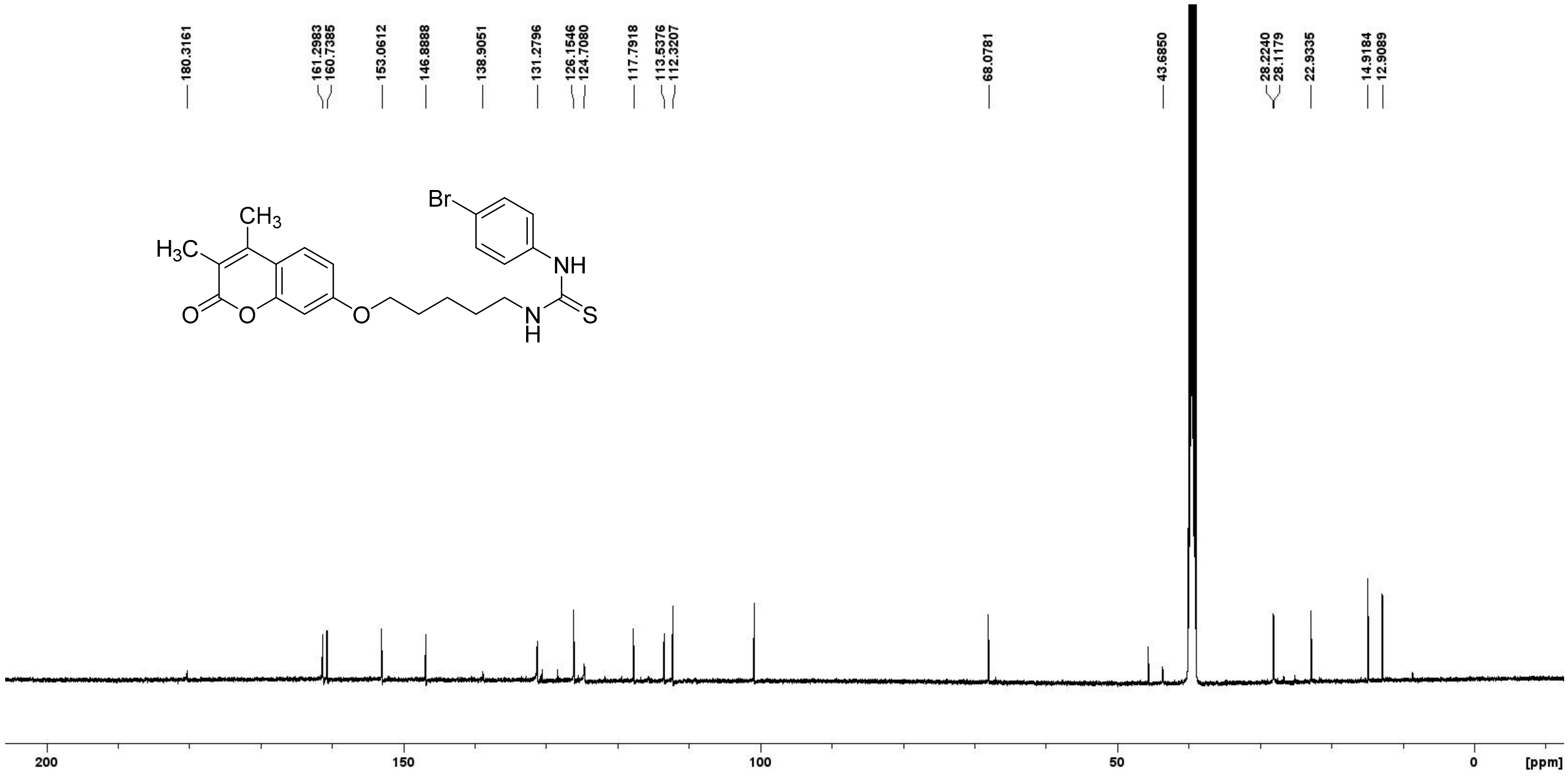

Figure S14. ^{13}C-NMR spectrum (125.7 MHz, DMSO-d_6) of **51**

Centro de Investigación, Tecnología e Innovación
Universidad de Sevilla
Avda. Reina Mercedes, nº 4-B, 41012 Sevilla, Spain

SERVICIO DE ESPECTROMETRIA DE MASAS

INFORMACIÓN EXPERIMENTAL

Equipo: Elite	Fuente ionización: HESI	Modo: POSITIVO	Rango de masas: 60-900

230127_RC63S 01/27/23 14:00:21 RC63(S) PM=488 C23H25BrN2O3S

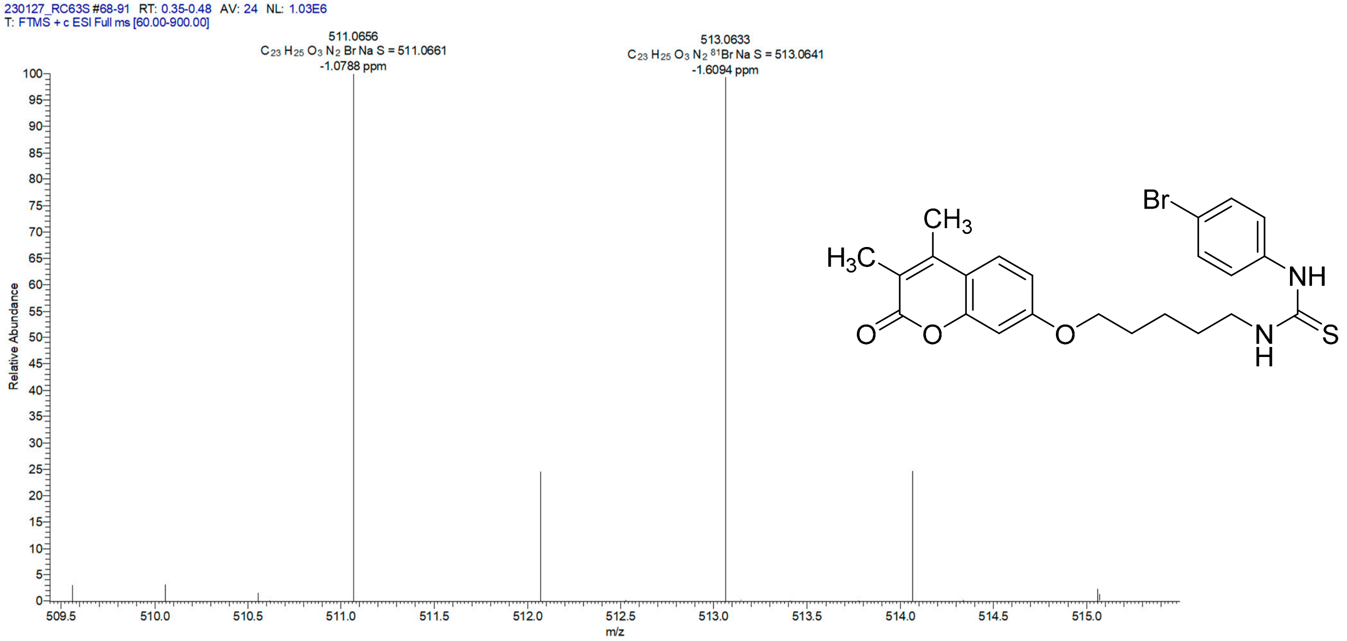

SGI Espectrometria de Masas
Tfno. 954559744; espectrometriademasas@us.es
Apdo. 1152, 41080 Sevilla, Spain
PNT07EPM0001-FT08
Rev 00
Fecha:25/05/2018

2

Figure S15. HRESI-MS of **51**

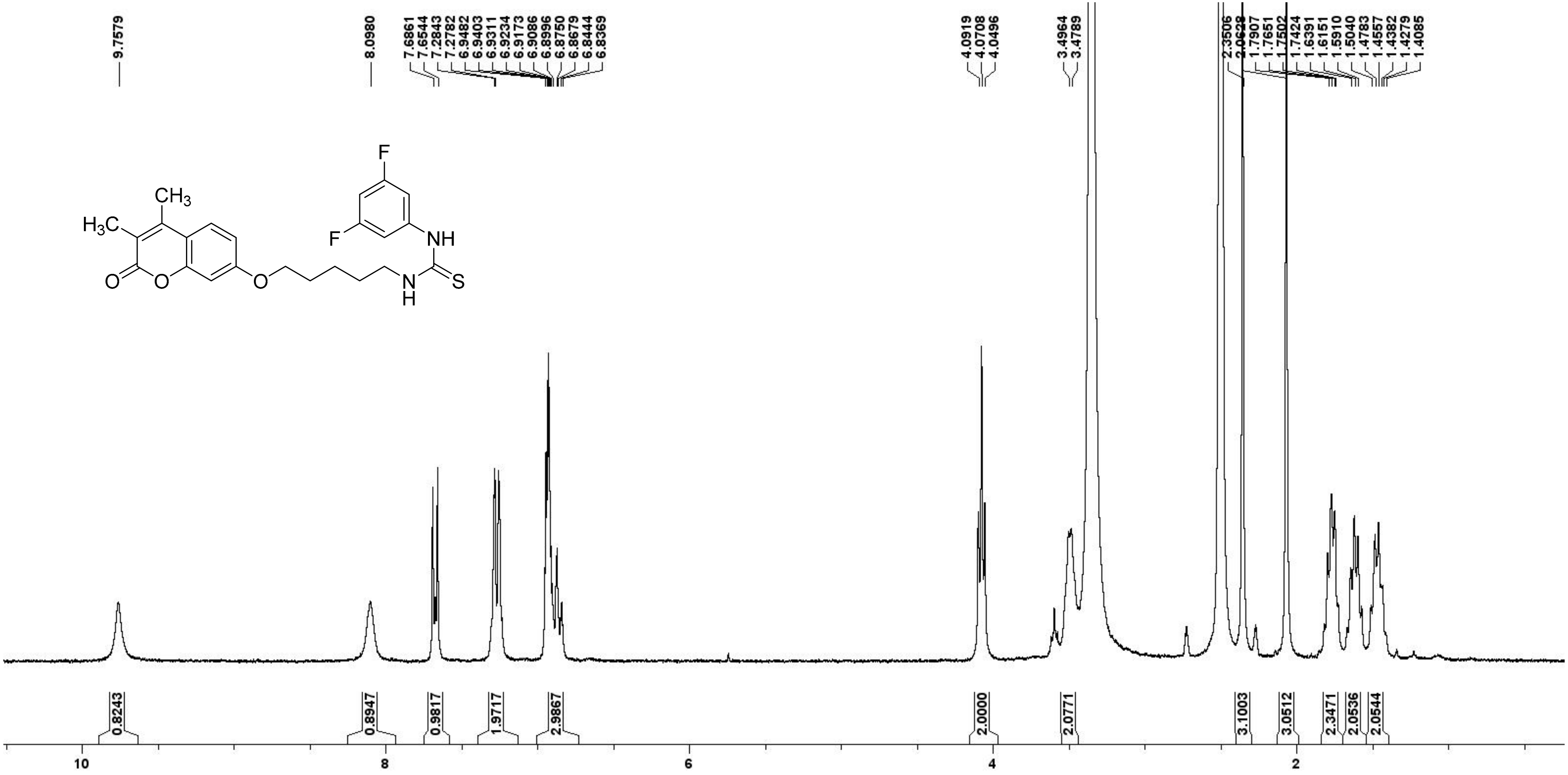

Figure S16. ^{1}H-NMR spectrum (300 MHz, DMSO-d_6) of **52**

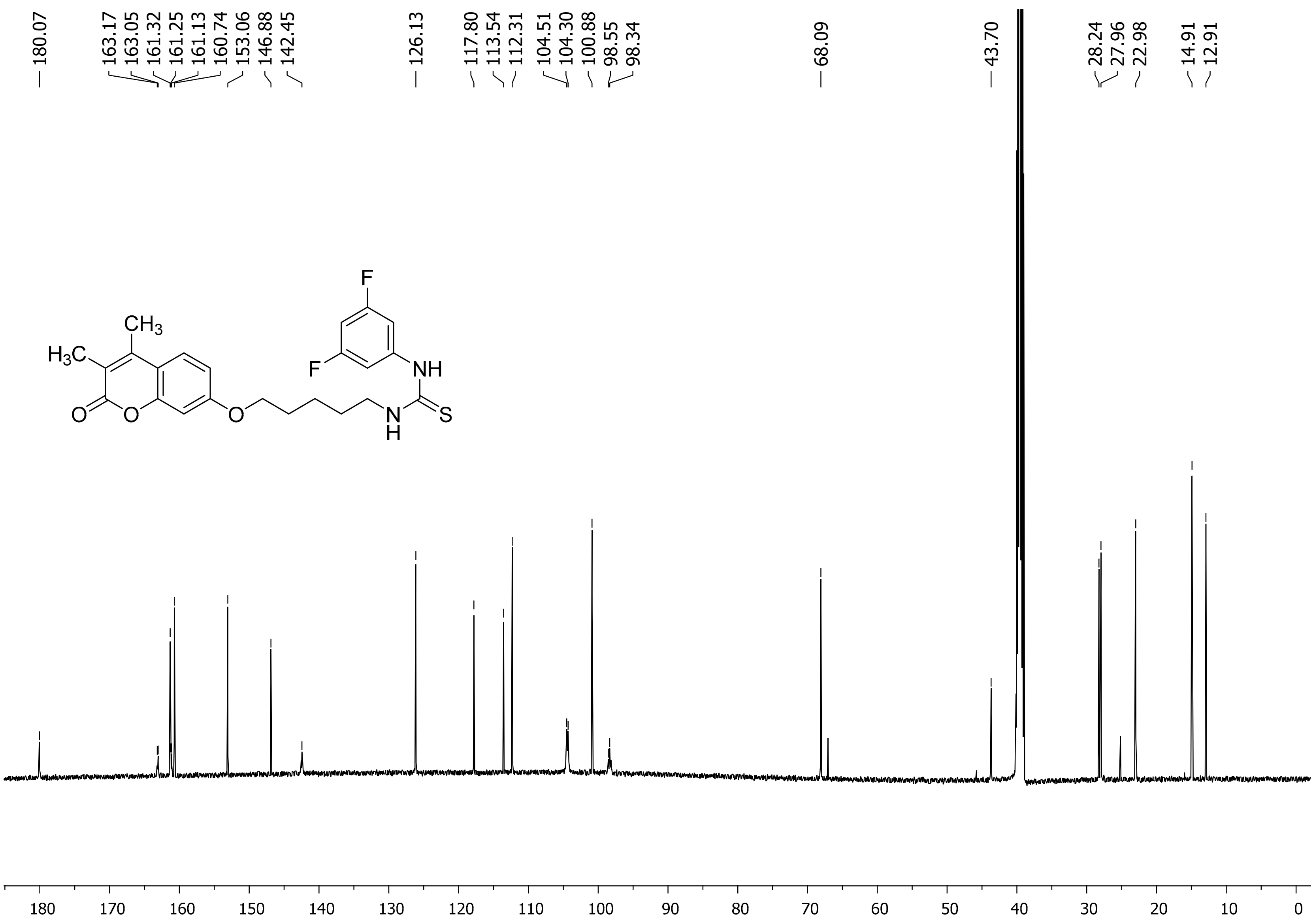

Figure S17. ^{13}C-NMR spectrum (125.7 MHz, DMSO-d_6) of **52**

INFORMACIÓN EXPERIMENTAL

Equipo: Elite	Fuente ionización: HESI	Modo: POSITIVO	Rango de masas: 60-900

230126_RC39S 01/26/23 11:36:28 RC39(S) PM=446 C23H24F2N2O3S

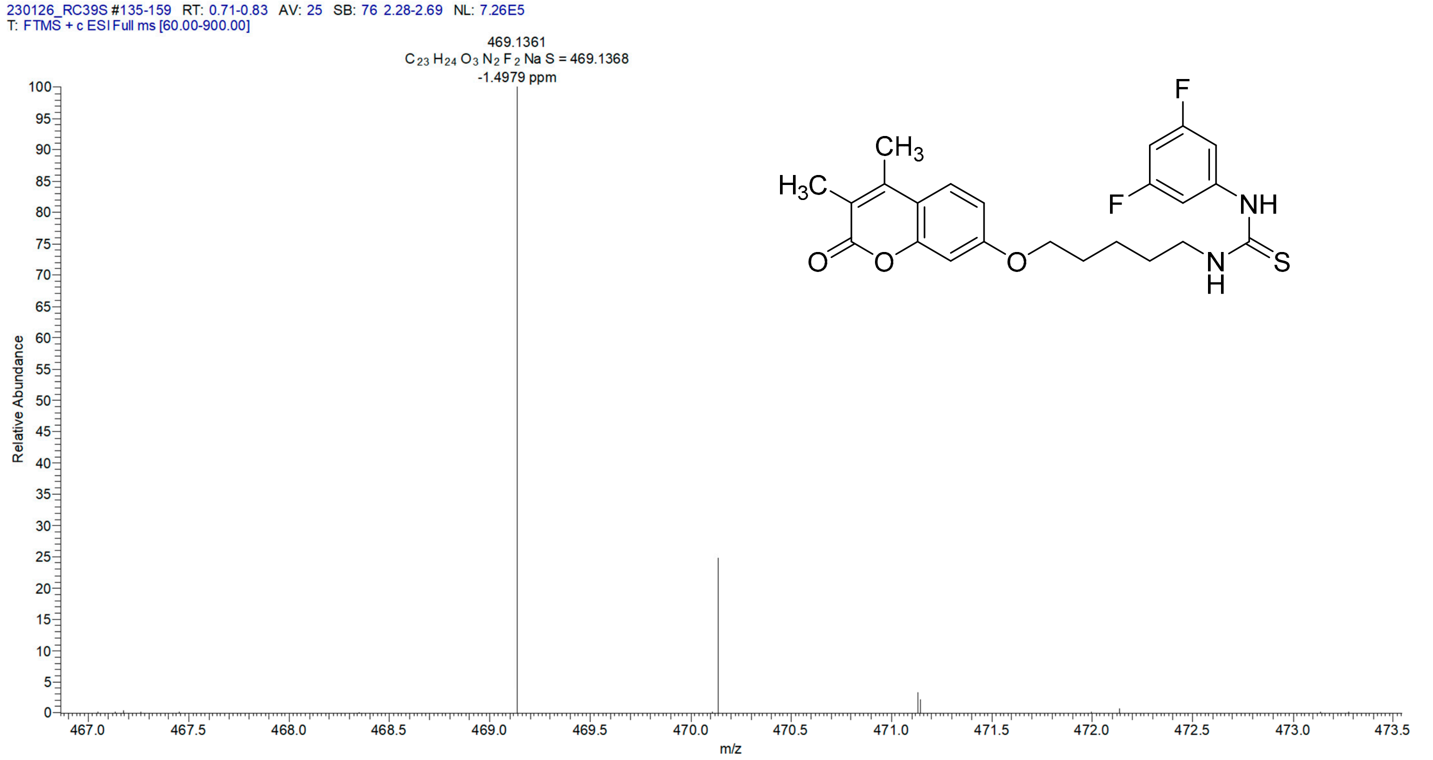

Figure S18. HRESI-MS of **52**

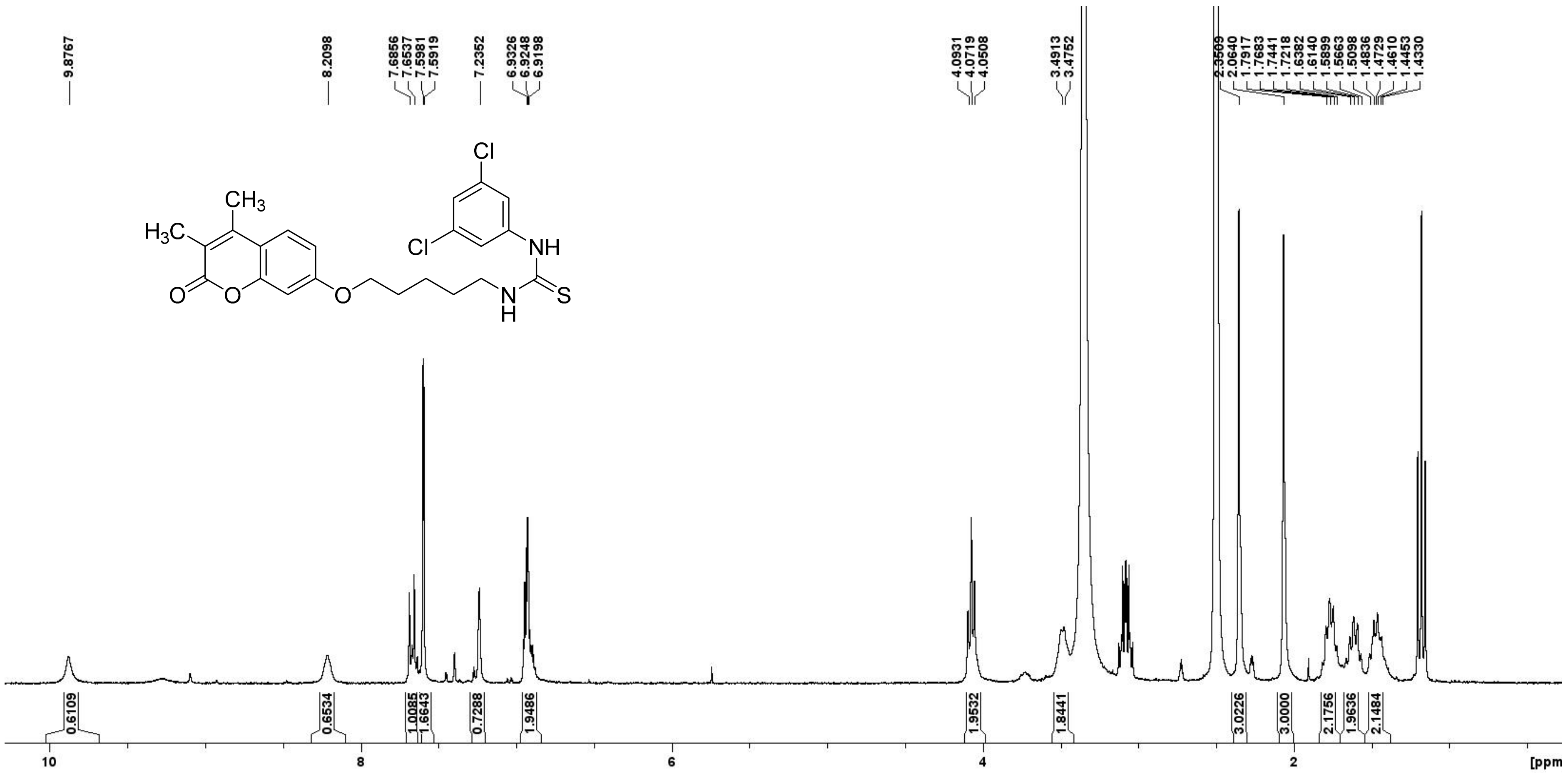

Figure S19. ^{1}H-NMR spectrum (300 MHz, DMSO-d_6) of **53**

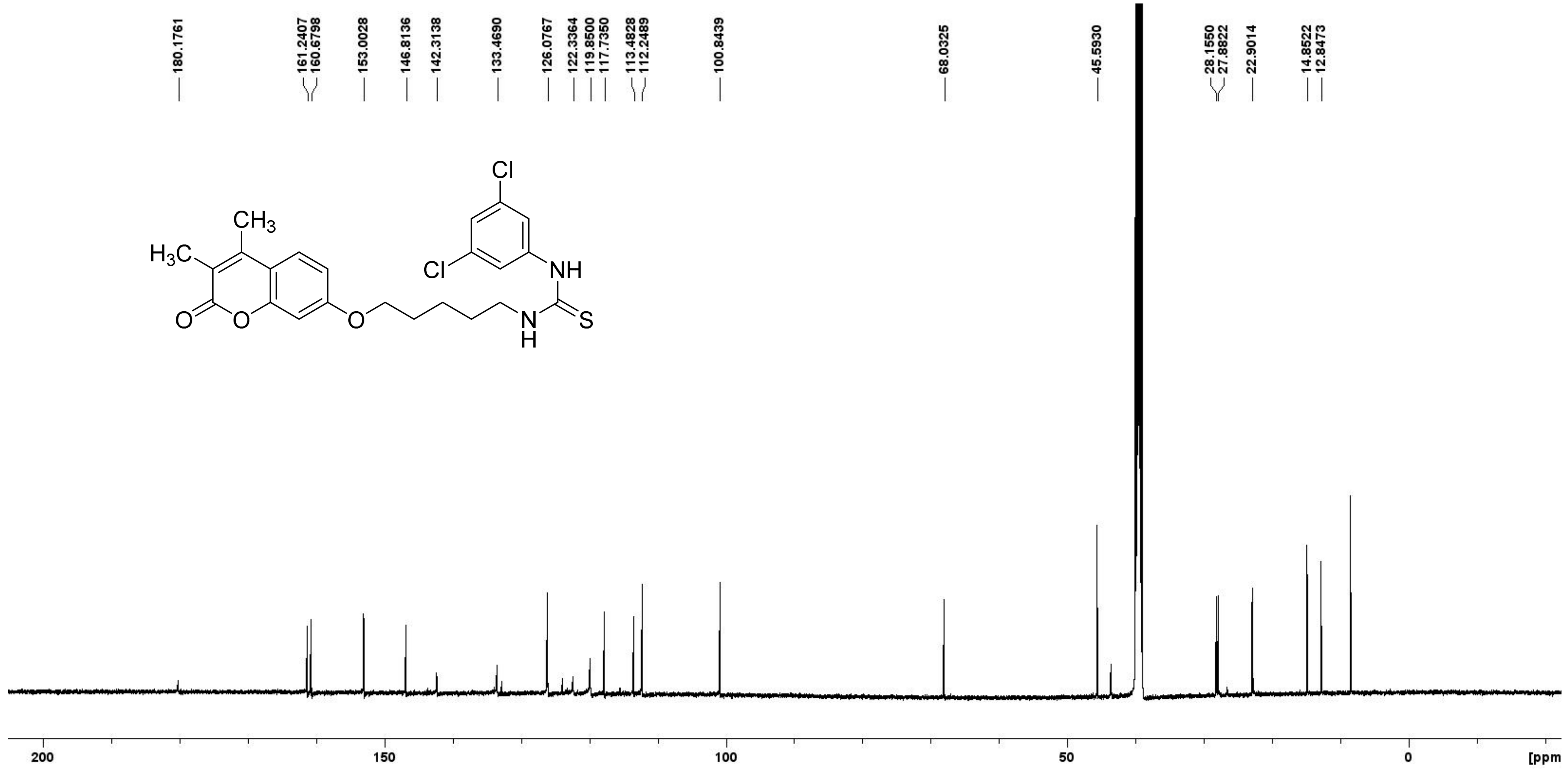

Figure S20. ^{13}C-NMR spectrum (125.7 MHz, DMSO-d_6) of **53**

Centro de Investigación, Tecnología e Innovación
Universidad de Sevilla
Avda. Reina Mercedes, nº 4-B, 41012 Sevilla, Spain

SERVICIO DE ESPECTROMETRIA DE MASAS

INFORMACIÓN EXPERIMENTAL

Equipo: Elite	Fuente ionización: HESI	Modo: POSITIVO	Rango de masas: 60-900

230126_RC48S 01/26/23 11:51:52 RC48(S) PM=478 C23H24Cl2N2O3S

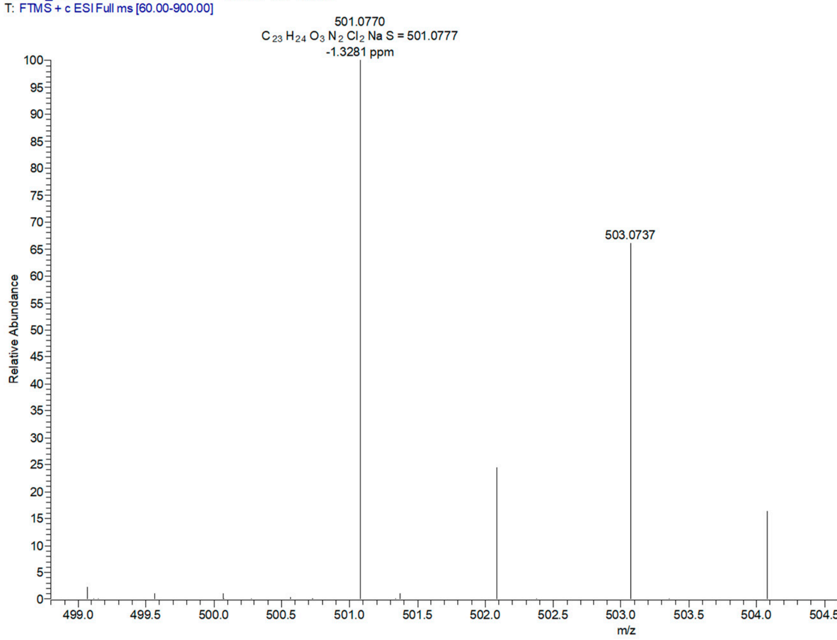

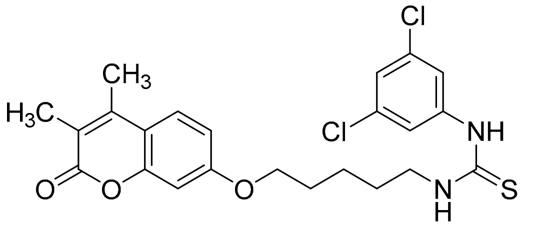

SGI Espectrometría de Masas
Tfno. 954559744; espectrometriademasas@us.es
Apdo. 1152, 41080 Sevilla, Spain
PNT07EPM0001-FT08
Rev 00
Fecha:25/05/2018

5

Figure S21. HRESI-MS of **53**

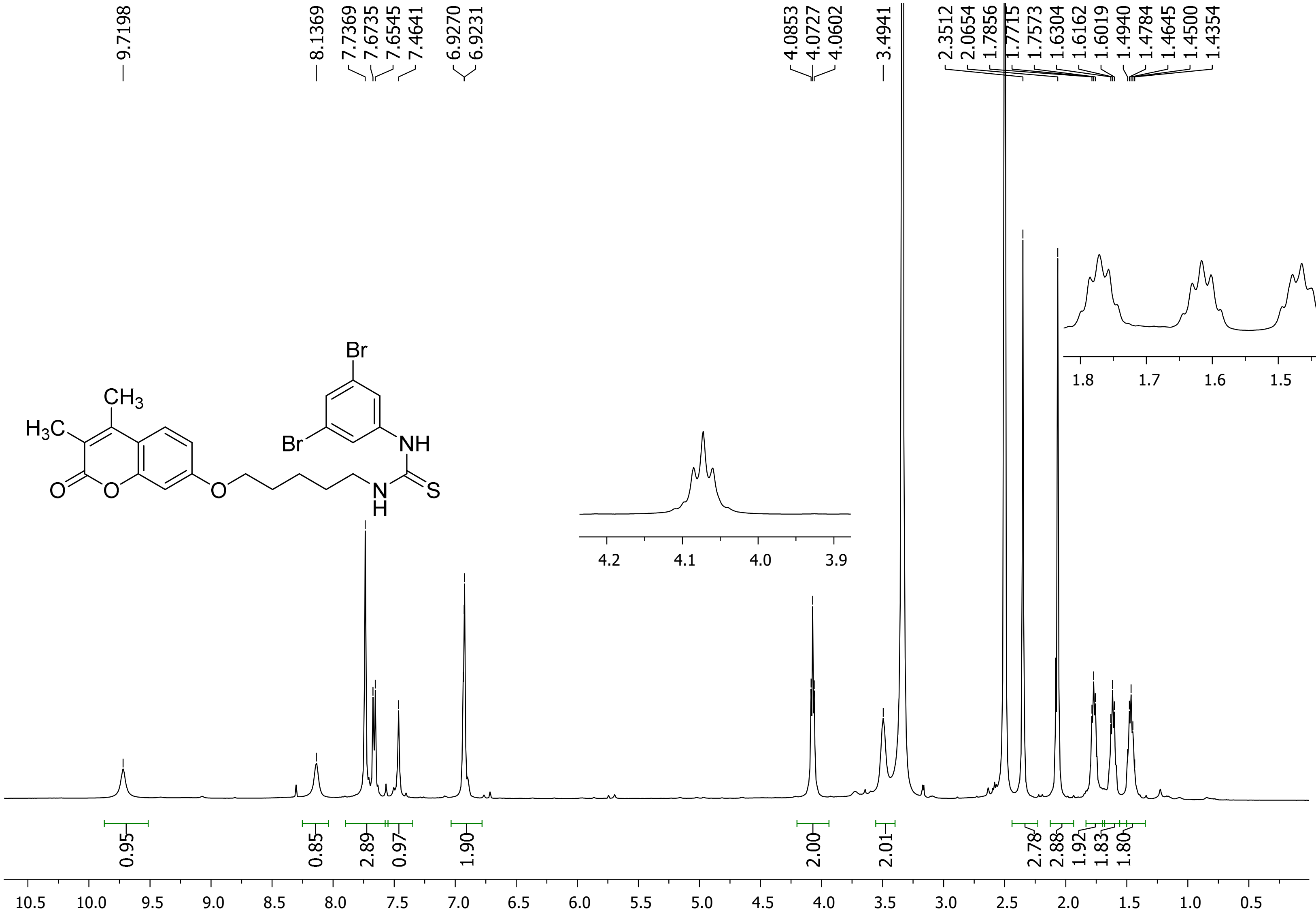

Figure S22. ^{1}H-NMR spectrum (500 MHz, DMSO-d_6) of **54**

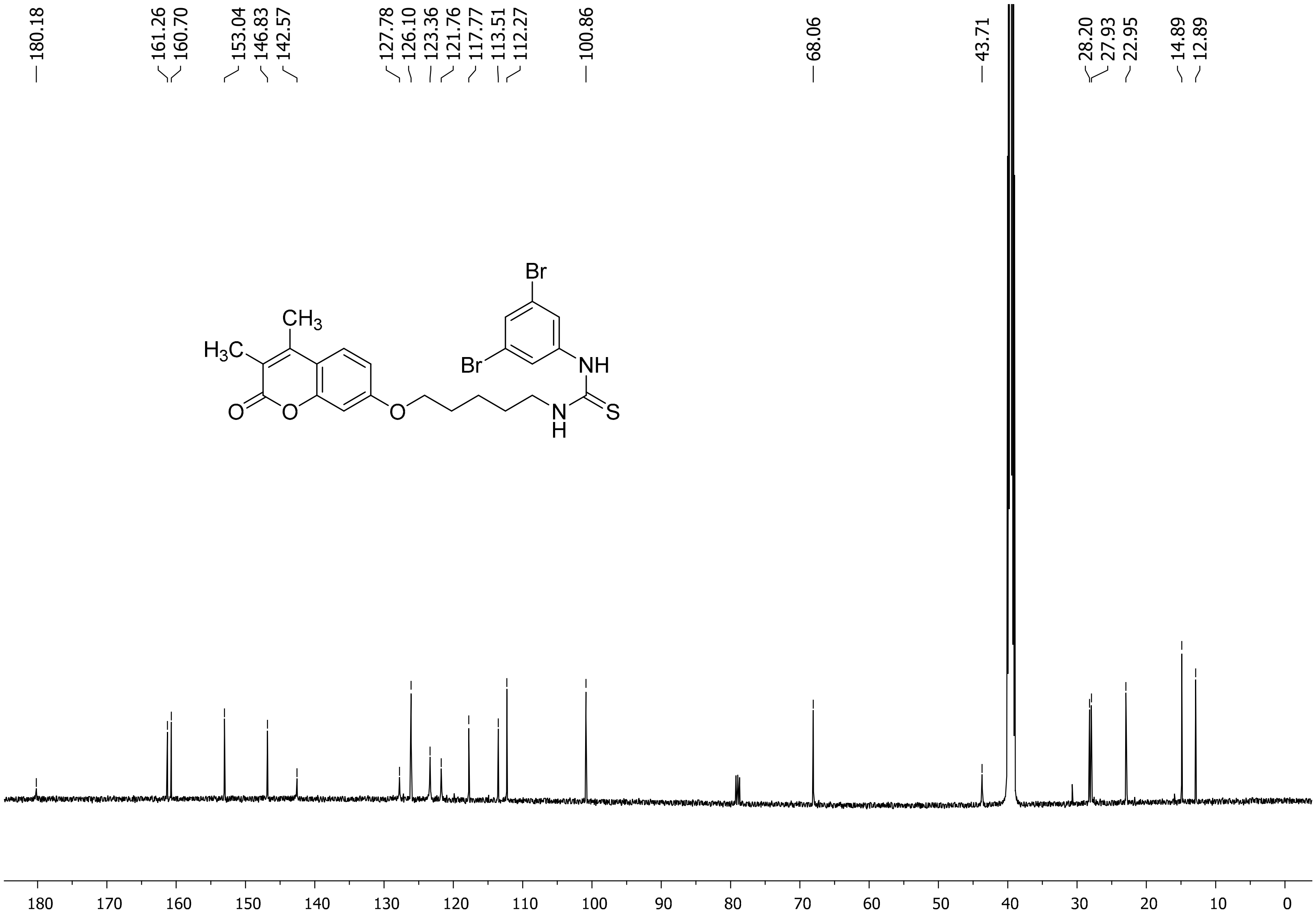

Figure S23. ^{13}C-NMR spectrum (125.7 MHz, DMSO-d_6) of **54**

INFORMACIÓN EXPERIMENTAL

Equipo: Elite	Fuente ionización: HESI	Modo: POSITIVO	Rango de masas: 60-900

230126_RC37S 01/26/23 11:31:35 RC37(S) PM=565 C23H24Br2N2O3S

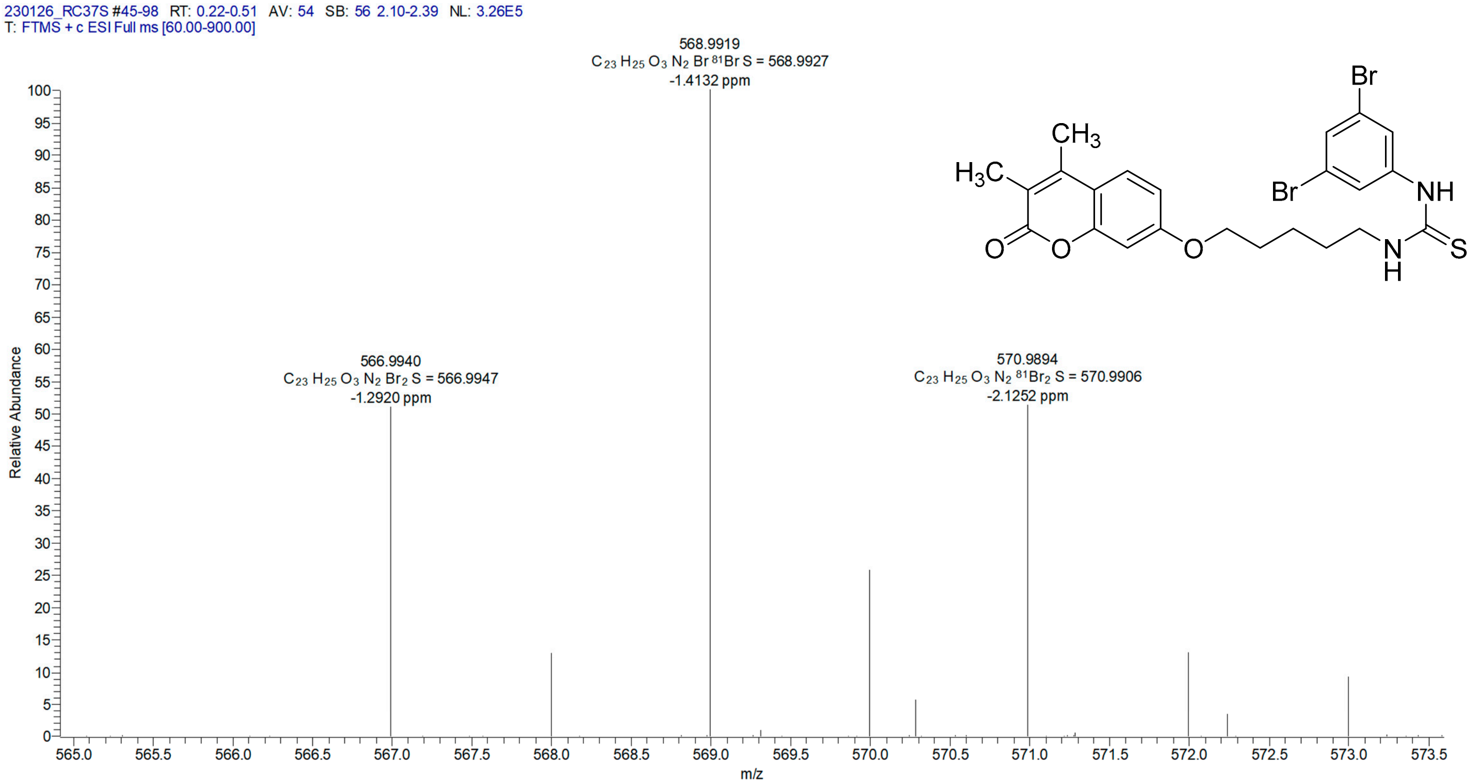

Figure S24. HRESI-MS of **54**

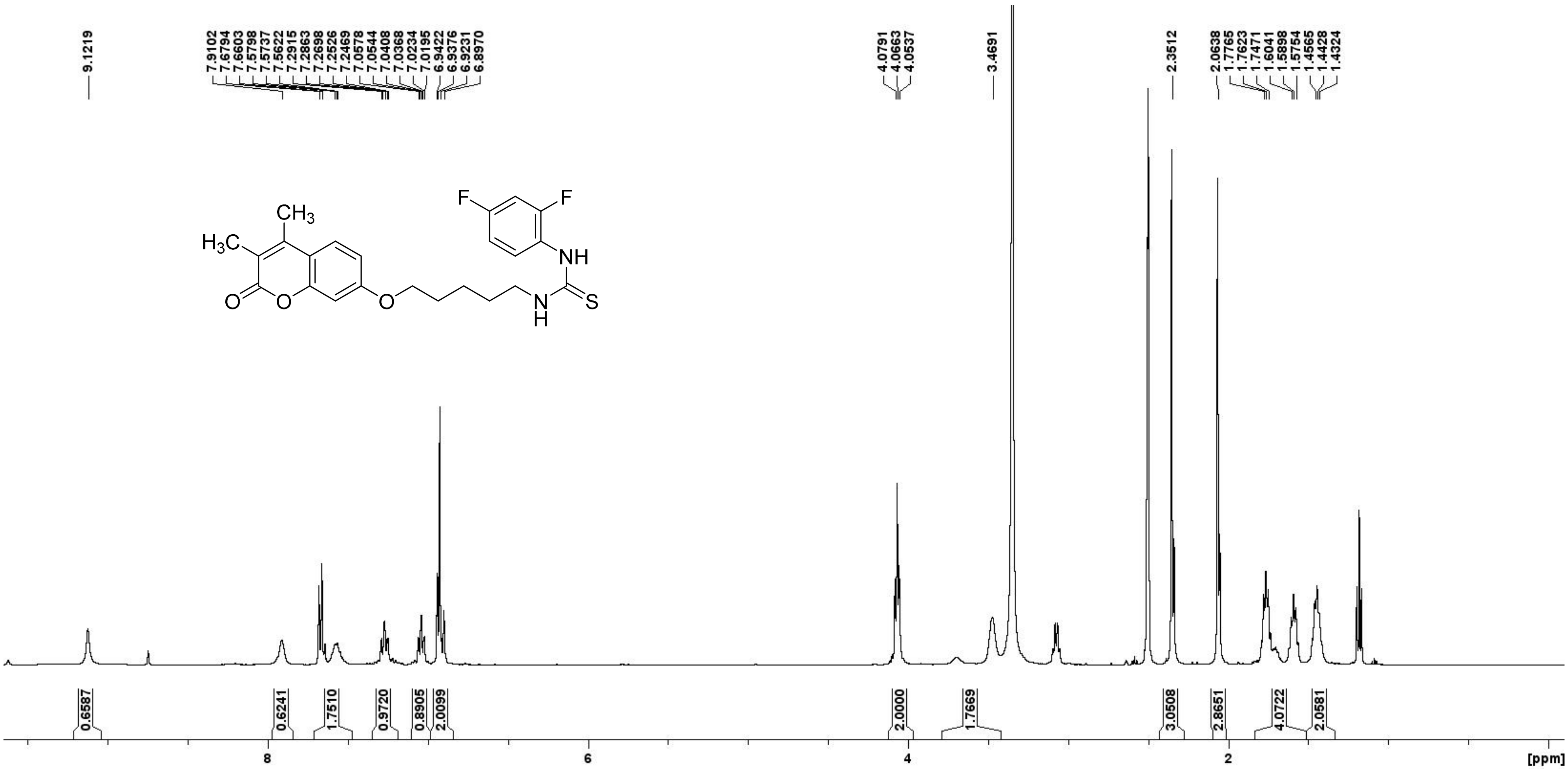

Figure S25. ^{1}H-NMR spectrum (500 MHz, DMSO-d_6) of **55**

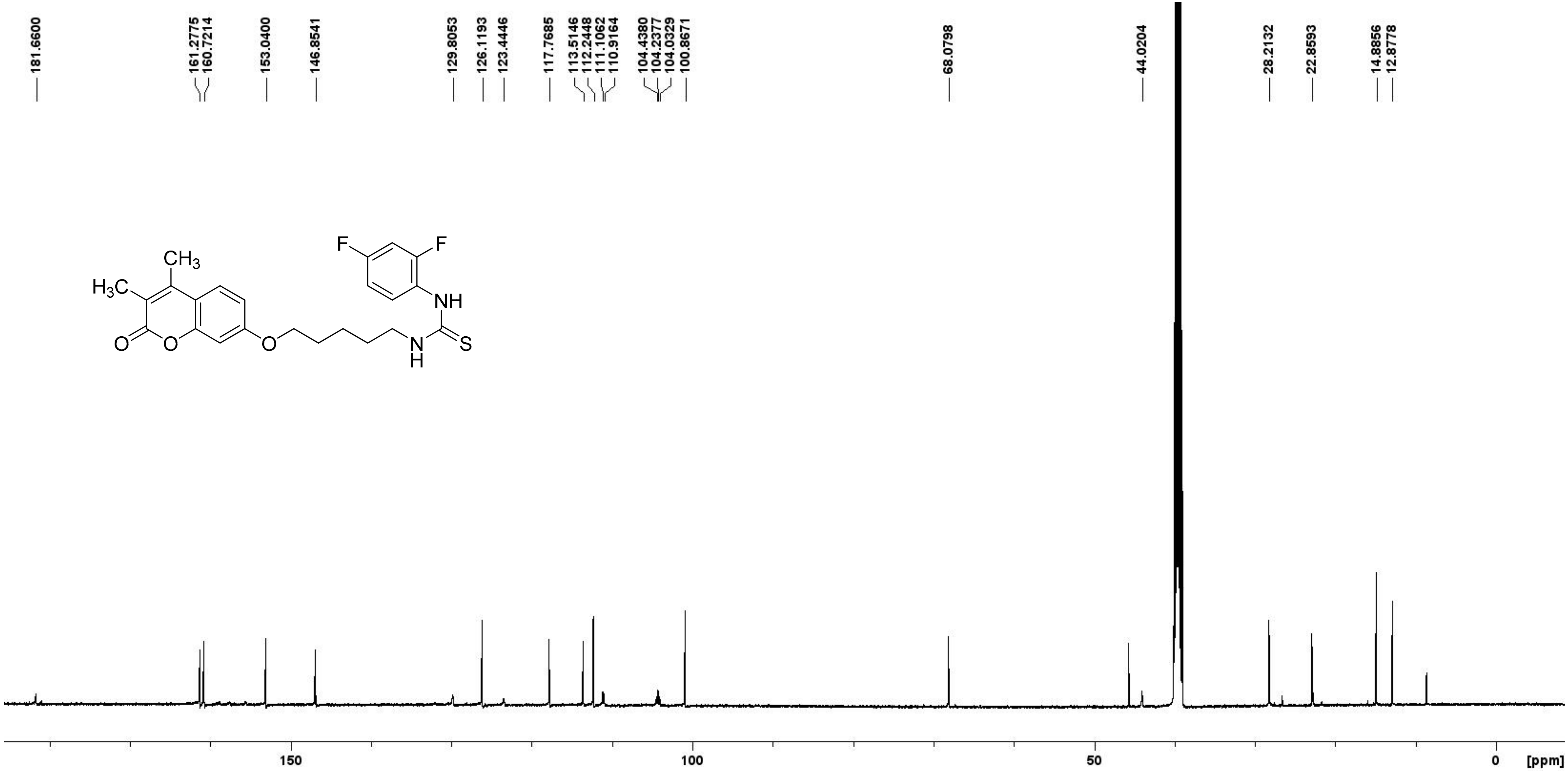

Figure S26. ^{13}C-NMR spectrum (125.7 MHz, DMSO-d_6) of **55**

INFORMACIÓN EXPERIMENTAL

Equipo: Elite	Fuente ionización: HESI	Modo: POSITIVO	Rango de masas: 60-900

230206_RC70S.raw 2/6/2023 1:49:10 PM RC70(S) PM=446 C23H24F2N2O3S

230206_RC70S #39-66 RT: 0.2-0.34 AV: 28 NL: 4.44E6
T: FTMS + c ESI Full ms [60.00-900.00]

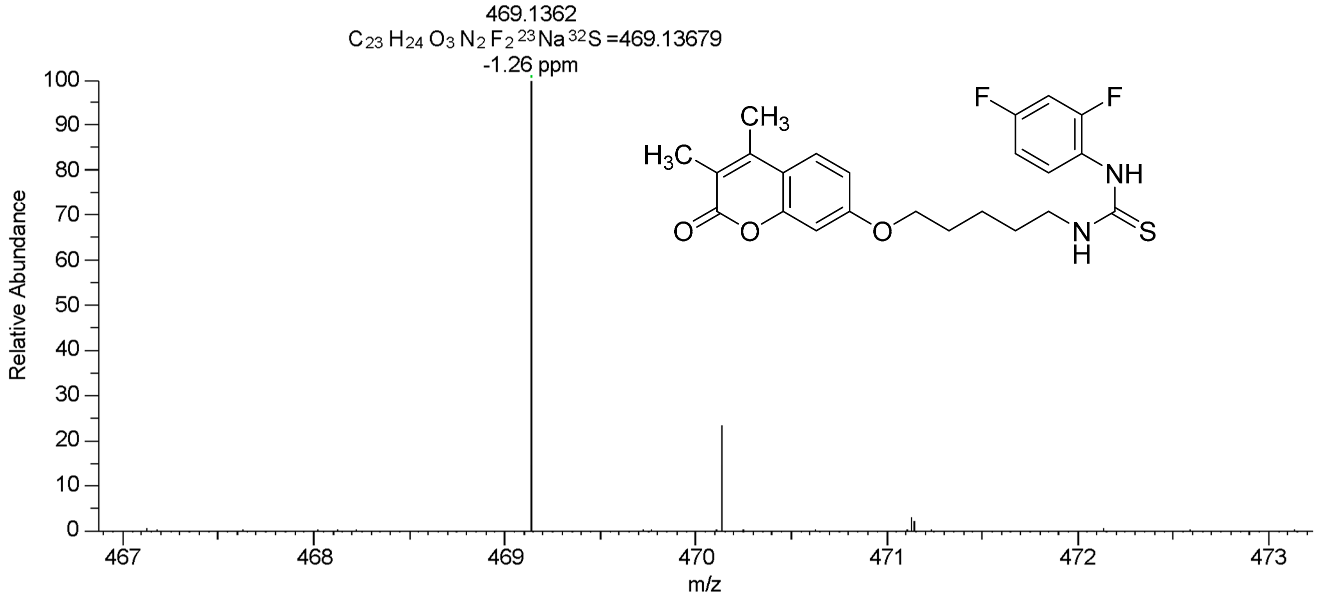

Figure S27. HRESI-MS of **55**

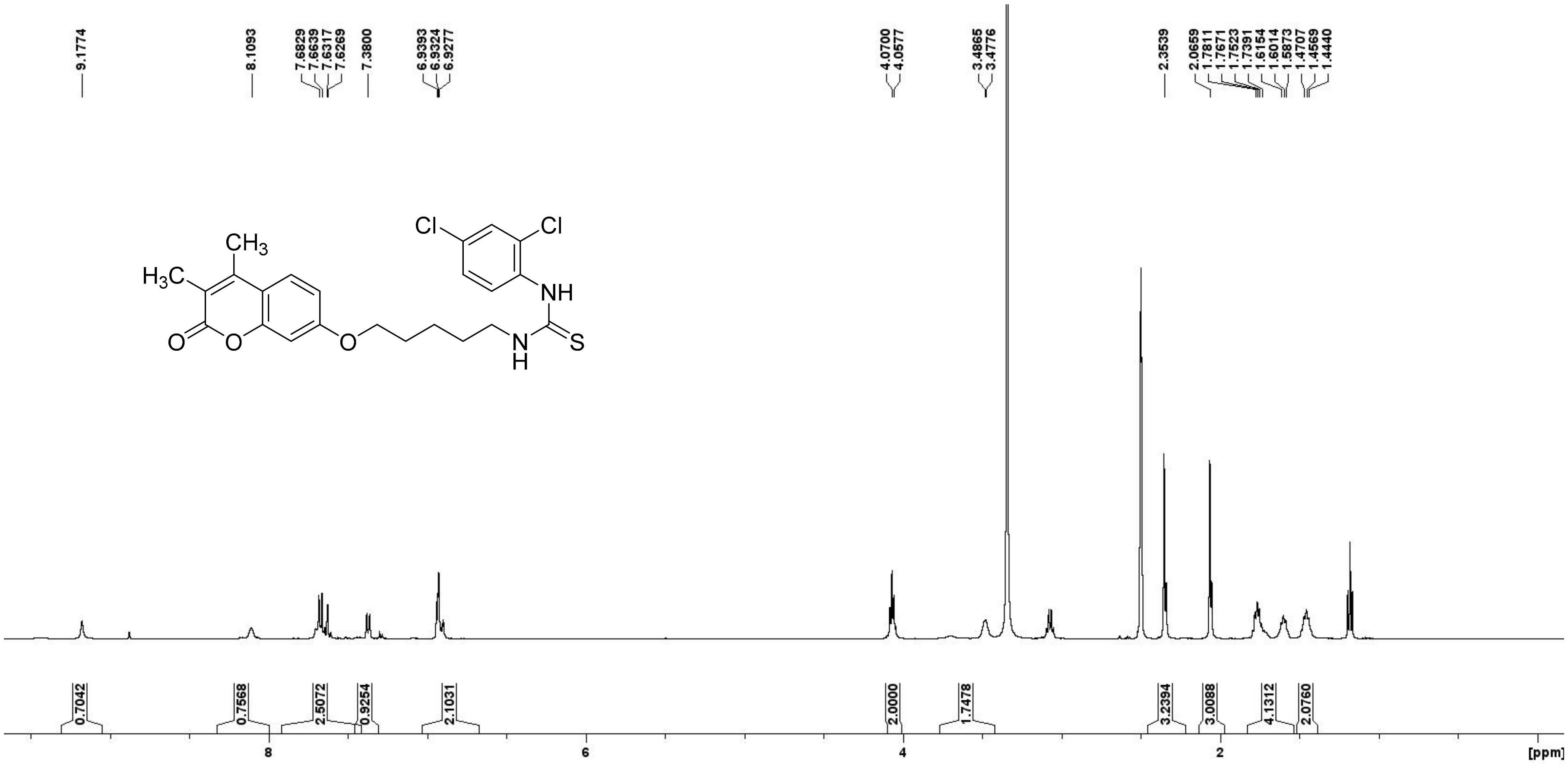

Figure S28. ^{1}H-NMR spectrum (500 MHz, DMSO-d_6) of **56**

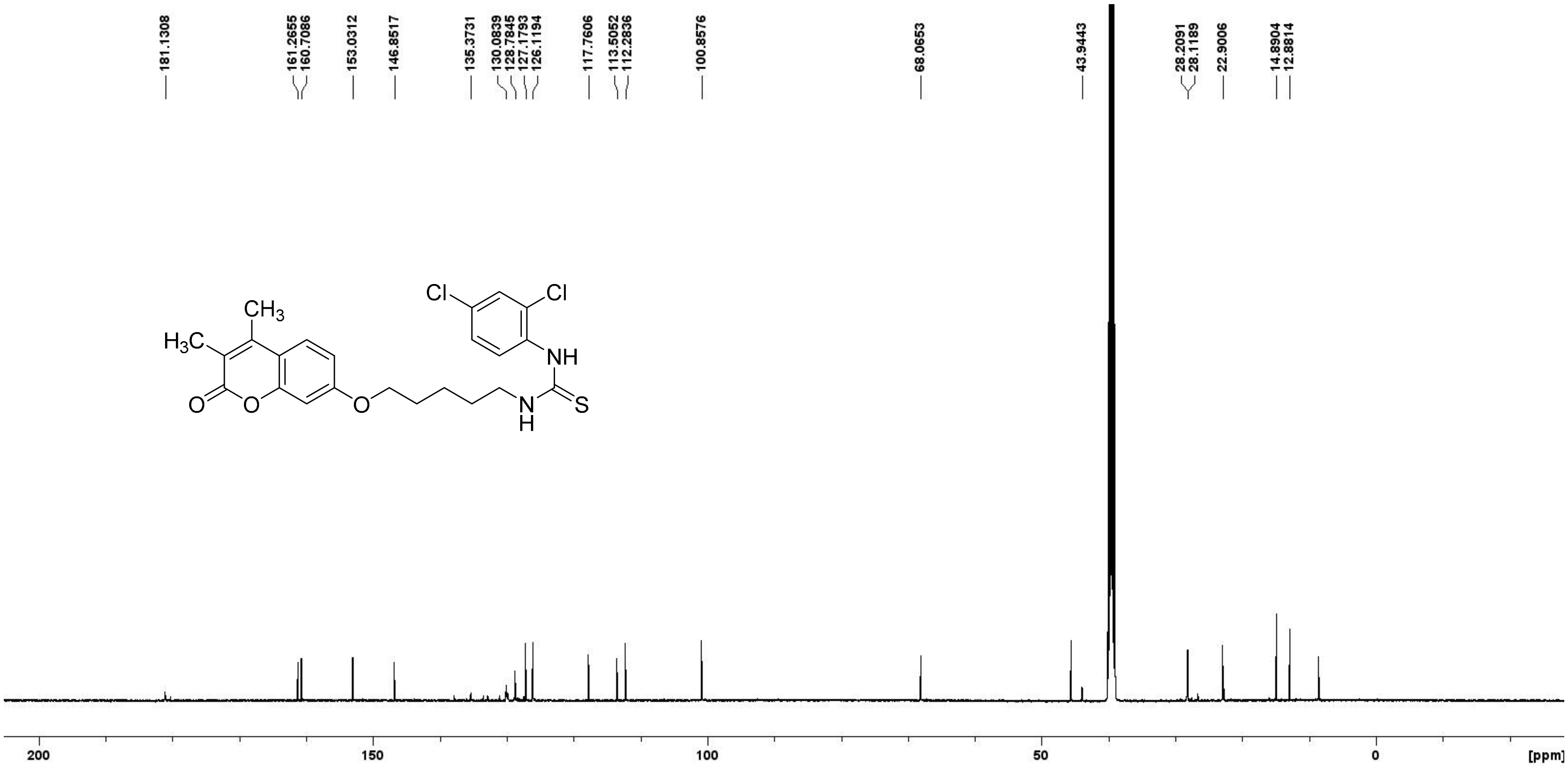

Figure S29. ^{13}C-NMR spectrum (125.7 MHz, DMSO-d_6) of **56**

INFORMACIÓN EXPERIMENTAL

Equipo: Elite	Fuente ionización: HESI	Modo: POSITIVO	Rango de masas: 60-900

230127_RC62S 01/27/23 14:05:28 RC62(S) PM=478 C23H24Cl2N2O3S

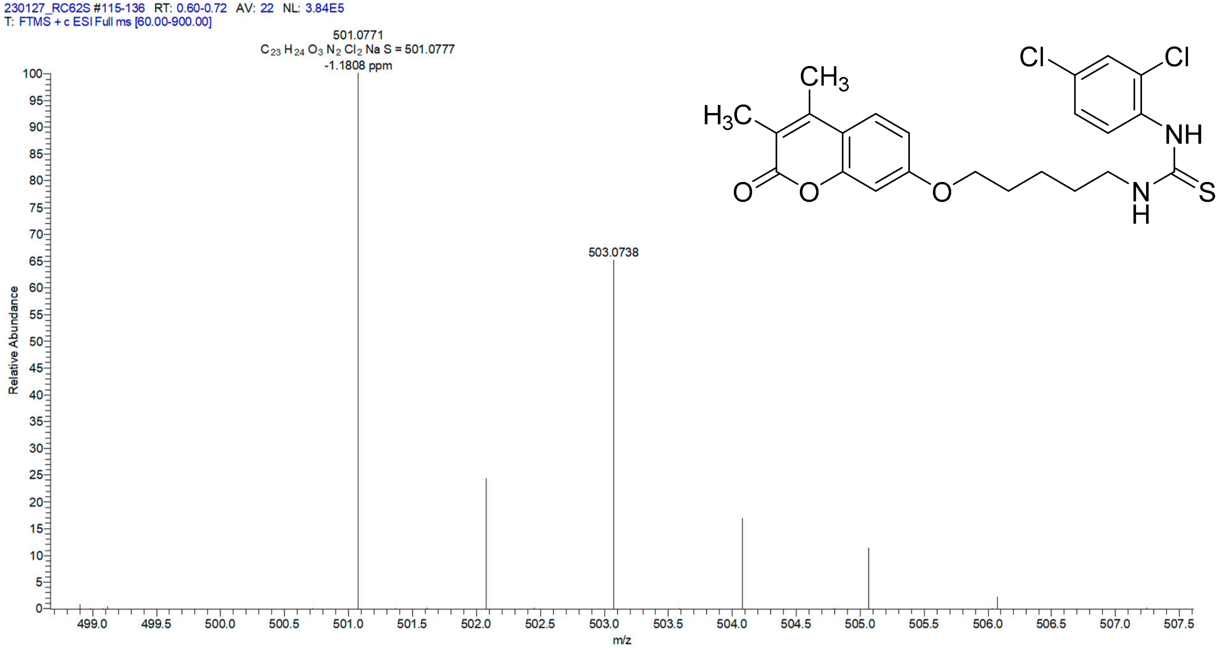

Figure S30. HRESI-MS of **56**

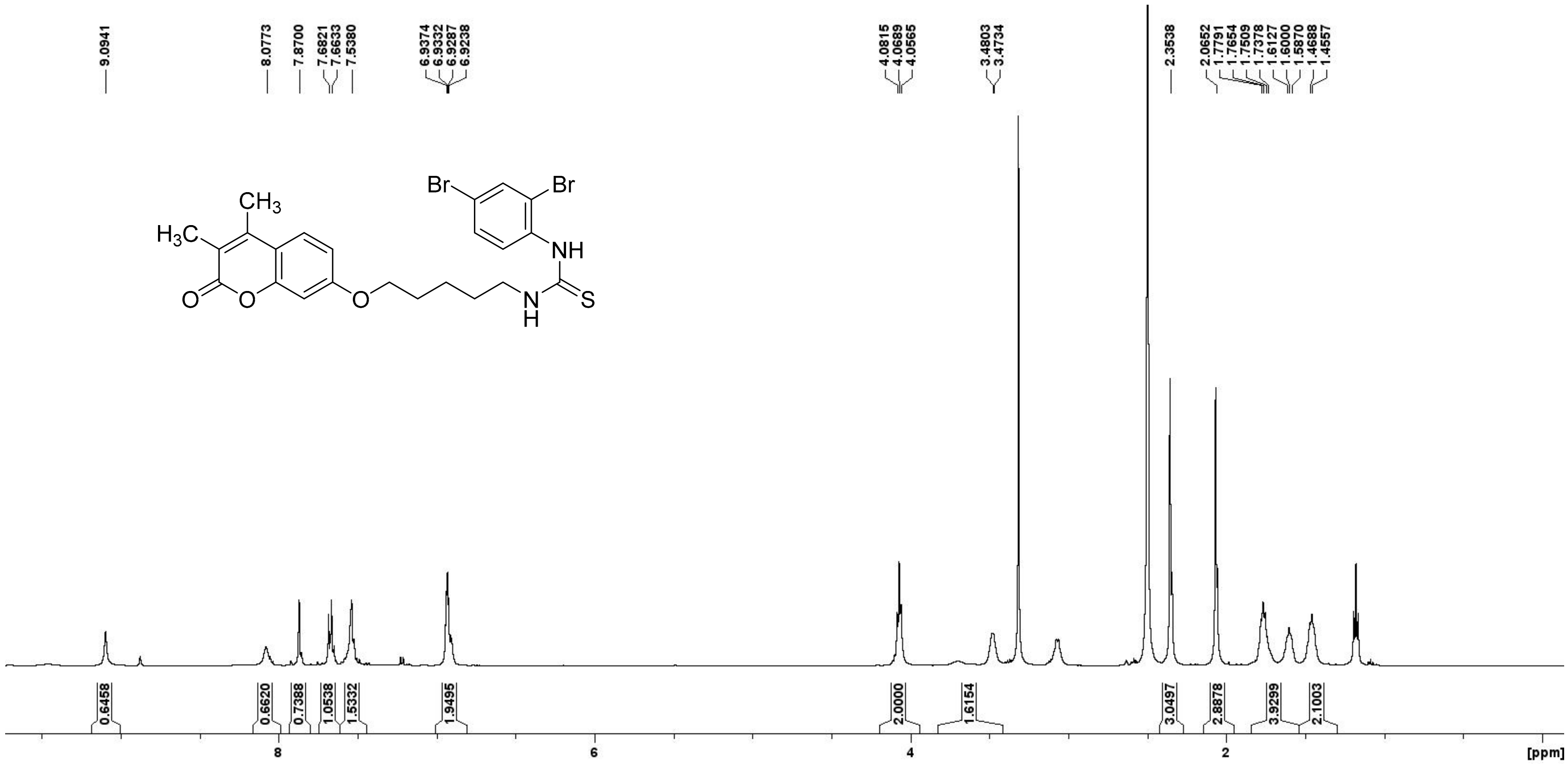

Figure S31. ^{1}H-NMR spectrum (500 MHz, DMSO-d_6) of **57**

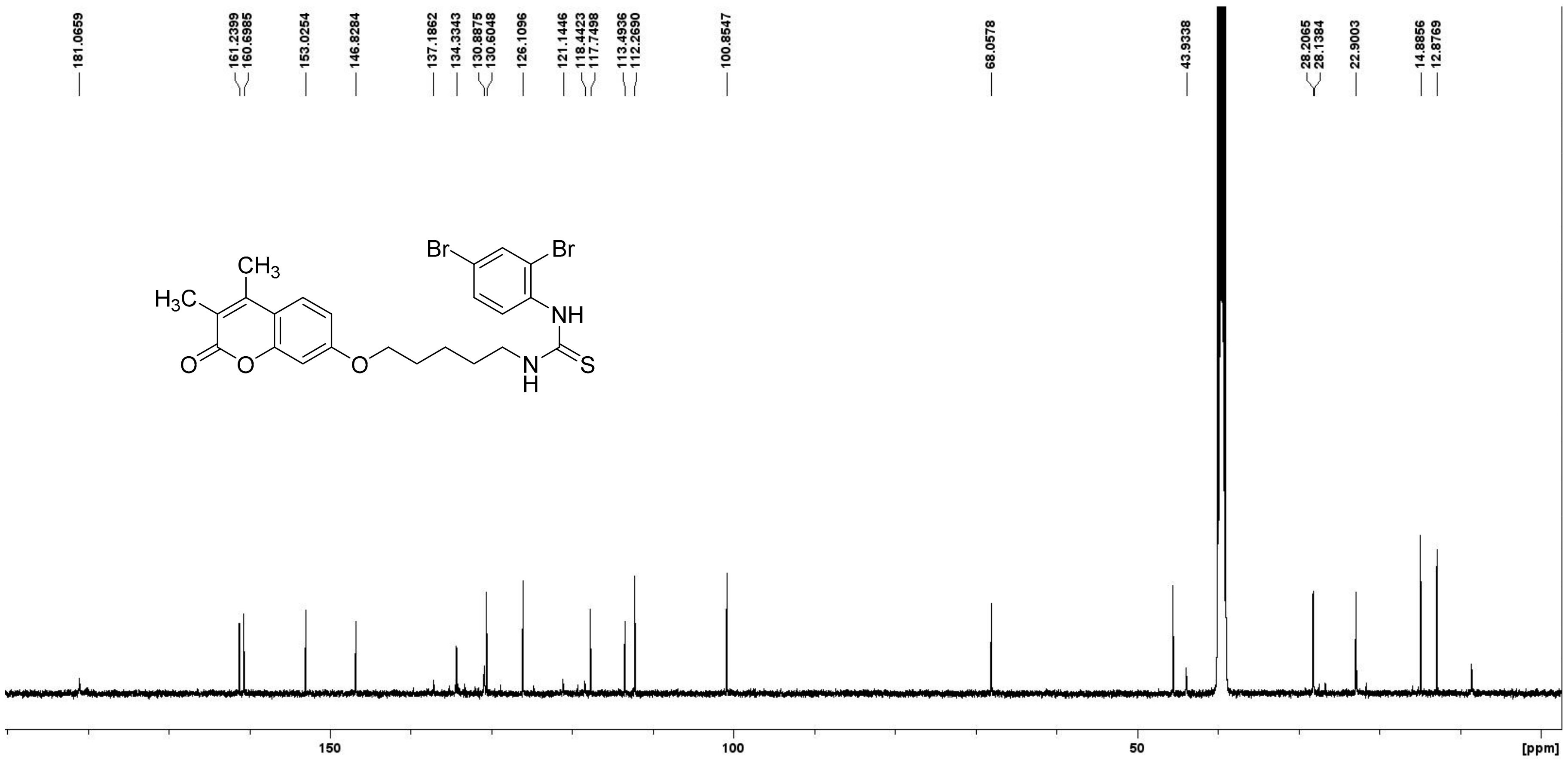

Figure S32. ^{13}C-NMR spectrum (125.7 MHz, DMSO-d_6) of **57**

Centro de Investigación, Tecnología e Innovación
Universidad de Sevilla
Avda. Reina Mercedes, nº 4-B, 41012 Sevilla, Spain

SERVICIO DE ESPECTROMETRIA DE MASAS

INFORMACIÓN EXPERIMENTAL

Equipo: Elite	Fuente ionización: HESI	Modo: POSITIVO	Rango de masas: 60-900

230201_RC67S 02/01/23 10:17:59 RC67(S) PM=565 C23H24Br2FN2O3S

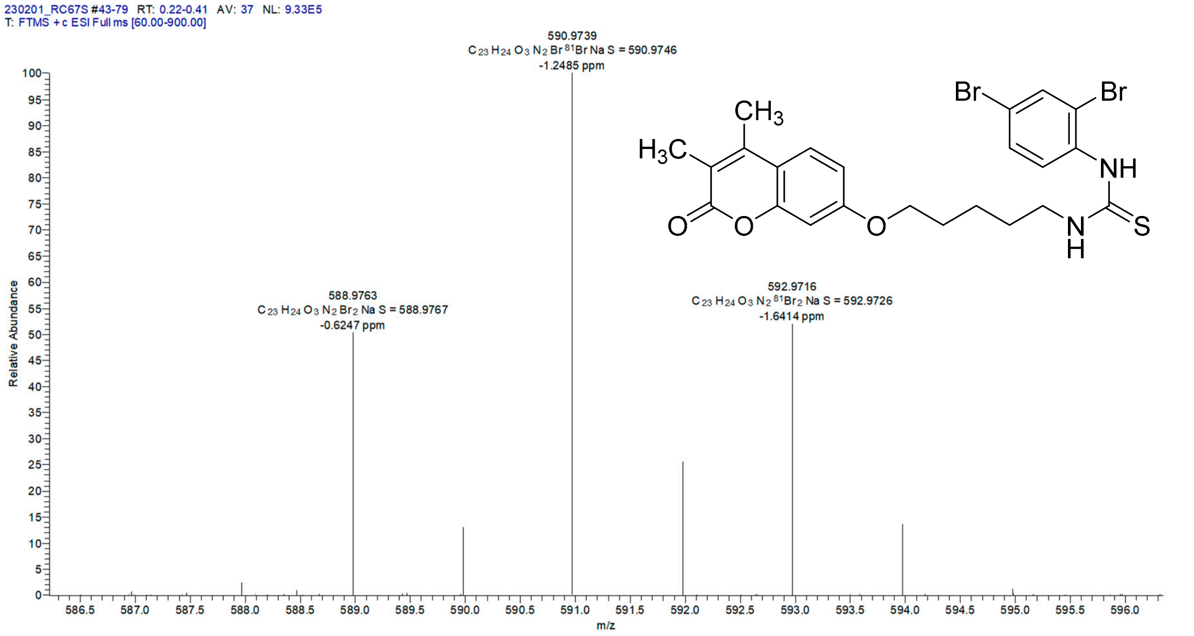

SGI Espectrometría de Masas
Tfno. 954559744; espectrometriademasas@us.es
Apdo. 1152, 41080 Sevilla, Spain
PNT07EPM0001-FT08
Rev 00
Fecha:25/05/2018

4

Figure S33. HRESI-MS of **57**

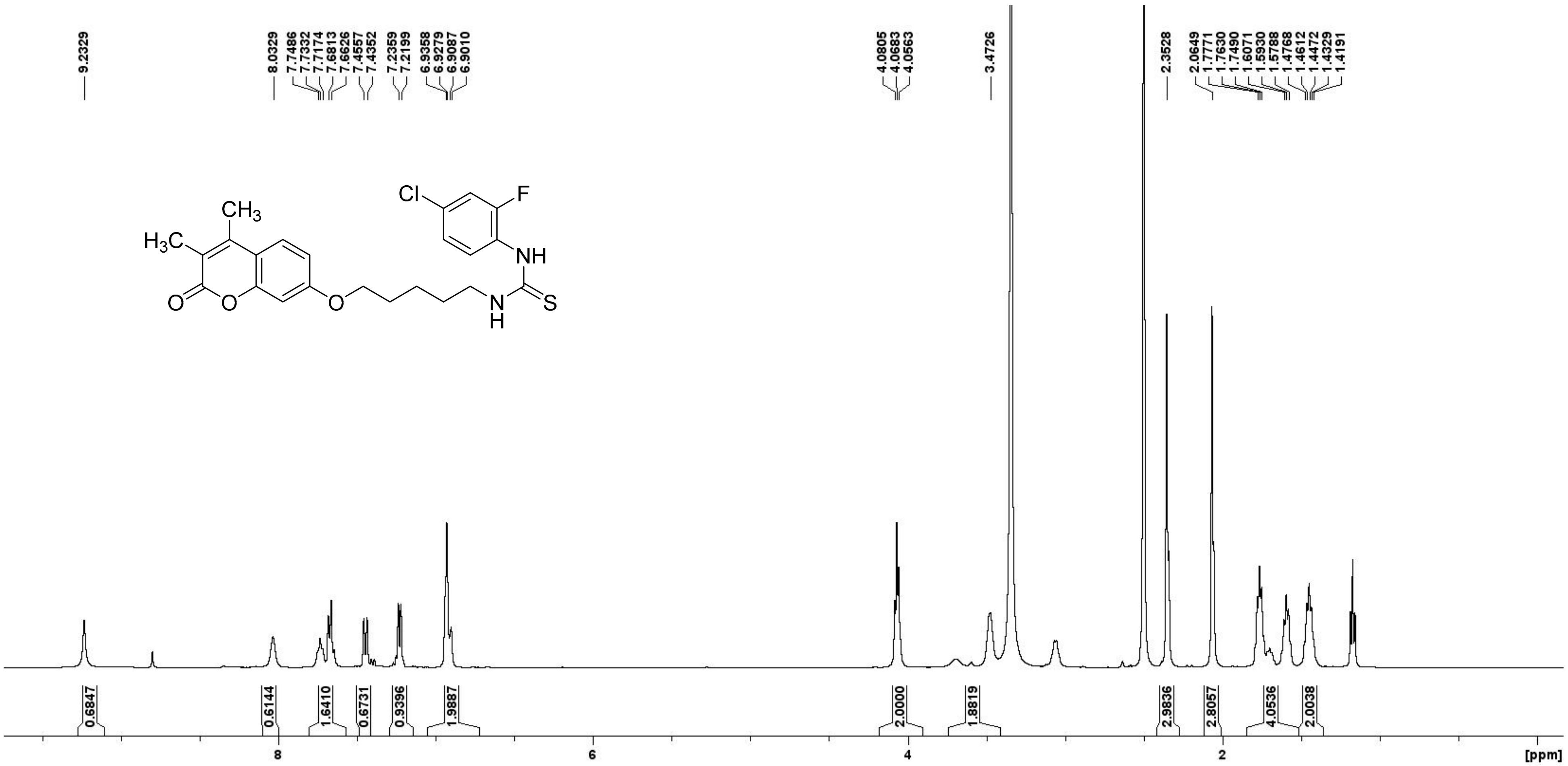

Figure S34. ^{1}H-NMR spectrum (500 MHz, DMSO-d_6) of **58**

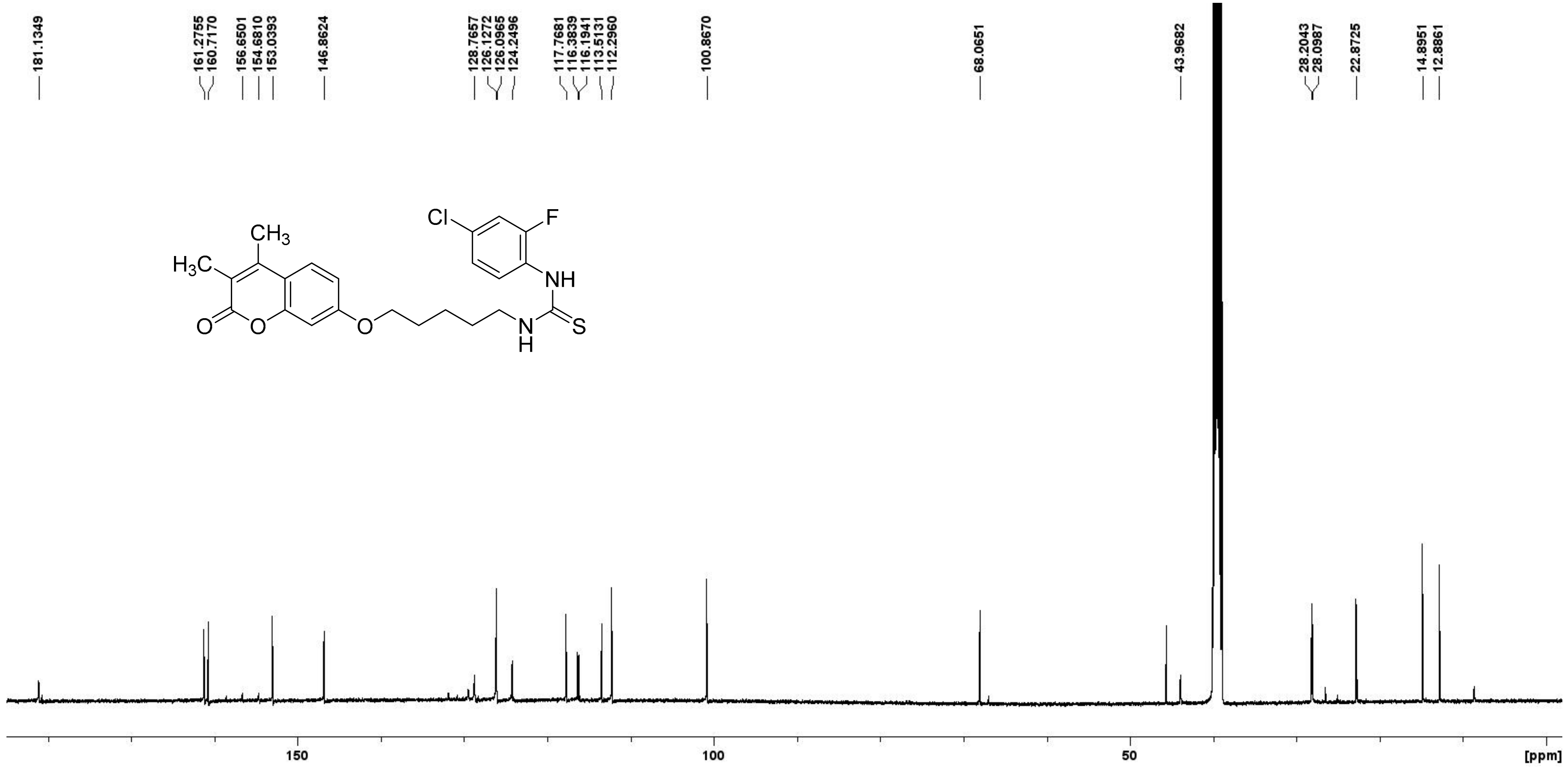

Figure S35. ^{13}C-NMR spectrum (125.7 MHz, DMSO-d_6) of **58**

Centro de Investigación, Tecnología e Innovación
Universidad de Sevilla
Avda. Reina Mercedes, nº 4-B, 41012 Sevilla, Spain

SERVICIO DE ESPECTROMETRIA DE MASAS

INFORMACIÓN EXPERIMENTAL

Equipo: Elite	Fuente ionización: HESI	Modo: POSITIVO	Rango de masas: 60-900

230201_RC66S 02/01/23 10:07:43 RC66(S) PM=462 C23H24ClFN2O3S

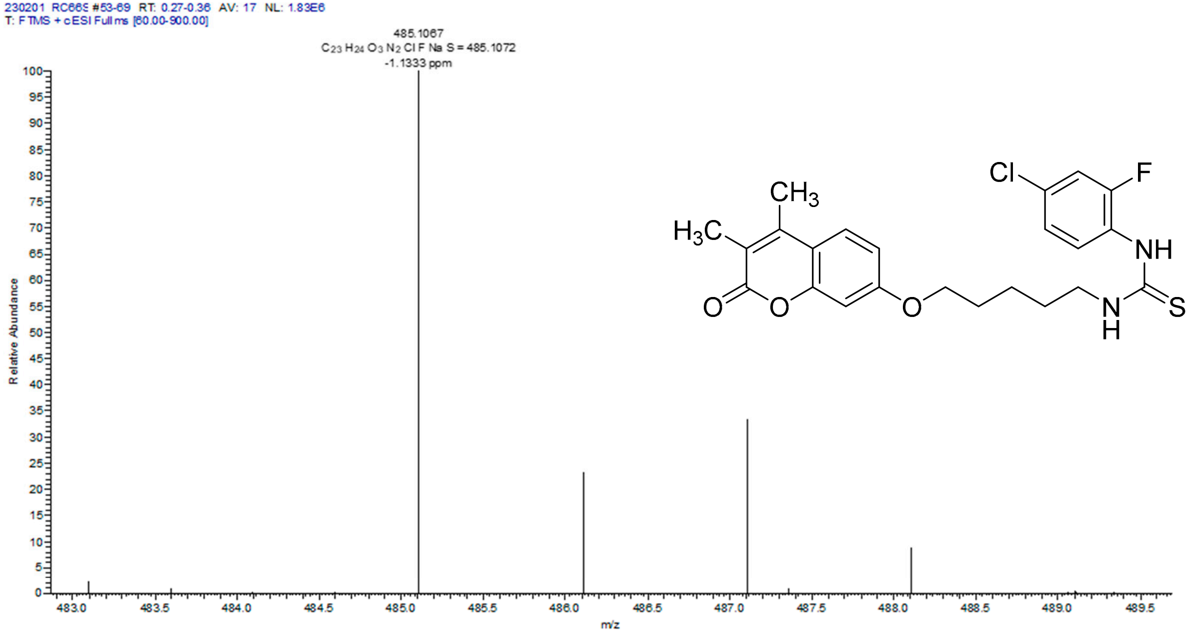

SGI Espectrometría de Masas
Tfno. 954559744; espectrometriademasas@us.es
Apdo. 1152, 41080 Sevilla. Spain
PNT07EPM0001-FT08
Rev 00
Fecha:25/05/2018

2

Figure S36. HRESI-MS of **58**

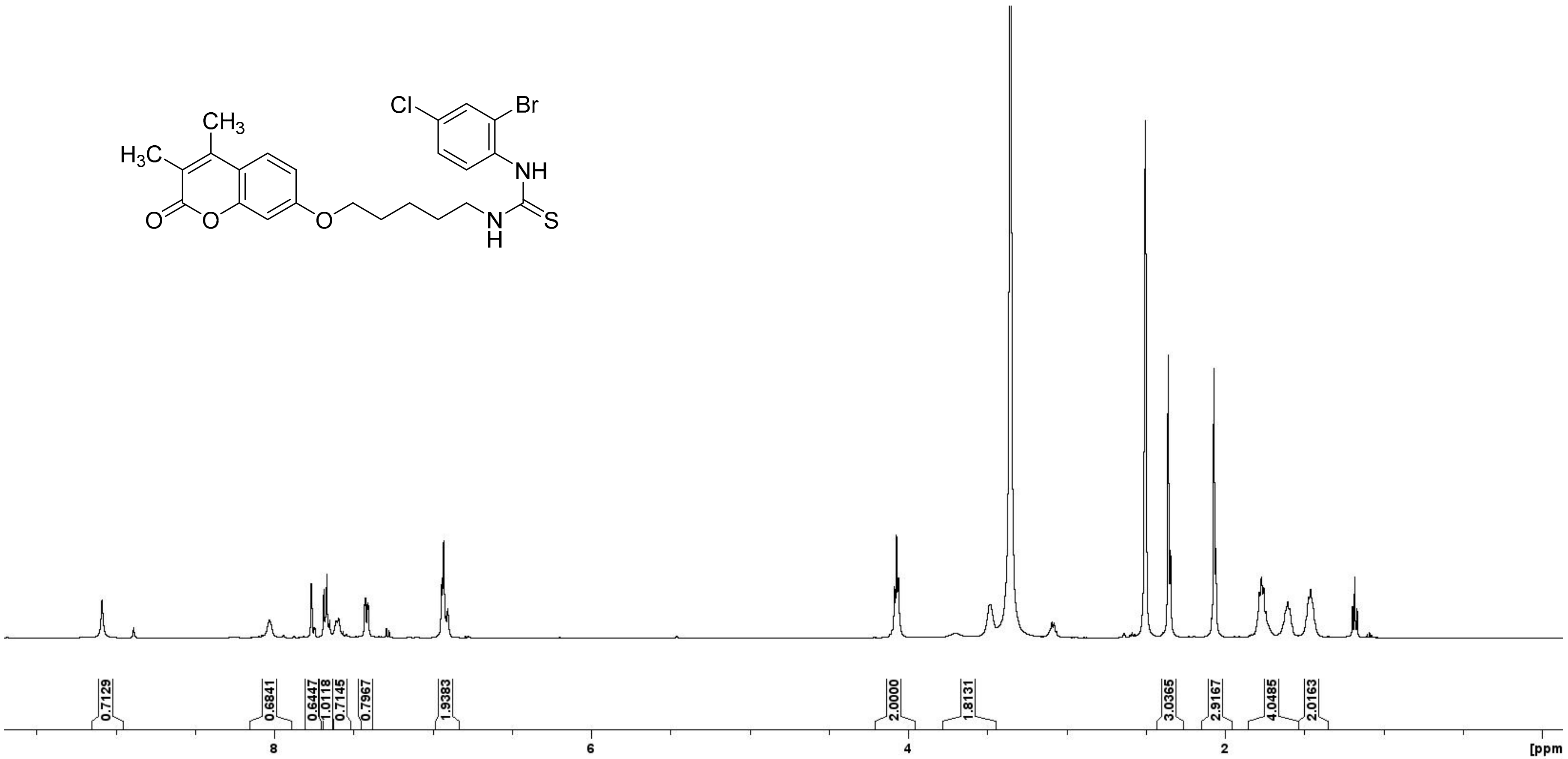

Figure S37. ^{1}H-NMR spectrum (500 MHz, DMSO-d_6) of **59**

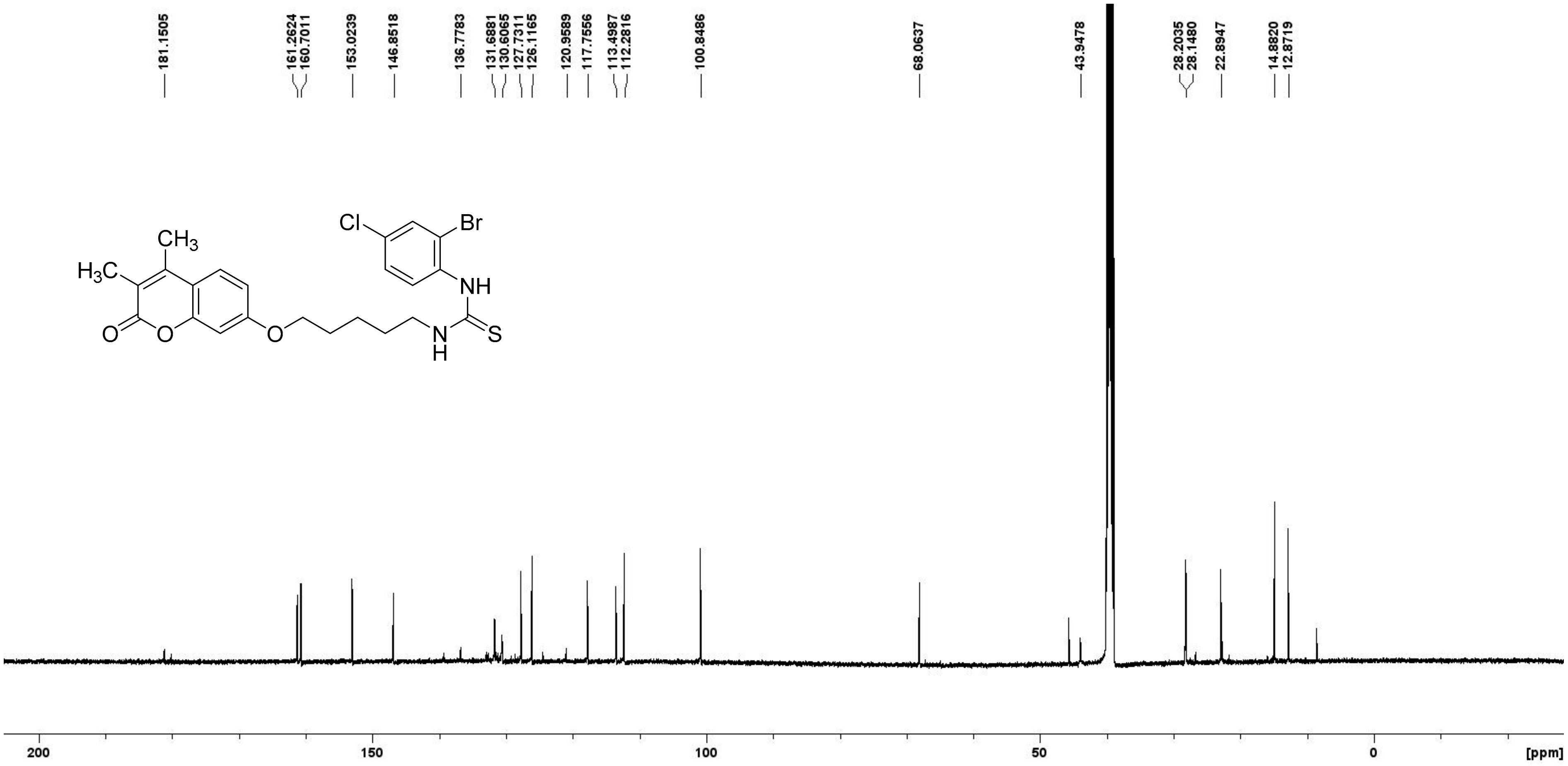

Figure S38. ^{13}C-NMR spectrum (125.7 MHz, DMSO-d_6) of **59**

Centro de Investigación, Tecnología e Innovación
Universidad de Sevilla
Avda. Reina Mercedes, nº 4-B, 41012 Sevilla, Spain

SERVICIO DE ESPECTROMETRIA DE MASAS

INFORMACIÓN EXPERIMENTAL

Equipo: Elite	Fuente ionización: HESI	Modo: POSITIVO	Rango de masas: 60-900

230201_RC64S 02/01/23 10:02:37 RC64(S) PM=522 C23H24BrClN2O3S

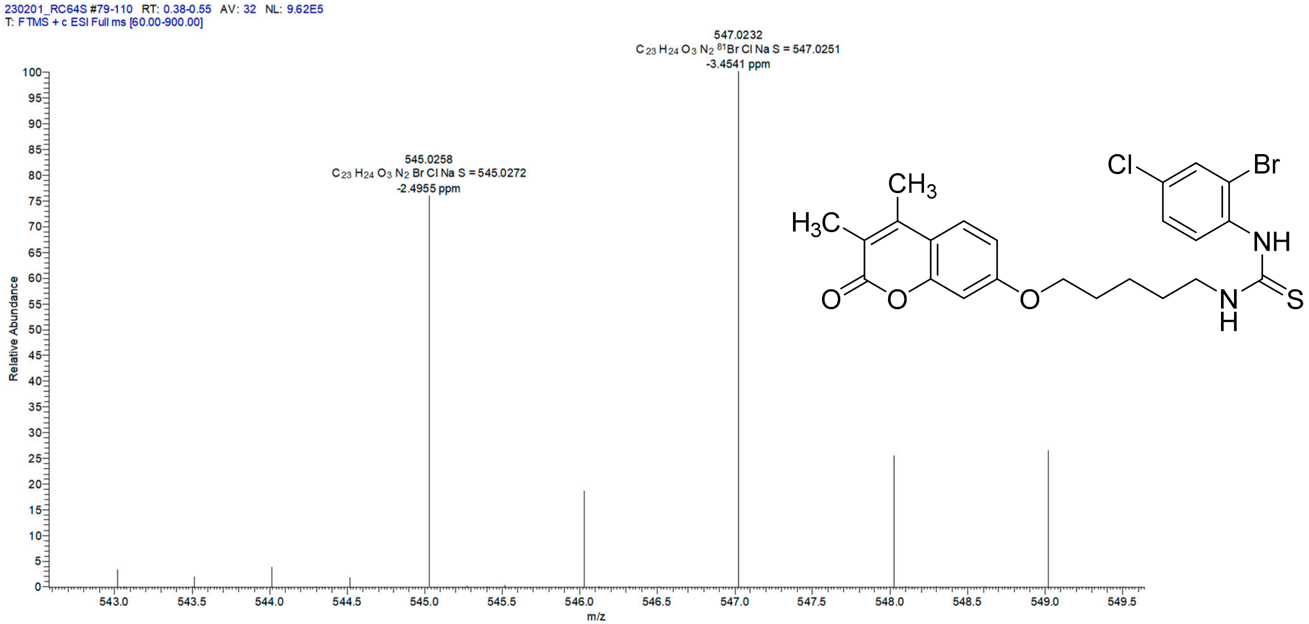

Figure S39. HRESI-MS of **59**

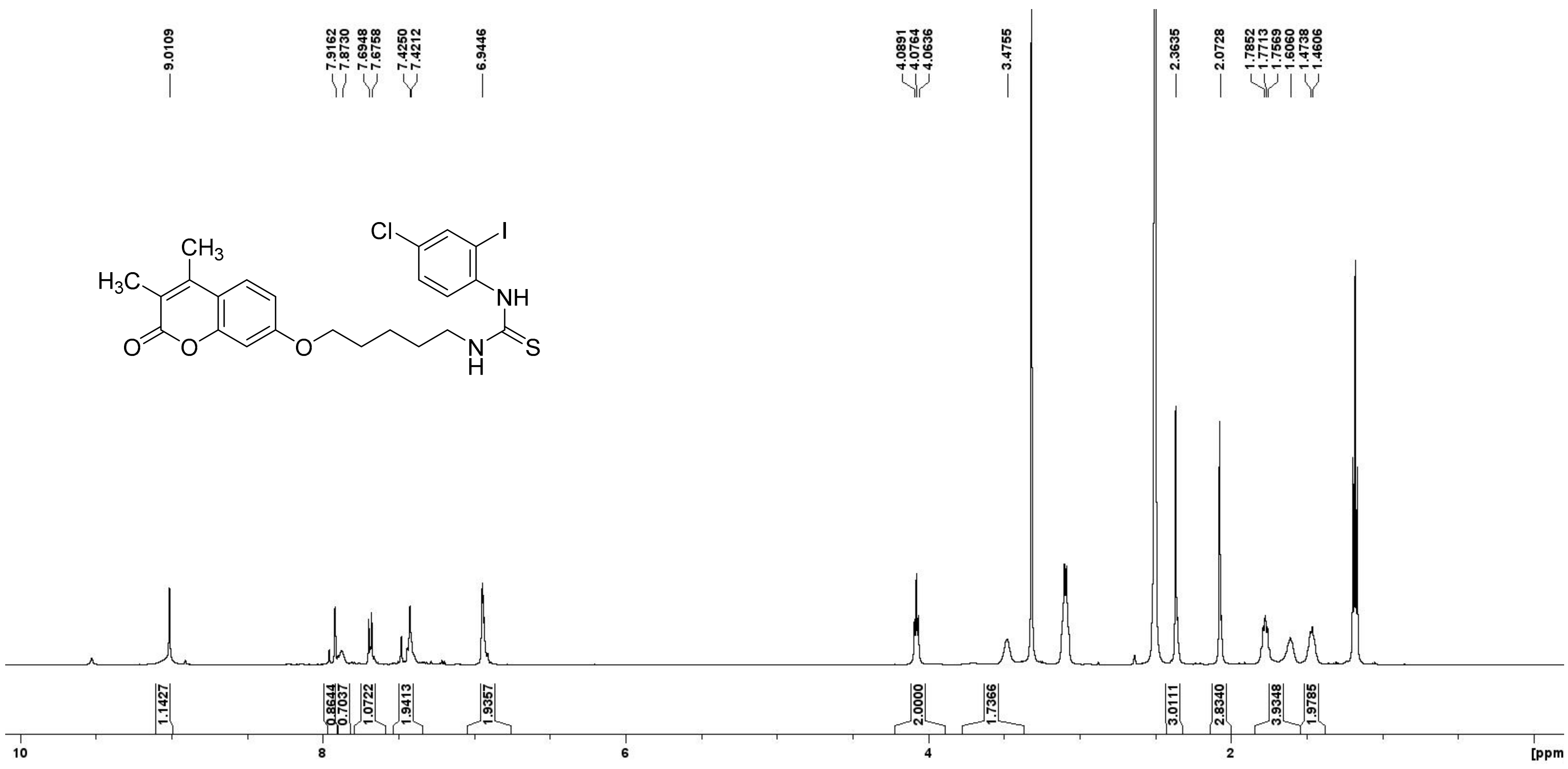

Figure S40. ^{1}H-NMR spectrum (500 MHz, DMSO-d_6) of **60**

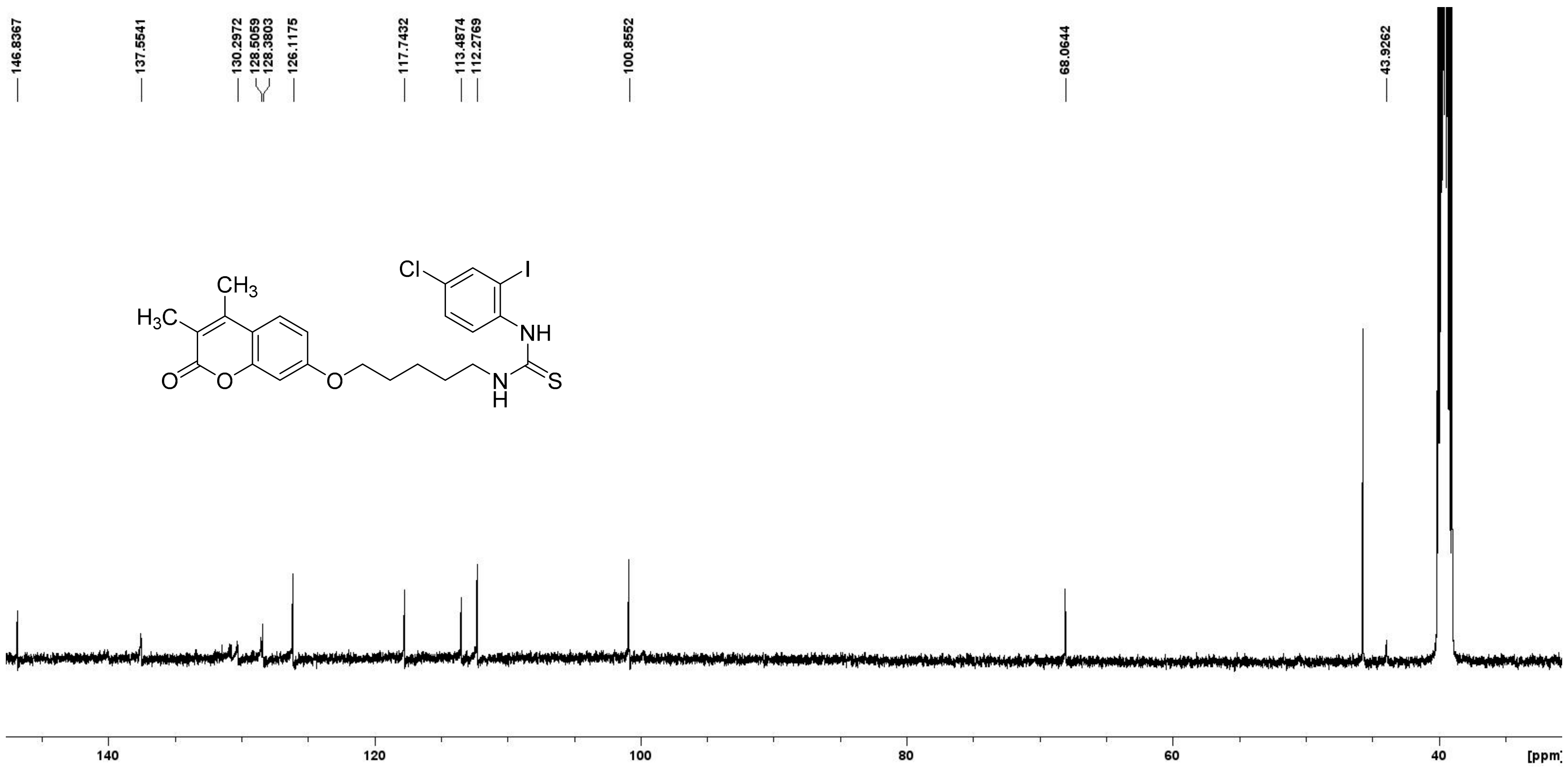

Figure S41. ^{13}C-NMR spectrum (125.7 MHz, DMSO-d_6) of **60**

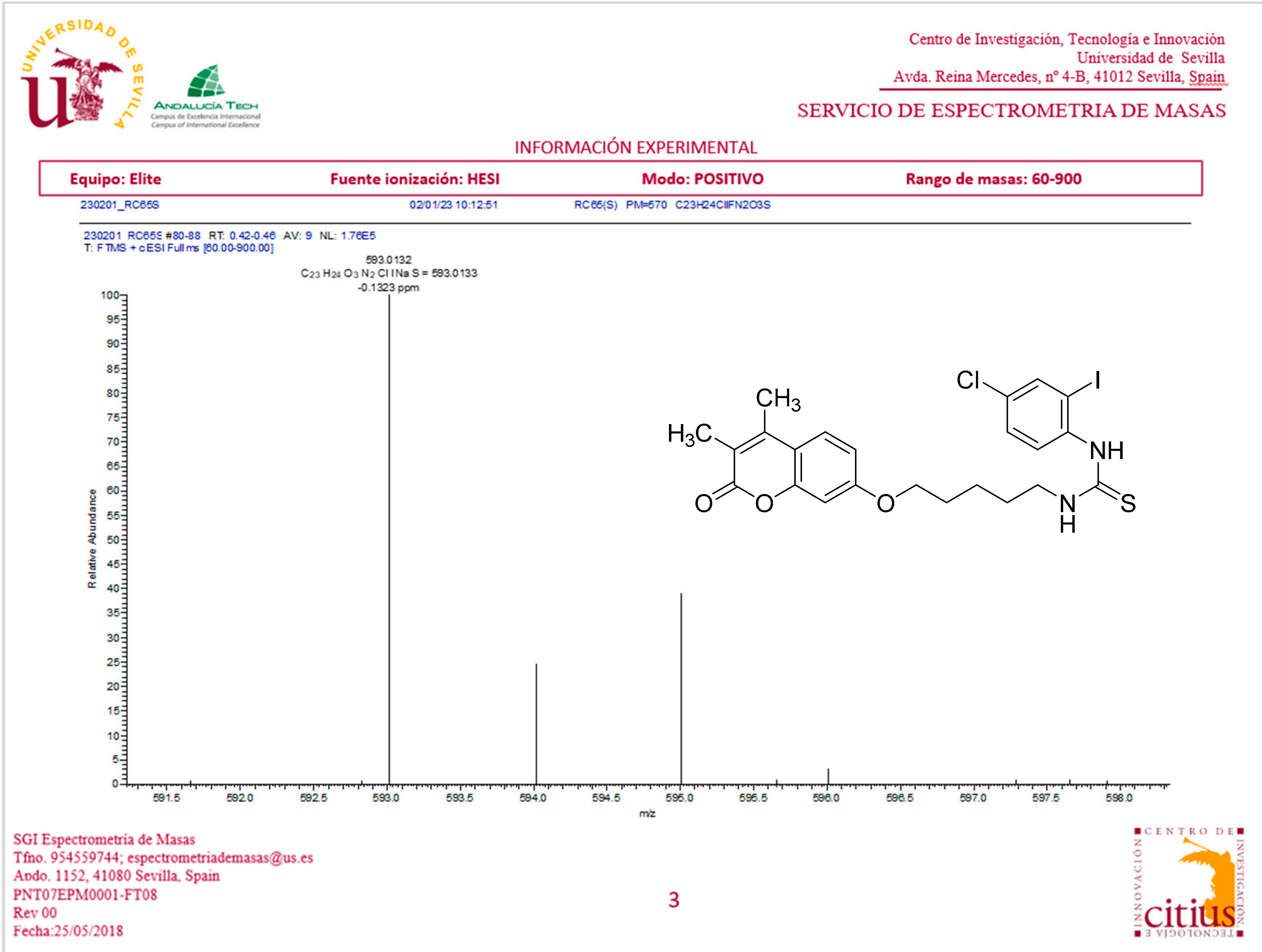

Figure S42. HRESI-MS of **60**
